# Evidence for maternal autoantibodies in the pathogenesis of preterm birth

**DOI:** 10.1038/s41590-026-02689-y

**Published:** 2026-10-14

**Authors:** Elze Rackaityte, Beltran Borges, Hannah M. Kortbawi, Haleigh S. Miller, Shirley J. Shao, Kelsey C. Zorn, Colette Caspar, Rebecca Wong, Frank McCarthy, Joseph S. Creery, Margareta Mayer, Madhura Raghavan, Elaine Kouame, Robert R. Puccinelli, Amy L. Kistler, David J. L. Yu, Sammer Roach Ganem, Alexandra DeRisi, Quinn Kawaja, Chung-Yu Wang, Aditi Saxena, Scott Oltman, Andrew F. Kung, Sabrina A. Mann, Anthea Mitchell, David Huang, Gabrielle Rizzuto, Rebecca J. Baer, Caleigh Mandel-Brehm, Kelli K. Ryckman, Stephanie L. Gaw, Sara L. Hillman, Laura L. Jelliffe-Pawlowski, Joanna Halkias, Nasim C. Sobhani, Mark S. Anderson, Francsesca Trespidi, Claudia Alpini, Tiziana Bosoni, Riccardo Albertini, Camilla Bellingeri, Barbara Gardella, Alessandro Borghesi, Nicole Prince, Jessica Lasky-Su, Michael R. Wilson, Marcelle I. Cedars, Joshua E. Elias, Tippi C. MacKenzie, Joseph L. DeRisi

**Affiliations:** 1 Department of Biochemistry and Biophysics, University of California San Francisco, San Francisco, CA USA; 2 Department of Surgery, University of California San Francisco, San Francisco, CA USA; 3 Center for Maternal-Fetal Precision Medicine, University of California San Francisco, San Francisco, CA USA; 4 The Eli and Edythe Broad Center of Regeneration Medicine and Stem Cell Research, San Francisco, CA USA; 5 Department of Obstetrics and Gynecology and Reproductive Sciences, University of California San Francisco, San Francisco, CA USA; 6 Department of Neurology, UCSF Weill Institute for Neurosciences, University of California San Francisco, San Francisco, CA USA; 7 Chan Zuckerberg Biohub San Francisco, San Francisco, CA USA; 8 Division of Neonatology, Department of Pediatrics, University of California San Francisco, San Francisco, CA USA; 9 UC Berkeley - UCSF Graduate Program in Bioengineering, University of California San Francisco, San Francisco, CA USA; 10 Diabetes Center, School of Medicine, University of California San Francisco, San Francisco, CA USA; 11 Department of Epidemiology and Biostatistics, University of California San Francisco, San Francisco, CA USA; 12 UCSF California Preterm Birth Initiative, San Francisco, CA USA; 13 UCSF Institute for Global Health Studies, San Francisco, CA USA; 14 Immuno-Oncology Program and Department of Pathology and Laboratory Medicine, Memorial Sloan Kettering Cancer Center, New York, NY USA; 15 Department of Pediatrics, University of California San Diego, La Jolla, CA USA; 16 Department of Epidemiology and Biostatistics, School of Public Health-Bloomington, Indiana University, Bloomington, IN USA; 17 Maternal and Fetal Medicine Department, Institute for Women’s Health, University College London, London, UK; 18 Host-Pathogen Interaction Group and Neonatal Intensive Care Unit, Fondazione IRCCS Policlinico San Matteo, Pavia, Italy; 19 Medical Genetics, Department of Molecular Medicine, University of Pavia, Pavia, Italy; 20 School of Life Sciences, Swiss Federal Institute of Technology, Lausanne, Switzerland; 21 Laboratory of Biochemical-Clinical Analyses, Fondazione IRCCS Policlinico San Matteo, Pavia, Italy; 22 Department of Obstetrics and Gynecology, Fondazione IRCCS Policlinico San Matteo, Pavia, Italy; 23 Department of Clinical-Surgical, Diagnostic and Pediatric Sciences, University of Pavia, Pavia, Italy; 24 Neonatal and Pediatric Intensive Care Unit, Mother, Child and Adolescent Department, Geneva University Hospitals, Geneva, Switzerland; 25 Department of Pediatrics, Gynecology and Obstetrics, University of Geneva, Geneva, Switzerland; 26 Channing Division of Network Medicine, Department of Medicine, Brigham and Women’s Hospital and Harvard Medical School, Boston, MA USA

**Keywords:** Antibodies, Pattern recognition receptors, Inflammatory diseases

## Abstract

Preterm birth is associated with breakdown of maternal–fetal tolerance and remains the leading cause of global mortality in young children. However, the role of self-reactive antibodies has been limited to a handful of pathogenic allo- and autoantibodies linked to pregnancy complications. Here we identify a shared autoreactive signature of pregnancy in pregnant sera (*n* = 2,719) relative to never-pregnant controls (*n* = 130), using proteome-wide autoantibody profiling. Across eight human pregnancy cohorts, we define autoreactivities associated with preterm birth by comparing preterm (*n* = 688) and term (*n* = 1,505) pregnancies. Within the signature, antibodies to IL1 receptor antagonist (IL1RA) induce resorption, inflammation and vascular malperfusion in pregnant mice. IL1RA localizes to placental endothelial adherens junctions, suggesting a role in vascular integrity. These findings identify a term pregnancy humoral immune program and shared changes preceding preterm delivery, including anti-IL1RA, which may have direct pathogenic consequences.

## Abstract (web summary)

Preterm labor is linked to breakdown of maternal–fetal tolerance. Rackaityte et al. report that some pregnant mothers harbor maternal autoantibodies, including those against IL-1 receptor antagonist, that may contribute to preterm delivery.

## Main

The maternal immune system is reprogrammed during pregnancy to tolerate a semi-allogeneic fetus, yet the mechanisms by which immune tolerance is established and maintained in normal human pregnancy remain incompletely understood. Failure to establish or maintain maternal–fetal immune tolerance is associated with preterm birth, and complications of prematurity remain the leading cause of death in children under 5 worldwide^1^. While several maternal^2^, fetal^3^ and environmental factors^4,5^ have been associated with preterm birth, the lack of a mechanistic understanding of immune physiology in term pregnancy, and how this physiology is perturbed in preterm birth, hinders the development of effective diagnostics and therapeutics.

Although little is known about the causes of preterm labor, animal studies have demonstrated that certain inflammatory cytokines are sufficient to induce labor^6–8^. Intrauterine injection of inflammatory cytokines, particularly IL1β, rapidly induces labor in nonhuman primates^6,9–12^ and rodents^13–17^. This finding mirrors associations in humans, where elevated inflammatory cytokines are detected in mothers during labor, including spontaneous preterm labor^18–23^. These data position inflammation not only as a correlate of preterm labor, but as a potential effector mechanism linking maternal immune dysregulation to parturition.

Recently, tolerization of maternal B cells through antigen masking on the placenta has been implicated in the establishment of maternal–fetal tolerance^24^. However, our current understanding of the humoral immune system in human pregnancy is largely limited. In normal term pregnancy, anti-paternal HLA antibodies are commonly generated and are often clinically innocuous in first and subsequent pregnancies^25^. Yet, their presence reflects a potentially functional humoral response: anti-HLA antibodies can mediate graft rejection, complicate organ transplantation in women^26^, and have been associated with spontaneous preterm delivery^27^ and placental endothelial complement deposition^28^. In other pregnancy complications, pathogenic auto- and alloantibodies such as anti-RhD^29^, anti-thyroid^30^, anti-human platelet antigen^31^ and anti-AT1 receptor^32^ antibodies have been identified. Together, these observations raise the possibility that dysregulated maternal humoral immunity may contribute to inflammatory pathways that precipitate early delivery.

Here we describe the use of proteome-wide autoantibody profiling technologies to generate a comprehensive portrait of autoimmune reactivities in maternal sera during normal term pregnancy and identify those that specifically precede preterm birth.

## Maternal autoantibody enrichment pregnancy atlas

**Fig. 1:**
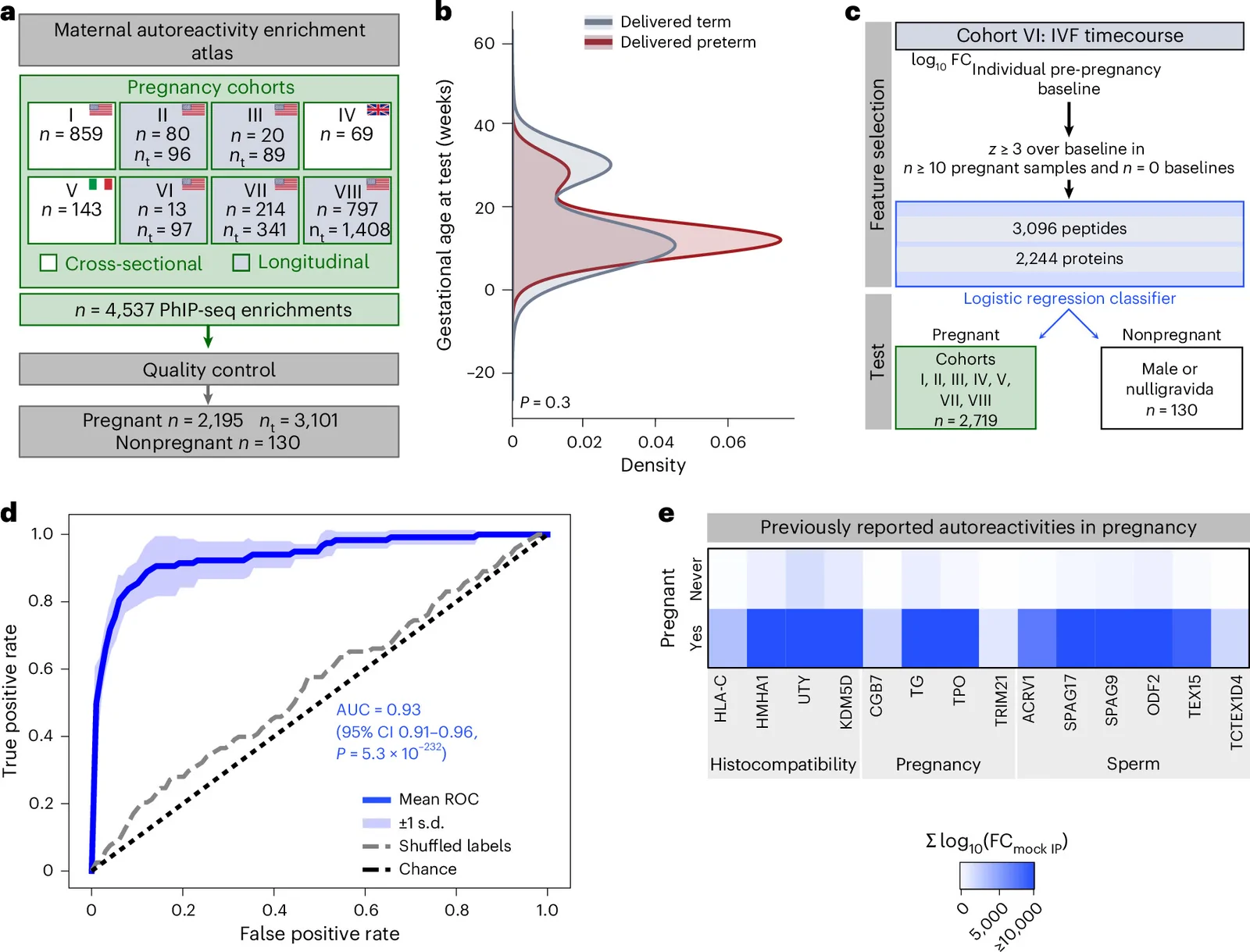
Maternal autoantibody signature induced by becoming pregnant. **a**, Schematic of study design integrating data from eight human pregnancy cohorts of maternal sera human analyzed by PhIP-seq with the human peptidome (*n* indicates human participant counts, *n*_t_ indicates human samples across multiple time points). **b**, Histogram of gestational age at time of sampling in weeks for women that delivered term and preterm across all cohorts tested. **c**, Schematic for identifying the PhIP-seq signature utilizing PhIP-seq data. Feature selection was performed on Cohort VI, where log_10_ fold changes (FC) were taken over individual prepregnancy baselines and *z*-scores were calculated over this baseline. Peptides were considered pregnancy-specific if they were detected in ten pregnant samples and zero baselines. These features were used to train a fivefold cross-validated logistic regression classifier to discriminate pregnant (cohorts I–V and VII–VIII) and nonpregnant samples. **d**, ROC curve showing fivefold cross-validated performance of features selected from cohort VI applied to pregnant samples (cohorts I–V and VII–VIII) and nonpregnant controls. Solid lines represent the mean ROC across 2,000 bootstrap resamples; shaded regions denote ±1 s.d.; 95% CIs are bootstrap percentile intervals (2,000 iterations). Two-sided *P* value from DeLong one-sample test versus AUC = 0.5. The dotted gray line indicates performance after label shuffling. **e**, Sum fold change over mock IP of selected proteins in the PhIP-seq pregnancy signature in pregnant or never-pregnant samples. Mann–Whitney *U* test for significance in **b**. Source data

To comprehensively investigate autoreactivity during pregnancy, maternal sera from term and preterm pregnancies derived from eight separate cohorts across three countries were used (*n* = 3,101 samples derived from *n* = *x* pregnant individuals). The cohorts captured cross-sectional (I, IV and V), longitudinal (II, III and VI–VIII) and population-based (I) designs (Fig. 1a). Cohorts III and VI comprised only term pregnancies with dense sampling across gestation. Paired maternal–cord blood samples were available for cohorts III and IV (Supplementary Fig. 1a–d and Supplementary Table 1). Gestational age at sample collection was highly similar between preterm and term outcomes (Fig. 1b, Supplementary Table 1 and Supplementary Fig. 1a,b). A separate cohort of sera from healthy males and nulligravid females was used as a nonpregnant control group (*n* = 130; Supplementary Table 1). Serum from these cohorts was used to immunoprecipitate a customized, previously validated programmable phage display library of the human proteome with the phage immunoprecipitation (IP) sequencing (PhIP-seq) approach^33,34^. In total, 4,537 PhIP-seq enrichments were performed, encompassing positive and negative control IPs as well as technically replicated human samples (Supplementary Table 1).

**Extended Data Fig. 1:**
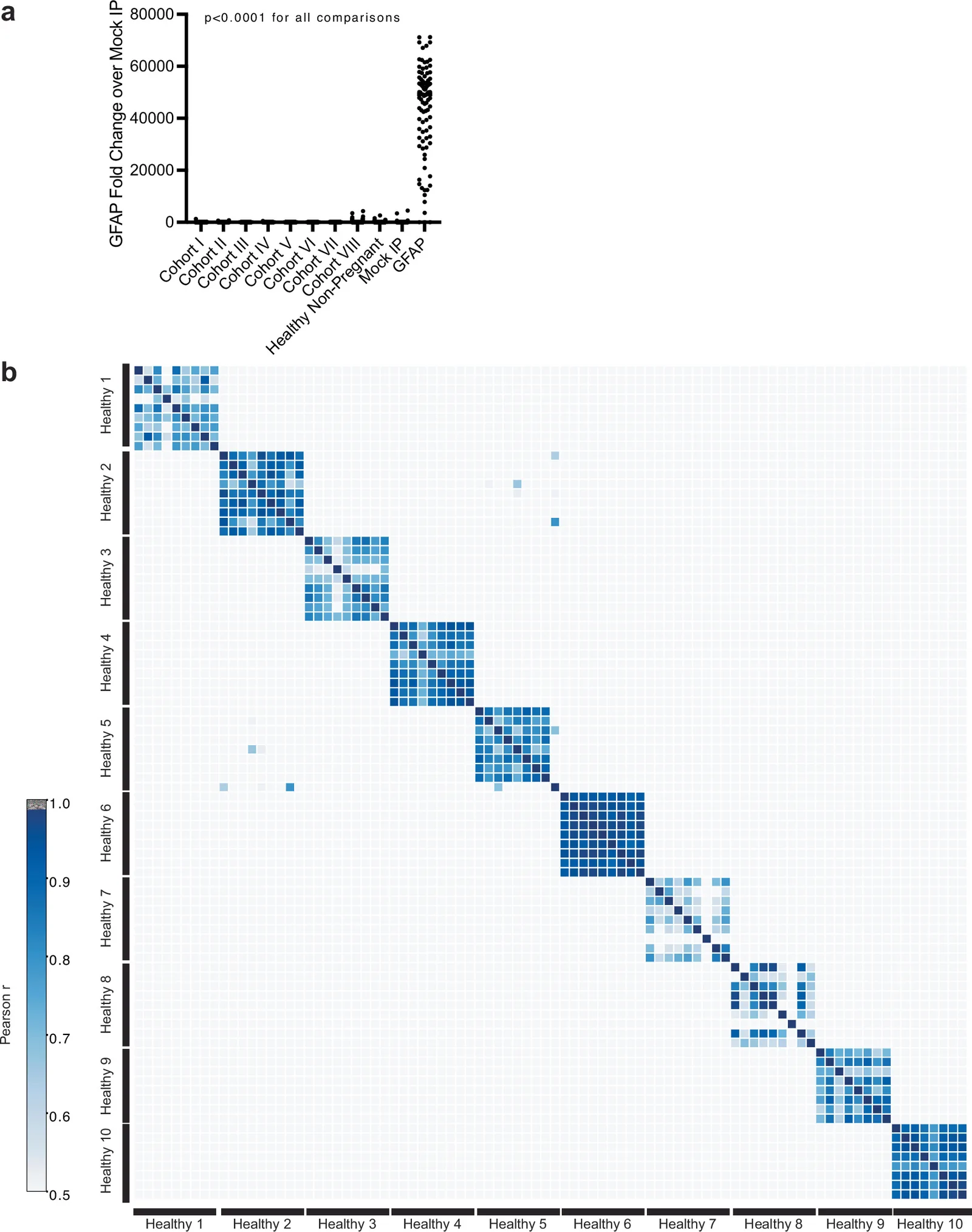
Technical consistency of the PhIP-seq method. **a**. Sum reads per 100,000 (RPK) of peptides tiling across the GFAP protein in n = 4,537 immunoprecipitations (IPs) with sera from Cohorts I-VII, healthy non-pregnant samples, mock IP, and anti-GFAP antibody. **b**. Pearson co-correlation analysis of fold change over mock IP across n = 10 technically replicated samples across 20 plates. Enrichments identified by PhIP-seq in **a**-**b**. Each dot represents an independent IP in **a**. Two-way ANOVA test for significance with Tukey post-hoc in **a**. Source data

Following three rounds of phage enrichment, the encoded peptide inserts were sequenced and aligned to the input library to calculate overall enrichment as previously described^33^ (Supplementary Methods). A commercial polyclonal antibody against glial fibrillary acidic protein (GFAP) was used as a positive control and replicated across 96-well plates, batches and cohorts 88 times. Peptides corresponding to the GFAP protein were enriched by an average of 10^3^-fold relative to IPs with sera from the human cohort samples (Extended Data Fig. 1a). Before any filtering, PhIP-seq enrichments from nonpregnant healthy controls were investigated for batch effects. A subset of samples (*n* = 10) was technically replicated nine times in random positions across 20 experimental plates. Each set of ten technical replicates exhibited high Pearson co-correlation, and as has been previously observed for immune profiling of healthy controls^35^, comparison of individuals with each other yielded low to no correlation (Extended Data Fig. 1b). After quality-control filtering to remove under-sequenced samples and averaging technically replicated samples (Supplementary Table 1 and Supplementary Methods), a total of *n* = 3,101 samples derived from *n* = 2,195 pregnant participants and *n* = 130 samples from healthy nonpregnant controls were retained. Because peptide-level representation in the human proteome-wide library can collapse shared sequence features across self and non-self allelic variants, we use the term autoantibody to describe maternal antibody reactivities against human protein-derived peptides, while recognizing that some of these signals may reflect alloantibody reactivity. Thus, we generated PhIP-seq profiles across 3,101 maternal serum samples from 2,195 pregnancies spanning eight diverse cohorts, alongside nonpregnant controls, resulting in a rigorously quality-controlled, proteome-wide map of maternal autoantibody reactivity across pregnancy outcomes.

## Global remodeling of autoreactivity during pregnancy

**Extended Data Fig. 2:**
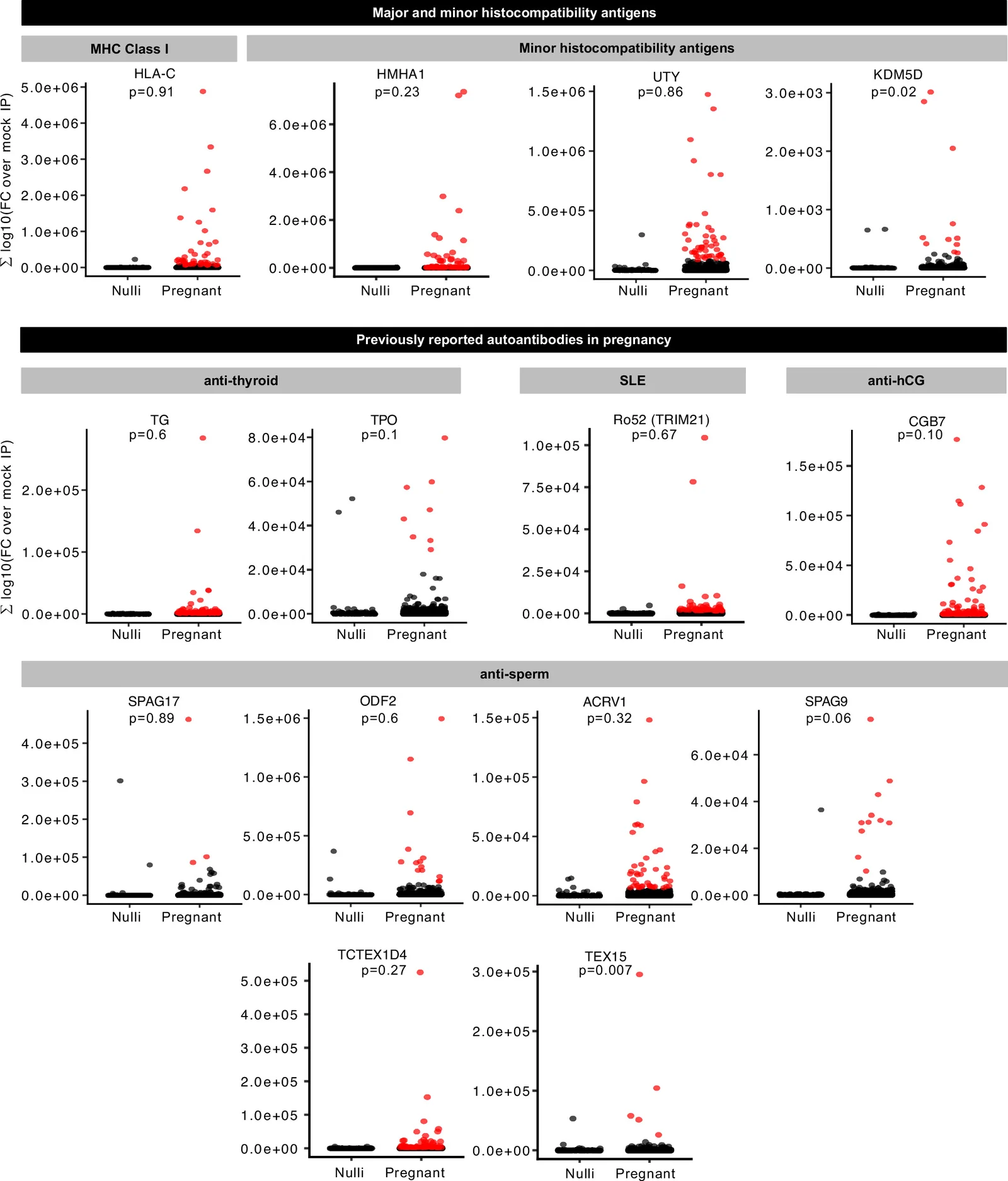
Autoreactivities enriched in pregnancy. Dot plots of summed log10 fold change of mock IP within an individual for peptides belonging to proteins in indicated categories in nulligravid (nulli) or pregnant sera. Red dots denote greater than 3 standard deviations from non-pregnant experiments. Individual human samples are represented by each dot, two-way Kolmogorov-Smirnov pairwise test for significance. Source data

During pregnancy, the maternal immune system must adapt to accommodate the semi-allogeneic fetus. To first assess the induced proteome-wide autoreactivity that accompanies normal pregnancy, we analyzed a cohort of term pregnancies resulting from frozen embryo transfer (FET; Cohort VI) with sampling pre-FET, across gestation, and postpartum. Peptide-level fold changes were computed relative to each individual’s prepregnancy baseline. Pregnancy-specific peptides were defined as those with *z*-score ≥3, present in ≥10 pregnancy samples and absent from all baseline samples (Fig. 1c). To derive the common signature of autoreactivity during normal pregnancy, the resulting 3,096 peptides, from 2,244 proteins, were used to build a logistic regression classifier (Supplementary Methods). The classifier was then used to distinguish pregnant (*n* = 2,719) from nonpregnant (*n* = 130) samples from all cohorts except cohort VI, which was used during feature selection only. The mean area under the curve (AUC) of the receiver-operating-characteristic (ROC) analysis was 0.93 (95% confidence interval (CI) 0.91–0.96, *P* = 5.3 × 10^−232^; Fig. 1d), supporting the existence of a large set of shared autoreactive pregnancy-associated changes. The PhIP-seq pregnancy signature recapitulated previously reported reactivities to histocompatibility molecules, pregnancy-associated proteins and sperm antigens (Fig. 1e) and identified autoreactive targets not previously known to be pregnancy associated (Extended Data Fig. 2 and Supplementary Table 2). Together, these data define a complex yet highly shared set of autoreactive changes in the pregnant mother.

## The preterm autoantibody signature

**Fig. 2:**
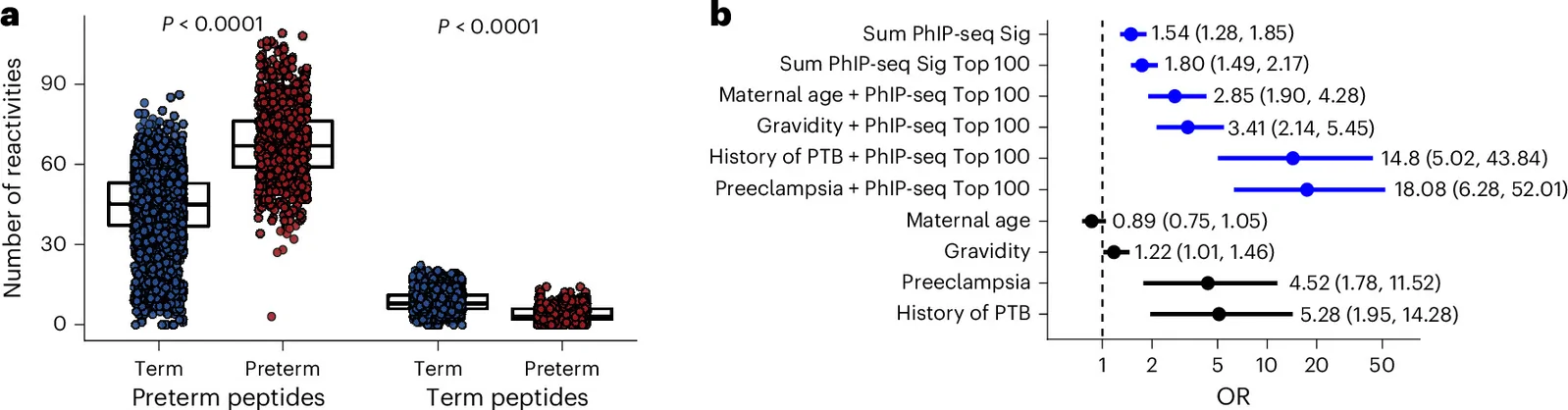
The preterm autoantibody signature. **a**, Number of preterm- and term-specific autoantibody reactivities per individual (*n* = 2,278) in term (blue) and preterm (red) pregnancies across all cohorts. Each point represents one individual; boxes indicate median and interquartile range; whiskers extend to minimum and maximum values. One-way ANOVA with Tukey honestly significant difference (HSD) test for significance. **b**, Forest plot of univariate and combined ORs for preterm birth. ORs and 95% CIs were estimated using logistic regression models predicting preterm birth. Clinical variables (history of preterm birth (PTB), preeclampsia, maternal age and gravidity) and PhIP-seq antibody enrichment metrics (total signature hits and top 100 ranked peptides) were evaluated in separate univariate models. Points indicate OR estimates, and horizontal whiskers indicate 95% CIs. Combined risk estimates represent the joint effect of clinical variables and the PhIP-seq top 100 peptide score from a multivariable logistic regression model, evaluated at +2 s.d. of the PhIP-seq score. Variance for the combined estimates was calculated using the full covariance matrix of the multivariable model. The vertical dashed line indicates the null value (OR 1). Samples with missing clinical variables were excluded. Source data

**Extended Data Fig. 3:**
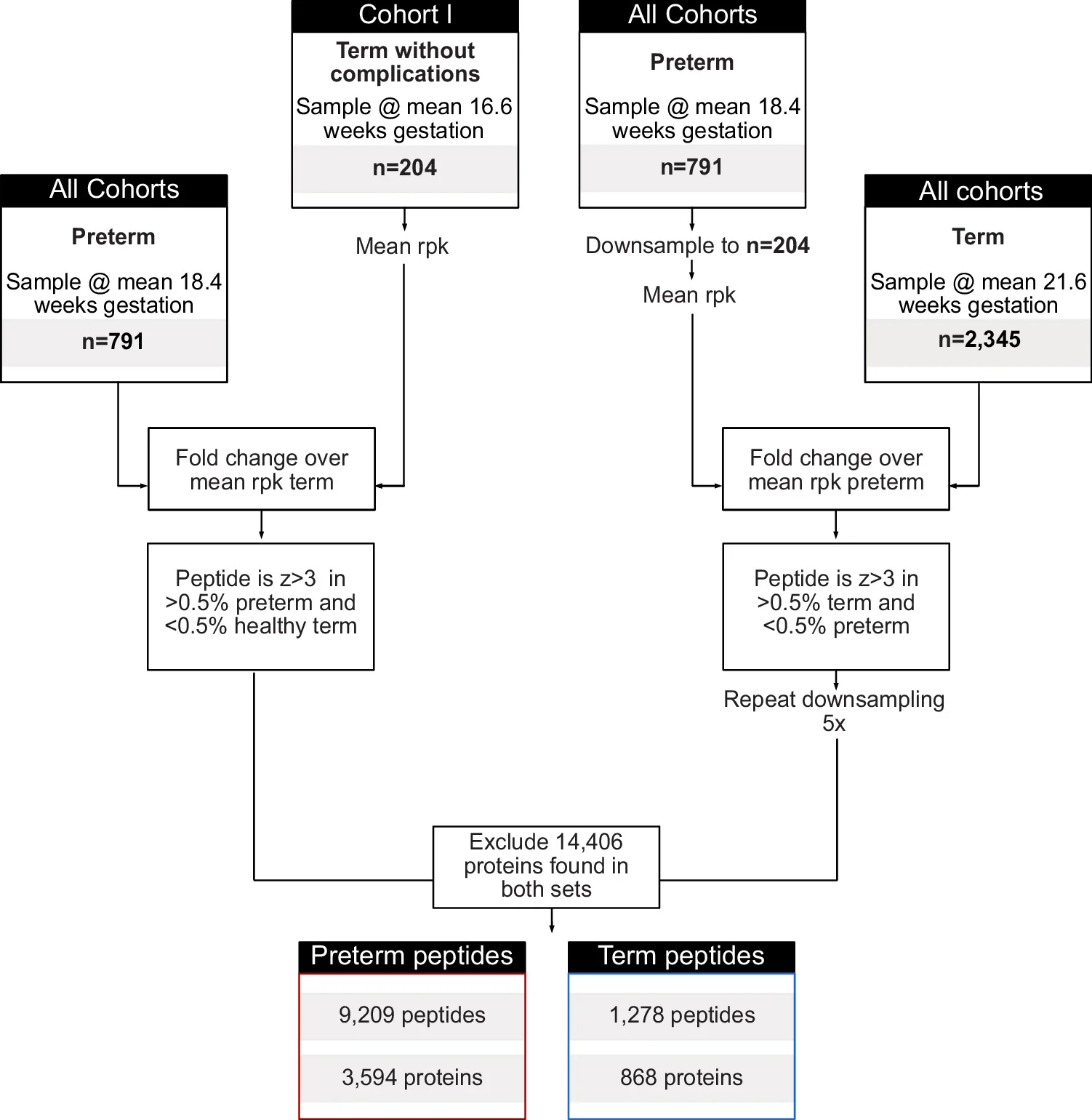
Identification of term- and preterm-specific autoreactivities by PhIP-seq. Flow chart of hit filtering strategy from input peptide library to term and preterm specific hits.

**Extended Data Fig. 4:**
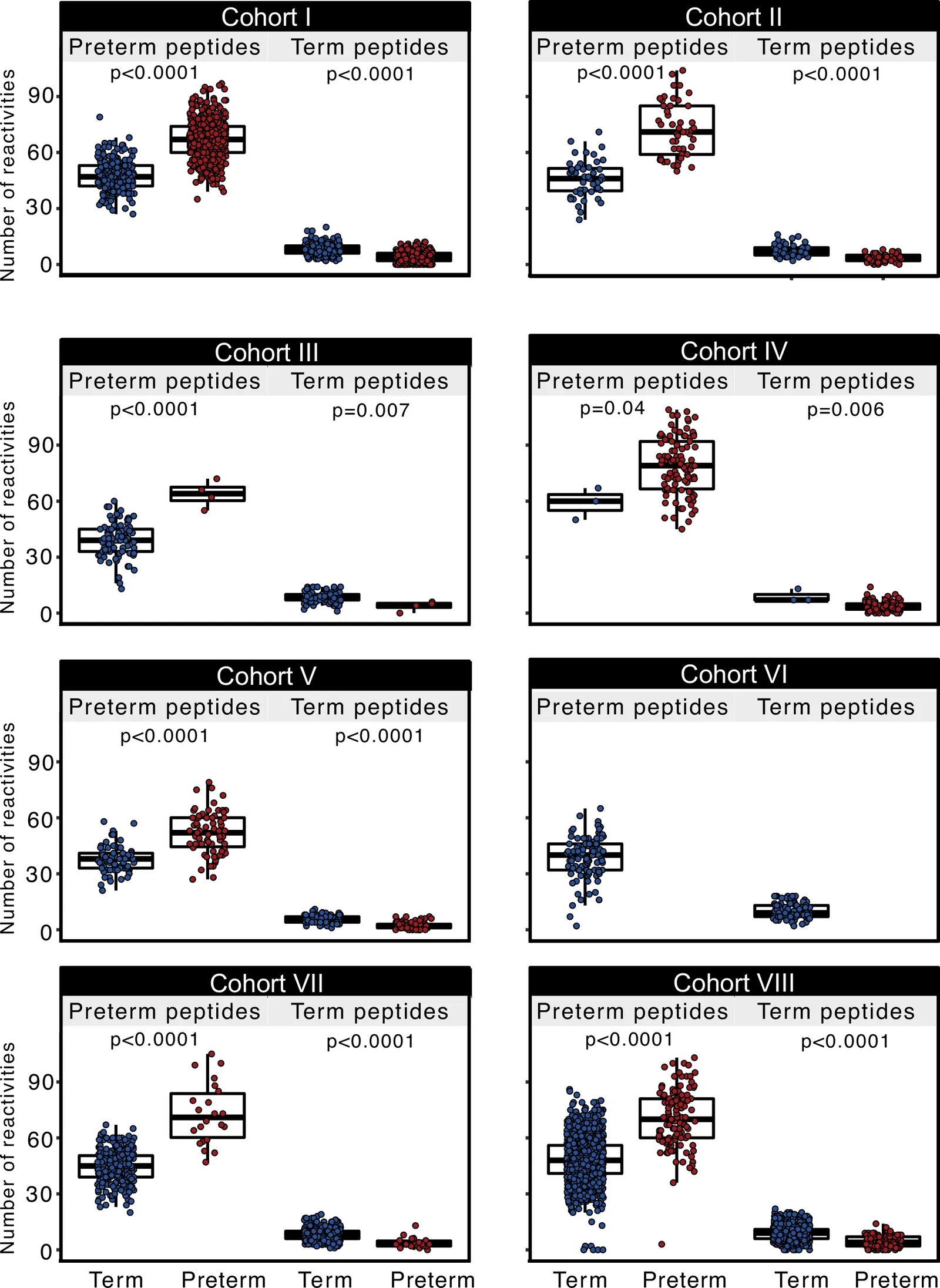
Preterm autoantibody signature across cohorts. Number of preterm (left) and term (right) specific autoantibody reactivities in term (blue) and preterm (red) pregnancies across all cohorts tested. Individual human samples are represented by each dot, healthy term pregnancies from Cohort I were excluded from plotting because the analysis was scaled to this reference group. Two-way Kolmogorov-Smirnov pairwise test for significance. Exact n per cohort is reported associated Source Data file; boxes indicate median and interquartile range; whiskers extend to minimum and maximum values. Source data

**Extended Data Fig. 5:**
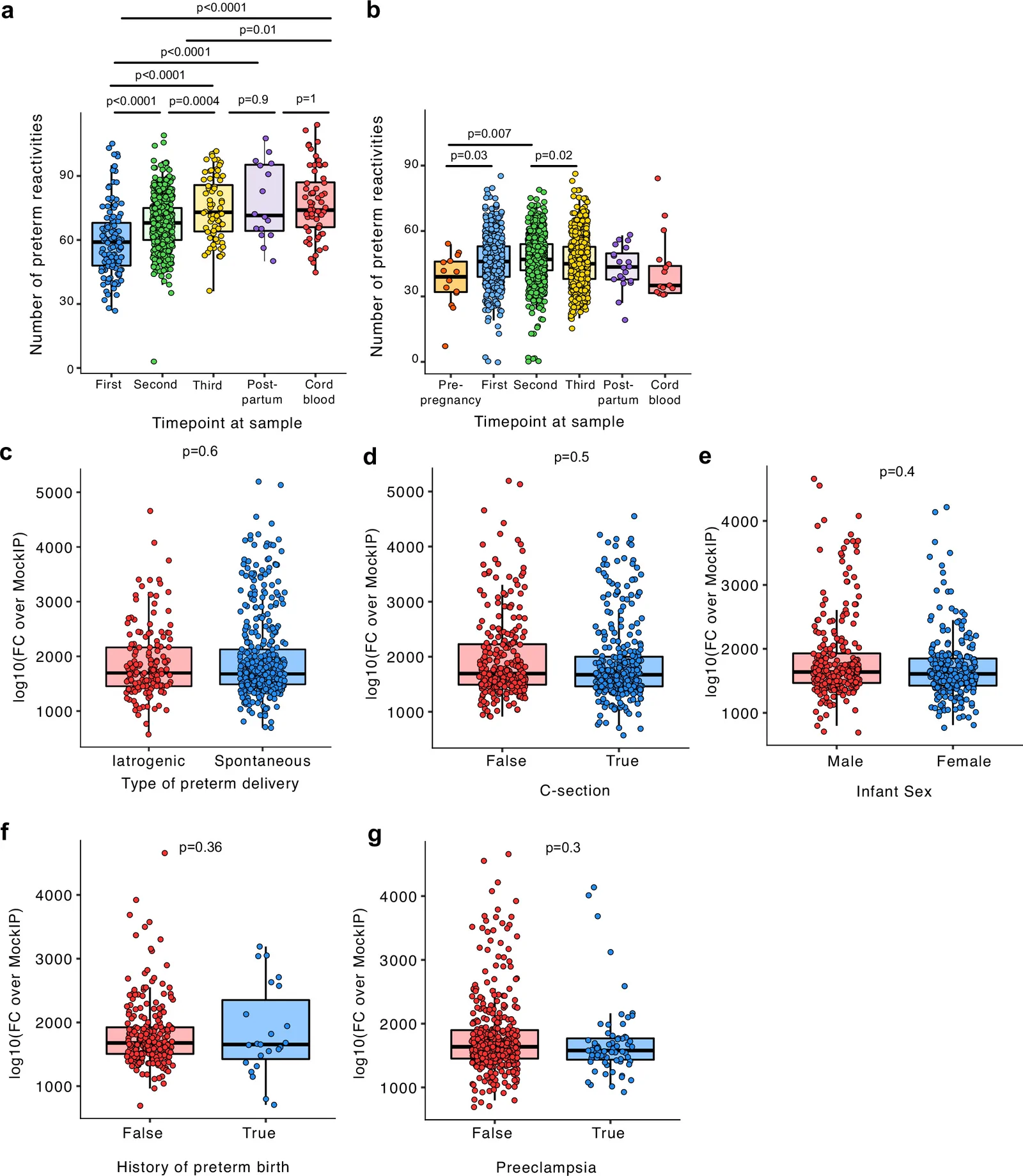
Preterm autoantibody signature with respect to clinical variables. Boxplot of the number of preterm reactivities per person among **a**. n = 791 preterm or **b**. n = 2,141 term pregnancies, grouped by timepoint of collection. Summed log10 fold change over mock IP of the preterm autoantibody signature in individuals with preterm pregnancies stratified by **c**. type of preterm delivery n = 574, **d**. delivery mode n = 574, **e**. infant sex n = 497, **f**. history of preterm birth n = 257, **g**. preeclampsia diagnosis n = 456. One-way ANOVA with Tukey HSD in a-b, two-way Kolmogorov-Smirnov pairwise test in c-g for significance. PhIP-seq enrichments from individual human samples are represented by each dot. Participants for which clinical variables were not available were excluded from the analysis. Boxes indicate median and interquartile range; whiskers extend to minimum and maximum values. Source data

We next assessed whether preterm pregnancies exhibit a shared autoreactive signature distinguishable from term pregnancy. A set of healthy term pregnancies from cohort I was selected as the control group (*n* = 204; Methods) and used to calculate mean reads per 100,000 (RPK) values at the peptide level. Subsequently, peptide-level fold changes were calculated over the control means for all pregnancy cohorts. Peptides with a *z*-score of 3 or greater were designated as preterm-enriched if they were shared by at least four preterm participants and were enriched in one or less of the 204 healthy term participants (*z*-score ≤3; Extended Data Fig. 3 and Supplementary Methods). Conversely, term-specific peptides were identified using the mean of the preterm samples (down-sampled to *n* = 204) to calculate *z*-scores (Supplementary Methods). This analysis yielded a total of 9,209 peptides derived from 3,594 proteins that were labeled as preterm-specific and 1,278 peptides derived from 868 proteins that were labeled as term-specific (Supplementary Table 3). The number of reactivities within the preterm autoantibody signature was significantly greater in preterm than term individuals. Conversely, the number of reactivities within the term autoantibody signature was overall lower, but still significantly higher in term than preterm pregnancies (Fig. 2a). This difference in enrichments was consistent across all eight cohorts (Extended Data Fig. 4). While the progression of normal pregnancy is associated with an increase in autoreactivity, the autoreactive enrichments derived from preterm individuals were both distinct and more numerous. The breadth of the preterm signature was also found to be significantly increased as a function of trimester at the time of sampling in preterm but not term pregnancies (Extended Data Fig. 5a,b), with clear divergence already apparent in the first trimester.

To determine whether the preterm autoantibody signature is associated with preterm birth risk relative to established obstetric risk factors, a logistic regression analysis was performed was performed (Fig. 2b). Higher antibody signal was associated with higher odds of preterm delivery (odds ratio (OR) 1.54 per standard deviation (s.d.) increase of summed log fold change over mock IP, 95% CI 1.28–1.85). To assess the strength of the association as a function of the number of autoantibody signature features, the feature set was restricted from as few as 10 peptides to as many as 500 peptides. Even with only 10 peptide features, the ORs were still significant (OR 1.32, 95% CI 1.11–1.56), and by 100 features, the maximum OR was achieved (OR 1.80, 95% CI 1.49–2.17). Established obstetric risk factors showed the expected direction and magnitude: prior preterm birth (OR 5.28, 95% CI 1.95–14.28) and preeclampsia (OR 4.52, 95% CI 1.78–11.52) were each associated with increased risk. By contrast, maternal age (OR 0.89, 95% CI 0.75–1.05) and gravidity (OR 1.22, 95% CI 1.01–1.46) per s.d. were not strongly associated with preterm birth risk. Notably, the autoantibody signature and clinical history provided largely independent risk information: in a multivariate model, patients with both prior preterm birth and elevated autoantibody burden (+1 s.d.) exhibited substantially increased odds (OR 8.36, 95% CI 2.97–23.52) compared with those with neither risk factor, consistent with additive effects on the odds scale.

**Fig. 3:**
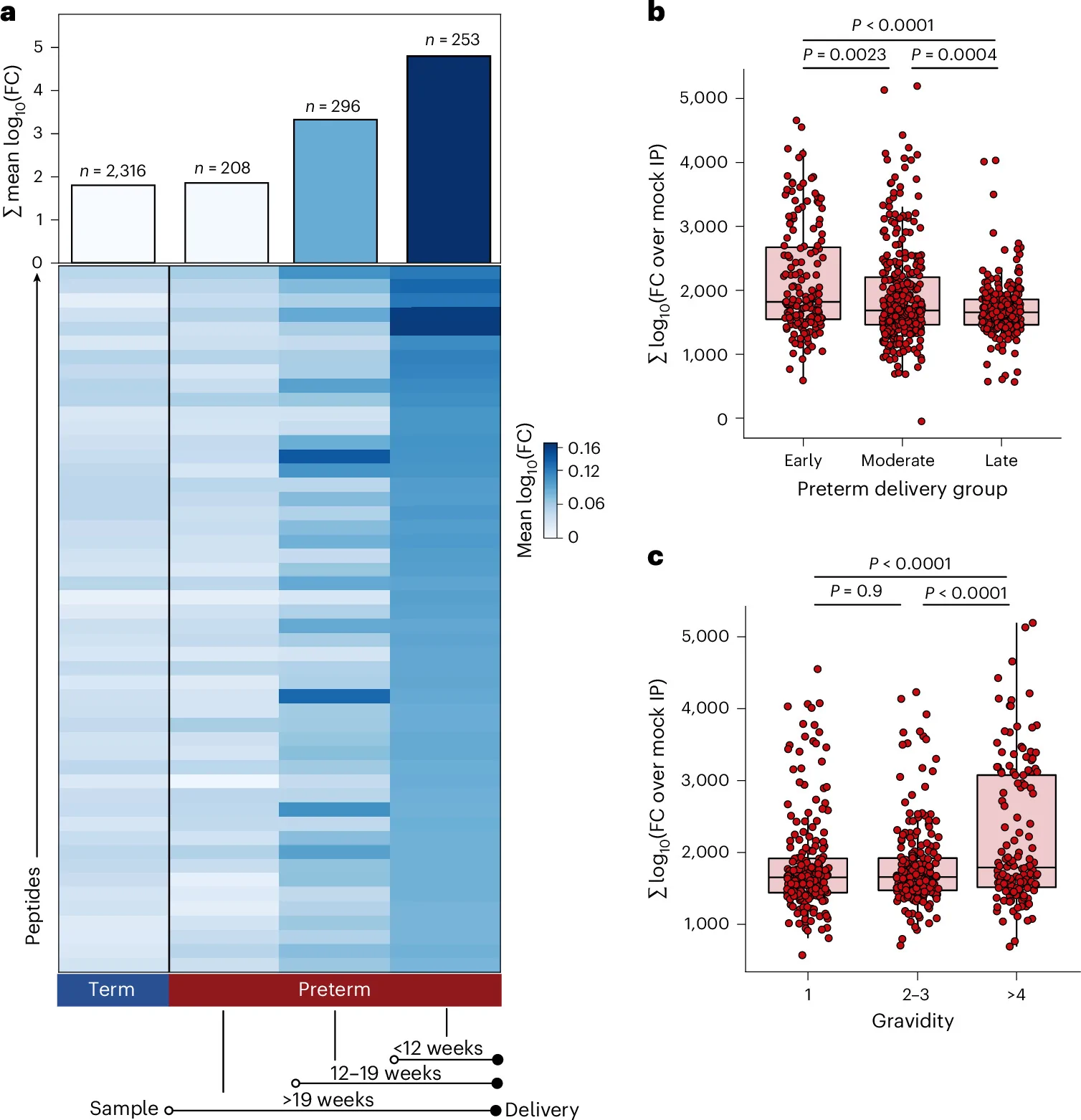
Association of clinical variables with the preterm autoantibody signature. **a**, Heatmap colored by log_10_ fold change over healthy term pregnancies for the top 50 peptides in the preterm autoantibody signature in term or preterm pregnancies sampled >19, 12–19 and <12 weeks before delivery. The bar plot (top) indicates sum of positivity across all peptide means. **b**,**c**, Summed log_10_ fold change over mock IP of the preterm-specific autoantibody signature in preterm pregnancies. Early (<28 weeks), moderate (28–32 weeks) and late (33–37 weeks) delivery age groups of *n* = 727 individuals (**b**) and gravidity categories of *n* = 555 individuals (**c**). Each point represents one individual; boxes indicate median and interquartile range; whiskers extend to minimum and maximum values; one-way ANOVA with Tukey HSD in **b** and **c**; PhIP-seq enrichments from individual human samples are represented by each dot. Participants for whom clinical variables were not available were excluded from the analysis. Source data

We next assessed whether specific clinical features of preterm delivery were reflected within the preterm autoantibody signature. The preterm autoantibody signature became progressively elevated within 19 weeks preceding preterm birth, indicating that the response emerges and intensifies in the months leading up to delivery (Fig. 3a). Furthermore, summed enrichment of the preterm autoantibody signature was significantly greater in more severe preterm deliveries (before 28 weeks) as compared with moderate or late preterm deliveries (Fig. 3b). Multigravidae, and especially women with four or more previous pregnancies, exhibited significantly higher autoreactivity than primigravidae (Fig. 3c). Together, these findings indicate that the preterm autoantibody signature exhibits both a phenotype-severity effect and a cumulative pregnancy–dosage effect, in which repeated pregnancy exposure amplifies the autoreactive response.

**Extended Data Fig. 6:**
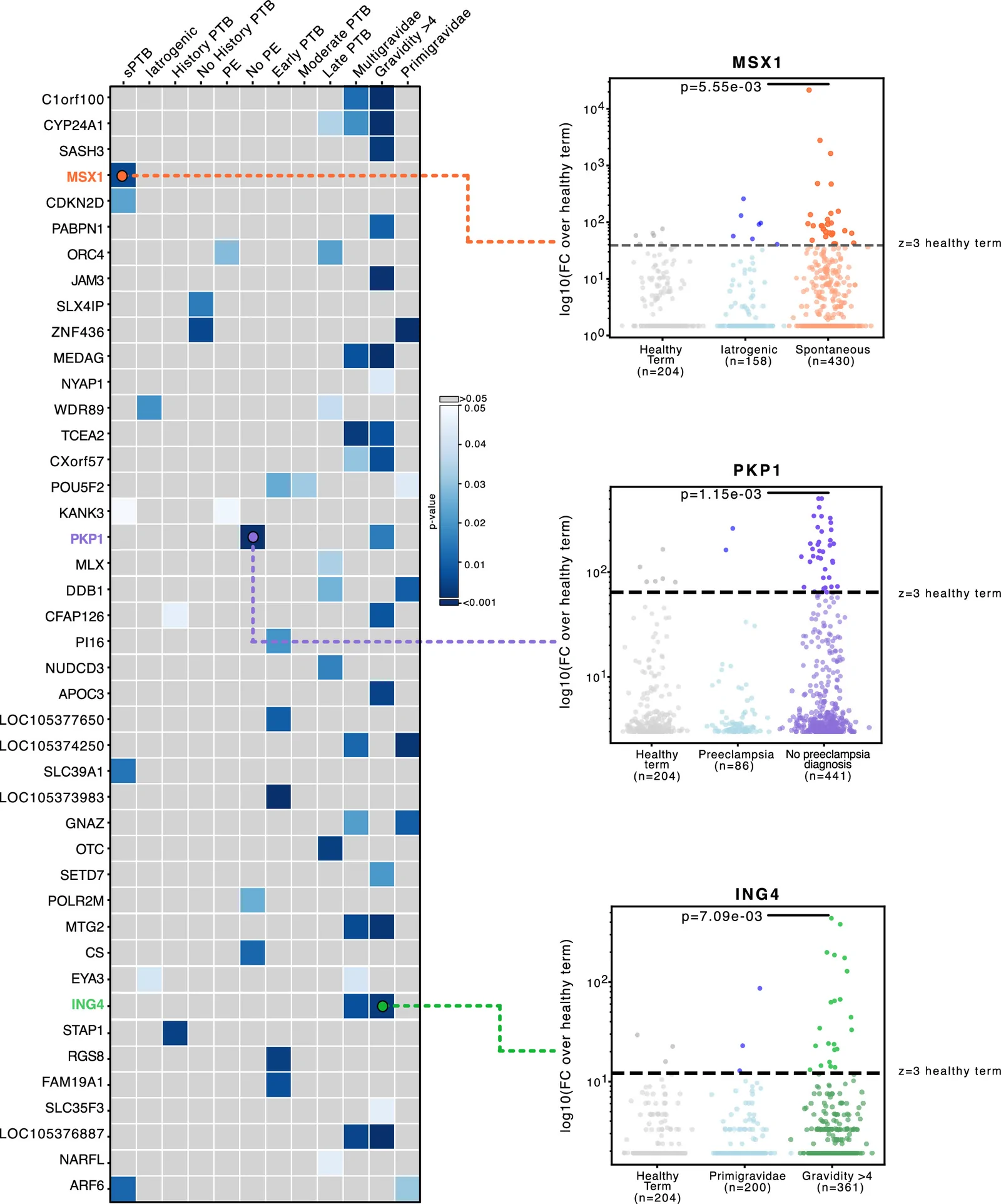
Phenotype–protein association analysis within preterm pregnancy. Heatmap of P values (two-way Kolmogorov–Smirnov testing) for differences in protein-level enrichment between preterm birth subgroups defined by clinical phenotypes. Protein enrichment was calculated as the sum of PhIP-seq enrichment values across significant peptides mapping to each gene. Cells are colored by raw P value for the group with higher mean enrichment; nonsignificant results (P ≥ 0.05) or associations in the opposite direction are masked (gray). Inset, jitter plots of protein-level enrichment distributions for representative proteins, with healthy term pregnancies shown as the reference group with two-way Kolmogorov-Smirnov test results. The dashed line indicates the enrichment threshold defined as the reference mean + 3 s.d. Source data

The preterm autoantibody signature was assessed for relationships with other pregnancy-related characteristics within preterm samples. Spontaneous preterm pregnancies exhibited a modestly higher preterm autoantibody signature enrichment than iatrogenic preterm pregnancies, although this did not reach statistical significance (Extended Data Fig. 5c). C-section delivery, infant sex, history of preterm birth and preeclampsia diagnosis did not significantly associate with the preterm autoantibody signature (Extended Data Fig. 5d–g). However, within-preterm comparisons revealed subtype-specific autoreactive peptide associations, with distinct enrichment profiles distinguishing clinical subtypes (Extended Data Fig. 6 and Supplementary Methods). For example, MSX1 was enriched in spontaneous versus iatrogenic preterm births, PKP1 in pregnancies without preeclampsia and ING4 in high-gravidity mothers (Extended Data Fig. 6), suggesting that the preterm autoantibody landscape reflects heterogeneous clinical etiologies.

**Fig. 4:**
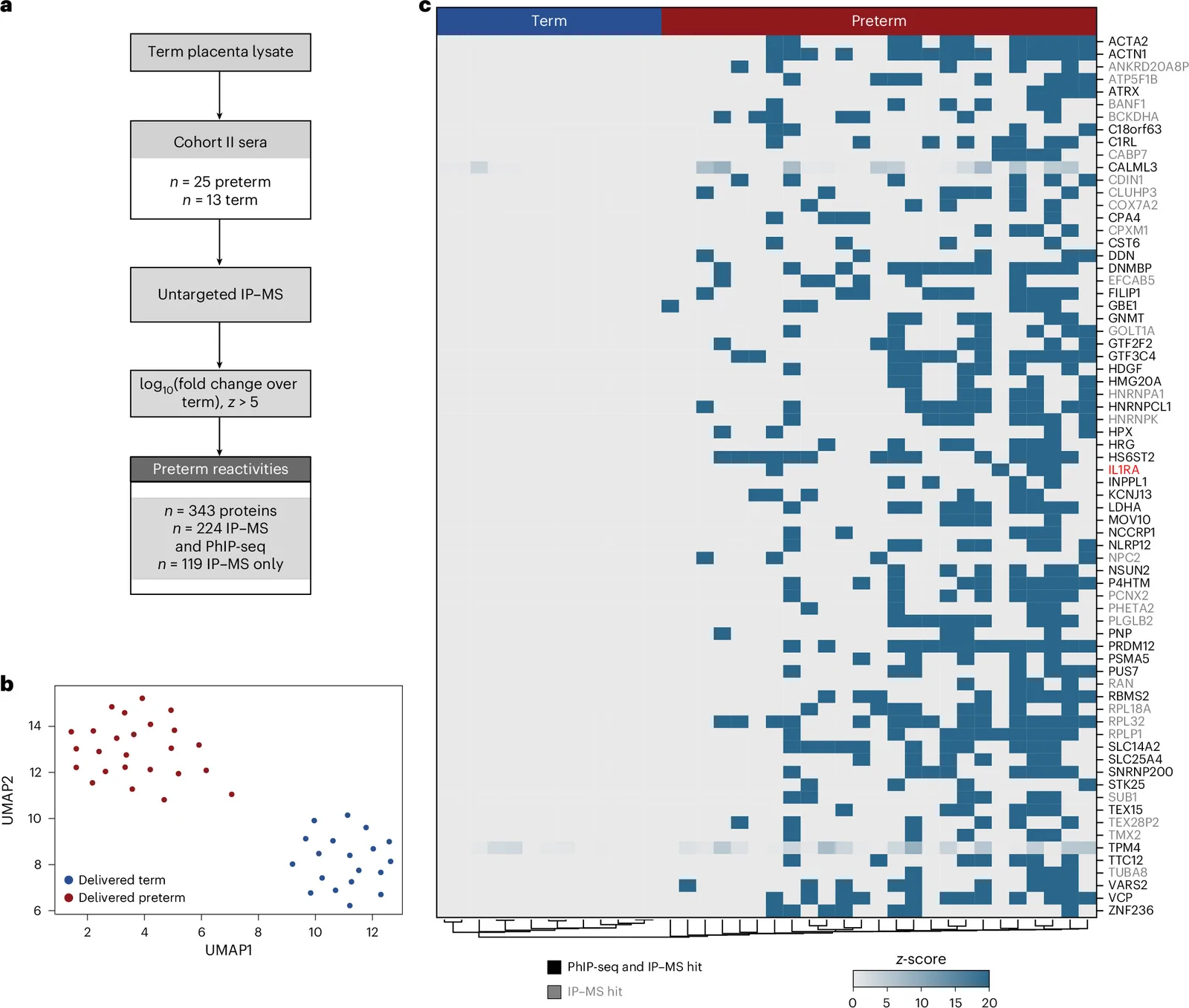
Validation of preterm-specific autoantibody signature. **a**, Schematic of the placental IP–MS experiment and hit filtration approach **b**, Unsupervised UMAP analysis of placental IP–MS protein abundances in cohort II, colored by subsequent term (blue) or preterm (red) delivery. **c**, Heatmap of hierarchically clustered *z*-scores from top 70 preterm-specific reactivities by placental IP–MS. Bolded protein names indicate that the hit was also identified by PhIP-seq in cohort II, while gray protein names indicate that the hit was identified by IP–MS only. Source data

To orthogonally assess the presence of autoreactive antibodies in preterm pregnancy, a randomly selected subset of sera from cohort II (*n* = 13 term, *n* = 25 preterm) was used to immunoprecipitate native proteins from placental extract. Antibody targets were identified using trapped ion mobility time-of-flight mass spectrometry (MS; Methods). MS-derived protein abundances were used to calculate *z*-scores relative to term pregnancies (Methods; Fig. 4a). A total of 343 preterm proteins were identified by IP–MS (Fig. 4a; Methods). While PhIP-seq displays linear peptides, IP–MS may also capture full-length proteins and conformational epitopes. Despite this difference, 65% (*n* = 224 proteins) were also found by PhIP-seq in this cohort (Fig. 4a and Supplementary Table 5). A Uniform Manifold Approximation and Projection (UMAP) analysis of the enriched protein abundances yielded two clusters, distinctly segregating preterm or term samples (Fig. 4b). Top ranked proteins by prevalence revealed sharing across multiple individuals, and hierarchical clustering of these features clearly separated preterm versus term sera (Fig. 4c). While neither PhIP-seq or IP–MS distinguishes between maternal or fetal derived antigens, the combined PhIP-seq and IP–MS data support the notion that auto- and/or allo-reactive antibodies describe preterm pregnancy.

**Fig. 5:**
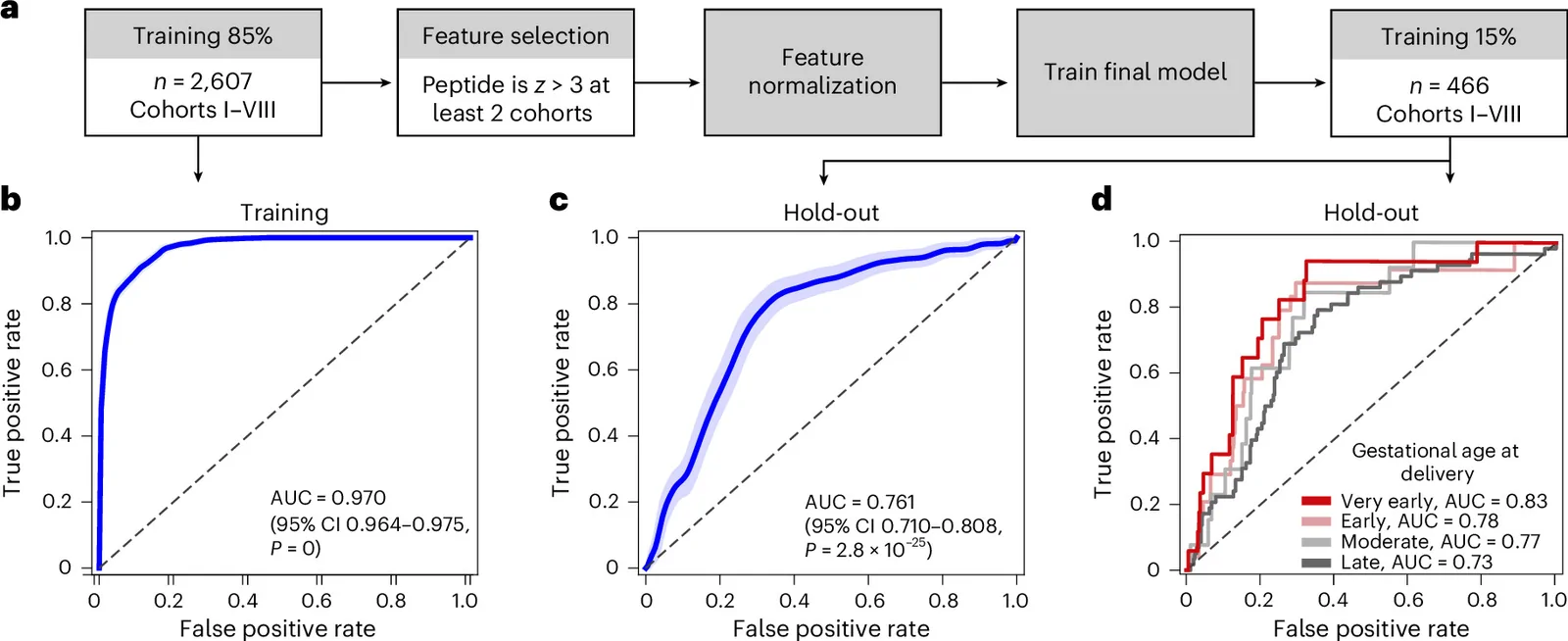
Evaluation of a predictive model of preterm birth using the serological autoreactive signature. **a**, Schematic of samples used for feature selection and final model training. **a**–**d**, ROC analysis of out-of-fold predictions from a forecasting model built on the preterm autoantibody signature in the training set (**b**), the hold-out set (**c**) and the hold-out test set stratified by gestational age group at delivery (**d**). Solid lines represent the mean ROC across 2,000 bootstrap resamples; shaded regions denote ±1 s.d.; 95% CIs are bootstrap percentile intervals (2,000 iterations). Two-sided *P* value from DeLong one-sample test versus AUC = 0.5. The dashed diagonal indicates chance-level performance (AUC = 0.5). Source data

**Extended Data Fig. 7:**
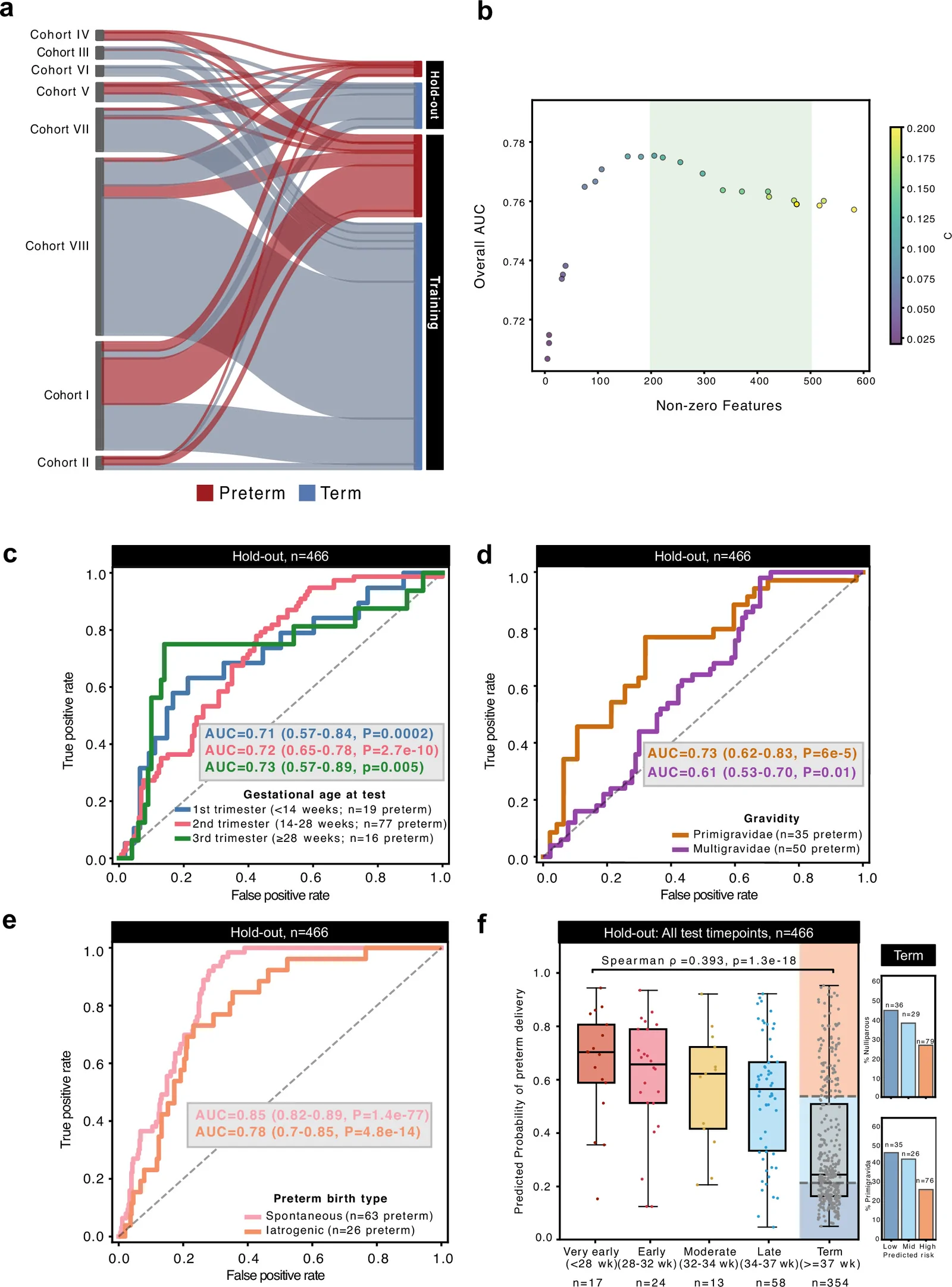
Machine learning predictor for preterm delivery. **a**. Schematic of samples designated for training or testing from each cohort with term or preterm outcomes. **b**. Scatter plot of overall AUC versus the number of non-zero features, colored by C value. Receiver operator curve analysis of model performance on the hold-out test set stratified by **c**. trimester at which the test was performed, **d**. gravidity or **e**. preterm birth type. Solid lines represent the mean ROC across 2,000 bootstrap resamples; 95% confidence intervals are bootstrap percentile intervals (2,000 iterations). Two-way p-value from DeLong one-sample test vs AUC = 0.5. **f**. Predicted probability of preterm delivery binned by preterm birth severity in the hold-out set. Shaded regions in term pregnancies correspond to tertiles of risk; boxes indicate median and interquartile range; whiskers extend to minimum and maximum values. Bar plots at right show the proportions of participants who were primigravida or nulliparous before the current pregnancy. Primigravida indicates no previous pregnancy, whereas nulliparous indicates no previous pregnancy resulting in a birth. Each dot represents an individual, Spearman correlation for significance in **f**. Source data

A simple logistic regression model was constructed to determine what predictive value may be contained within PhIP-seq profiles. Data from the eight cohorts were partitioned into sets for feature selection and model training (*n* = 2,607), with representative samples from each cohort held out for independent validation (*n* = 466; Fig. 5a, Extended Data Fig. 7a,b and Supplementary Methods). The final trained model composed of 463 peptide features was utilized to predict preterm or term delivery outcomes of the hold-out set, which was never used for training or feature selection. As expected, model performance on the validation cohorts was less than the training cohorts; however, forecasting ability was still achieved (AUC 0.76, 95% CI 0.710–0.808, *P* = 2.8 × 10^−25^; Fig. 5b,c, Supplementary Table 4 and Supplementary Methods). Discrimination was stable across trimesters of sample collection, including samples obtained during the first trimester (AUC 0.71, 95% CI 0.57–0.84, *P* = 0.002; Extended Data Fig. 7c), was highest among primigravidae (AUC 0.72, 95% CI 0.62–0.83, *P* = 5.7 × 10^−5^; Extended Data Fig. 7d), and predicted spontaneous preterm birth subtype best (AUC 0.85, 95% CI 0.82–0.89, *P* = 1.4 × 10⁻^77^; Extended Data Fig. 7e). Predicted risk scores exhibited a monotonic severity gradient, with higher scores associated with earlier gestational age at delivery (AUC 0.83, 95% CI 0.73–0.91, *P* = 2.3 × 10⁻^12^ for very early preterm versus AUC 0.73, 95% CI 0.67–0.79, *P* = 4.8 × 10⁻^12^ for late preterm; Fig. 5d), and this remained significant even after restricting to samples collected before 16 weeks of gestation (AUC 0.77, 95% CI 0.62–0.90, *P* = 0.0002 for early preterm). Notably, term samples predicted to be at highest risk of preterm birth were predominantly from multiparous women, suggesting that prior pregnancy history may influence the immunological signature (Extended Data Fig. 7f). These findings suggest that PhIP-seq captures a heterogeneous, pregnancy humoral immune state that precedes preterm birth and reflects distributed antibody reactivities rather than a single dominant specificity.

## Targets within the preterm autoantibody signature

The features that constitute the preterm autoantibody signature correspond to a rich landscape of proteins distributed across structures and functions related to pregnancy and the fetus. Identification of most prevalent features (Supplementary Table 3) associated with risk included PLA2G2A, a secreted phospholipase involved in prostaglandin biosynthesis, a process that is essential for placental adaptation and labor onset among other roles and IZUMO3, classically a sperm protein^36^, raising the possibility of alloantibody responses to paternal antigens.

**Fig. 6:**
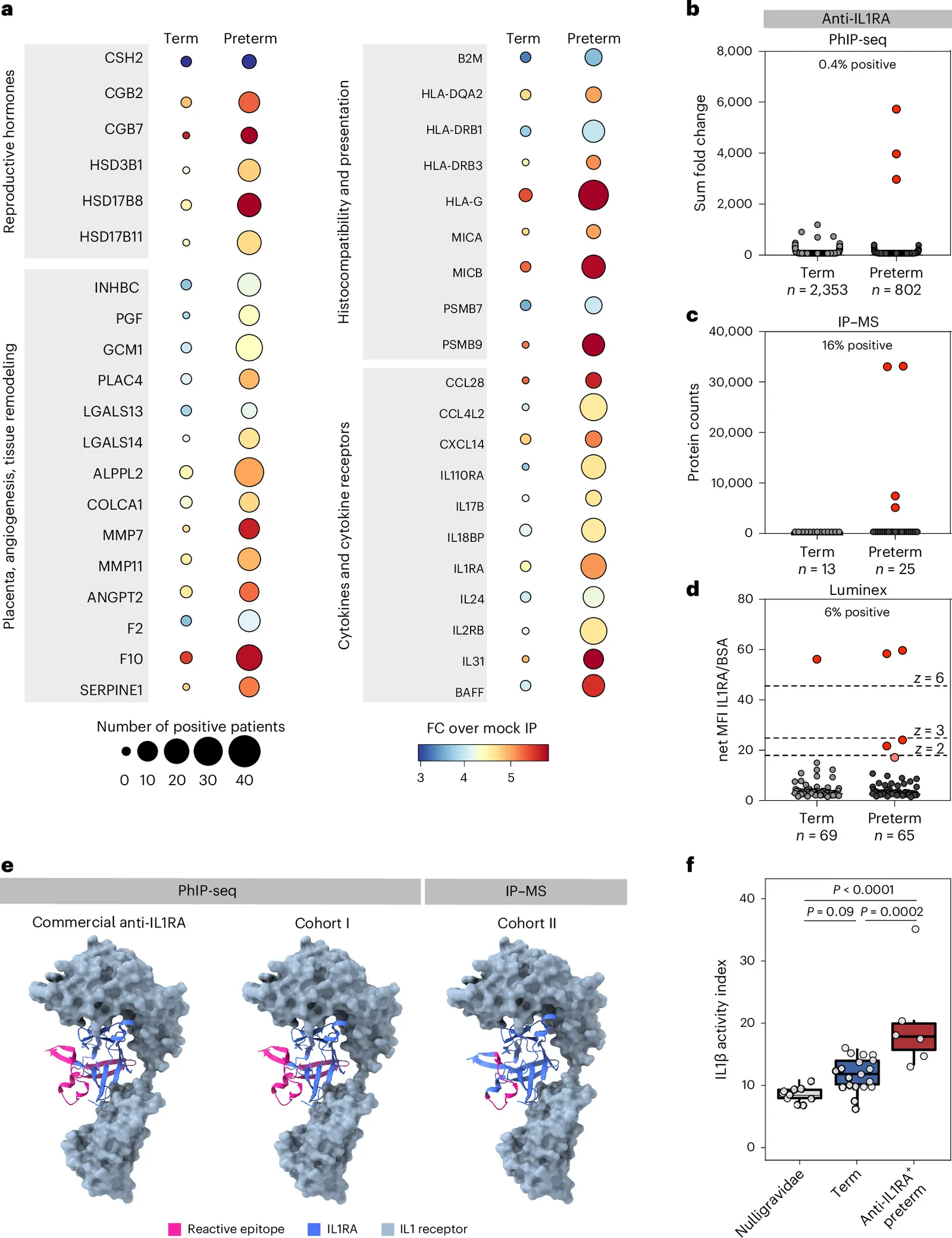
Targets within the preterm autoantibody signature. **a**, Dot plot of antibody reactivity for preterm-associated hit genes, grouped by functional category (columns), in healthy term pregnancies from cohort I (left) and preterm pregnancies from all cohorts (right). Dot color indicates reactivity expressed as the log_10_ fold change over mock IP, summed across a protein’s hit peptides and across individuals within each group; the color scale is winsorized to the 1st–95th percentile of values across the plotted genes; hues reflect rank within the panel. Dot size indicates the number of seropositive individuals. Only peptides called as significant preterm hits are shown. **b**–**d**, Anti-IL1RA antibodies in preterm versus term pregnancies identified using fold change above mean in term pregnancies detected by PhIP-seq (**b**), protein counts detected by placental IP–MS in cohort II (**c**) or net MFI of IL1RA over BSA detected by Luminex assay in cohort V (**d**). Percent positivity in preterm pregnancies is indicated and defined using assay-specific thresholds based on term pregnancies: samples exceeding 50 s.d. above the term mean for PhIP-seq, detectable IL1RA peptides by IP–MS, or 2 s.d. above the term mean for the Luminex assay. **e**, Location of reactive epitope (magenta) determined by PhIP-seq or IP–MS for an anti-IL1RA commercial antibody (R&D MAB280) or enriched in preterm sera in cohort I or cohort II on the structure of human IL1RA (green) bound to the IL1 receptor (blue). PDB accession: 1IRA. **f**, Quantification of secreted embryonic alkaline phosphatase (SEAP) produced by HEK-Blue IL-1β reporter cells incubated with human IL-1β and IL1RA in the presence of *n* = 36 female nulligravidae, term or anti-IL1RA^+^ sera. IL1β activity was calculated by dividing experimental signal with patient sera by cells treated with IL1β alone. Dots represent individual human serum samples in **b**–**d** and **f**. Statistical significance calculated by one-way ANOVA with Tukey post hoc in **f**. Boxes indicate median and interquartile range; whiskers extend to minimum and maximum values. Source data

Pathway enrichment analysis was also used to aggregate and summarize relationships between preterm autoantibody signature features (Supplementary Methods). These pathways included hormone synthesis and signaling, placental angiogenesis and tissue remodeling, histocompatibility and cytokine categories (Fig. 6a). Several critical reproductive hormones (and/or their receptors) were significantly enriched (CSH2, CGB2, CGB7 and PGF). Vascular remodeling of maternal spiral arterioles is a hallmark of successful placentation^37^. Accordingly, proteases (for example, MMP11 and MMP7) and canonical angiogenesis proteins (for example, LAMB3) were significantly targeted in preterm pregnancies (Fig. 6a).

Preterm autoantibody targets also included histocompatibility and antigen-processing proteins (B2M, HLA^−^DPB1, HLA-DQA2, MICA, MICB and PSMB9) and the placental-specific HLA-G (Fig. 6a). Anti-HLA antibodies increase the risk of spontaneous preterm delivery^27^ and are associated with complement deposition in the placental endothelium, which occurs more frequently in spontaneous preterm labor^28^. Anti-cytokine antibodies, known to increase susceptibility to infection^38^, were also identified. Of these, anti- interleukin 1 receptor antagonist (IL1RA) and anti-IL2RB were the most significantly enriched. Furthermore, anti-IL1RA was the only anti-cytokine reactivity that was detected both by PhIP-seq and IP–MS (Fig. 6a and Supplementary Tables 3 and 4). Together, the essential roles of these proteins, their expression on placenta and their enrichment by preterm sera suggest that, beyond a breakdown in tolerance, there is the possibility that some of the corresponding autoantibodies may possess pathologic function-blocking activities related to pathways that maintain successful pregnancy.

## Neutralizing anti-IL1RA in a subset of preterm pregnancies

Our comprehensive profiling of autoreactivity during pregnancy uncovered a heterogeneous immune landscape, with preterm pregnancies exhibiting patterns that distinctly diverge from those seen in term pregnancies. To better understand autoreactivities that may contribute to the pathogenesis of preterm birth, we screened for proteins with established roles in the initiation of parturition and their corresponding antagonists. We purposely included low-prevalence hits, especially those targeting secreted proteins. Among candidates, we identified IL1RA (encoded by the *IL1RN* gene) as a possible lead. IL1RA is the secreted competitive antagonist of IL1β, whose labor-promoting effects are well established^6^; the existence of autoantibodies to the secreted competitive antagonist, IL1RA, has been noted in other contexts^39–42^. Despite low prevalence by PhIP-seq across all cohorts (0.4%; Fig. 6b), we pursued studies of anti-IL1RA as an exemplar of preterm autoreactivity with a plausible mechanistic consequence.

**Extended Data Fig. 8:**
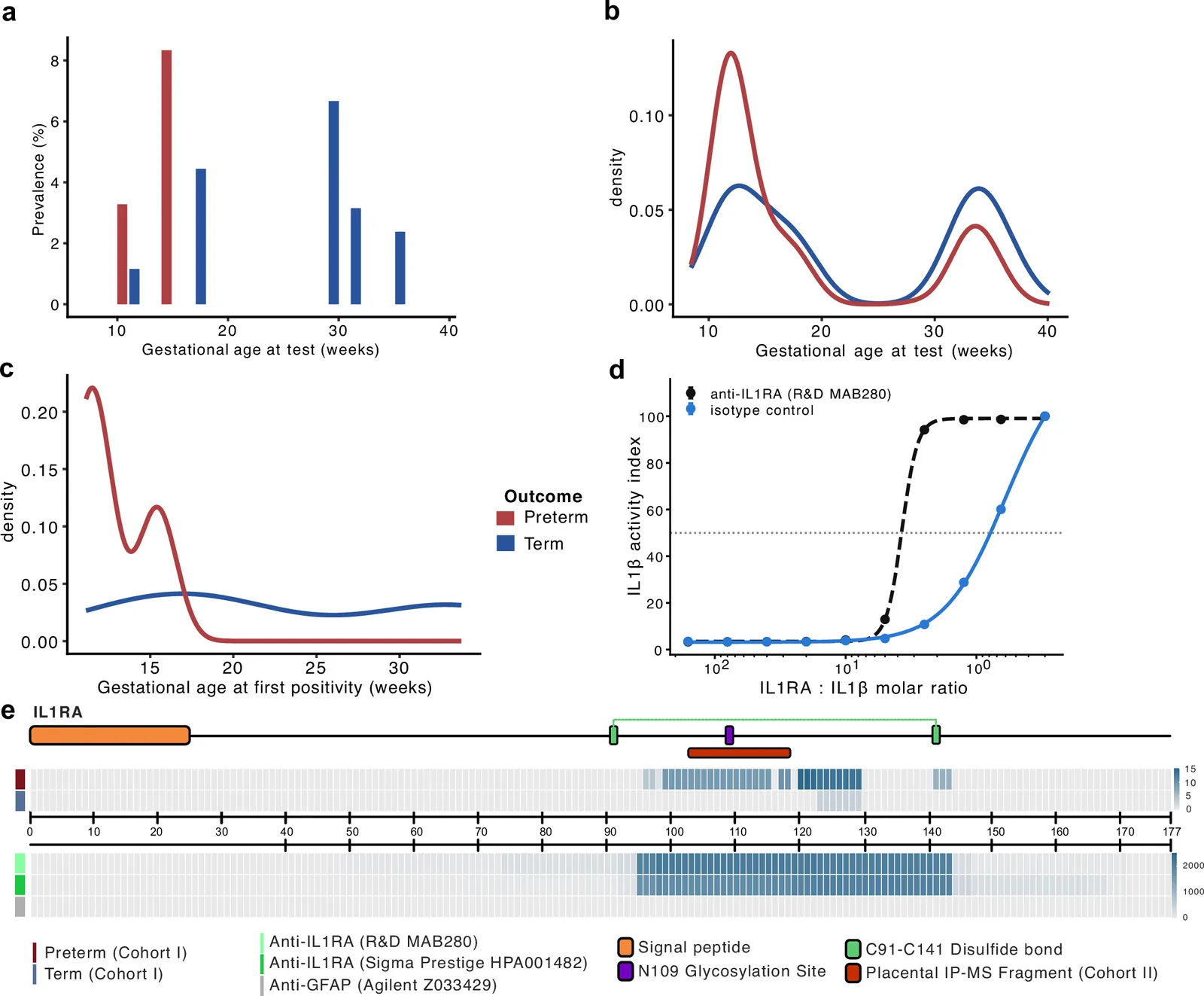
Prevalence of IL-1RA autoantibody positivity across gestational age. **a**. Prevalence of anti-IL1RA positive pregnancies binned by gestational age at sampling (2-week bins). Bars show the proportion of positive samples within each bin. Only bins with ≥2 total samples were included. Density of anti-IL1RA positive pregnancies with respect to **b**. gestational age at testing or **c**. gestational age at first detectable IL-1RA autoantibody positivity in subjects who became positive at least once. For each individual, positivity was defined as the earliest gestational age at which anti-IL1RA was detected. Pregnancies were considered to be anti-IL1RA positive if the MFI over BSA exceeded three standard deviations from the mean in term pregnancies. Preterm and term outcomes are represented in **a**-**c**. **d**. Quantification of secreted embryonic alkaline phosphatase (SEAP) produced by HEK-Blue IL-1β reporter cells incubated with human IL-1β and IL-1RA across a titration of IL-1RA (IL1RA:IL-1β molar ratio), in the presence of a purified anti-IL1RA antibody or an isotype-matched control antibody. IL-1β activity was calculated by dividing the experimental signal by that of cells treated with IL-1β alone, and is expressed as the percentage of maximal response. Dose–response curves fitted with a four-parameter logistic (4PL) model; points, mean ± s.d. of duplicate measurements; dotted line, 50% response. IC50 derived from the 4PL fits, expressed as the IL1RA:IL-1β ratio at half-maximal response. **e**. Domain structure of IL1RA with placental IP-MS fragment enriched in preterm pregnancies in Cohort II (red; top). Heatmap of mean corresponding positional enrichment for each amino acid by PhIP-seq in term and preterm pregnancies in Cohort I (middle) or in IPs with commercial anti-IL1RA or off-target anti-GFAP antibodies. Source data

To assess the prevalence of anti-IL1RA antibodies, two orthogonal approaches were used. First, IP–MS was used to interrogate cohort II sample (20–30 weeks gestation at sample collection; randomly sampled *n* = 25 preterm, *n* = 13 term). This analysis recovered antibody bound IL1RA in 16% of preterm samples (Fig. 6c and Supplementary Table 6). In an independent cohort (cohort V; 11–15 weeks gestation at sample collection; *n* = 65 preterm, *n* = 69 term), we implemented a Luminex assay, which detected anti-IL1RA in 6% of preterm pregnancies and <1.5% of term pregnancies (Fig. 6d). We further profiled a random sample of 894 longitudinal sera from cohort VIII (*n* = 86 preterm, *n* = 808 term) using the same Luminex platform. Prevalence of anti-IL1RA positivity in preterm pregnancies was 2.3% before 18 weeks (*n* = 3/129). By contrast, term pregnancies exhibited ≤0.4% prevalence before 18 weeks (*n* = 2/516; Fisher exact test OR 6.12, *P* = 0.057). We also noted anti-IL1RA positivity among term pregnancies at later gestational ages, including 2.5% after 18 weeks (*n* = 4/90; Extended Data Fig. 8a–c and Supplementary Table 6). Together, these results support a model in which early emergence, rather than presence alone, is associated with increased preterm risk.

To identify the specific epitope on IL1RA that is targeted by anti-IL1RA antibodies, peptide fragments from PhIP-seq and IP–MS were analyzed. Positional mean enrichment analysis of PhIP-seq data (Supplementary Methods) revealed that the majority of anti-IL1RA^+^ pregnancies targeted overlapping peptides between D99 and D129 amino acid residues on IL1RA. This epitope (Fig. 6e and Extended Data Fig. 8d), opposite the receptor-engaging face, is highly charged and contains the only N-linked glycosylation residue in the protein (N109)^43^. Antibodies to this epitope have previously been reported in IgG4 disease^40^, and it is also targeted by two commercial antibodies. IP–MS fragment analysis identified a single 16-amino-acid peptide (103-LQLEAVNITDLSENRK-118) that was fully contained within the target PhIP-seq fragment (Fig. 6e and Extended Data Fig. 8d). These data confirm the target epitope enriched by both PhIP-seq and IP–MS are localized to the same region, and furthermore, these data suggest that anti-IL1RA antibodies present in multiple preterm sera converge on the same region of the protein.

To investigate the functional consequences of anti-IL1RA antibodies, a previously described IL1β reporter cell line and validated assay was utilized^39^. Reporter cells were stimulated with recombinant IL1β and a titration of increasing amounts of excess of IL1RA and serum or antibody (Methods). Cells treated with a commercial anti-IL1RA antibody (R&D AF-280-NA) that primarily targets the patient epitope resulted in significant neutralization of IL1RA as measured by greater IL1β activity, shifting the IL1RA:IL1β half-maximal inhibitory concentration six-fold relative to an isotype control (IC_50_ 3.71 versus 0.61; Extended Data Fig. 8d). Similarly, anti-IL1RA^+^ preterm sera also resulted in significantly higher IL1β activity and neutralized IL1RA (Fig. 6f). Conversely, cells treated with sera from nulligravidae or term women exhibited low IL1β activity in the presence of IL1RA, with IL1β activity increasing monotonically across nulligravid, term, and anti-IL1RA^+^ preterm groups (Kruskal–Wallis *P* = 6.7 × 10⁻^5^; ordered-trend Spearman *ρ* = 0.62, *P* < 1 × 10⁻^5^). Thus, both commercial antibodies and preterm patient sera are sufficient to neutralize IL1RA, thereby licensing IL1β signaling.

## Anti-IL1RA promotes fetal malperfusion in vivo

**Fig. 7:**
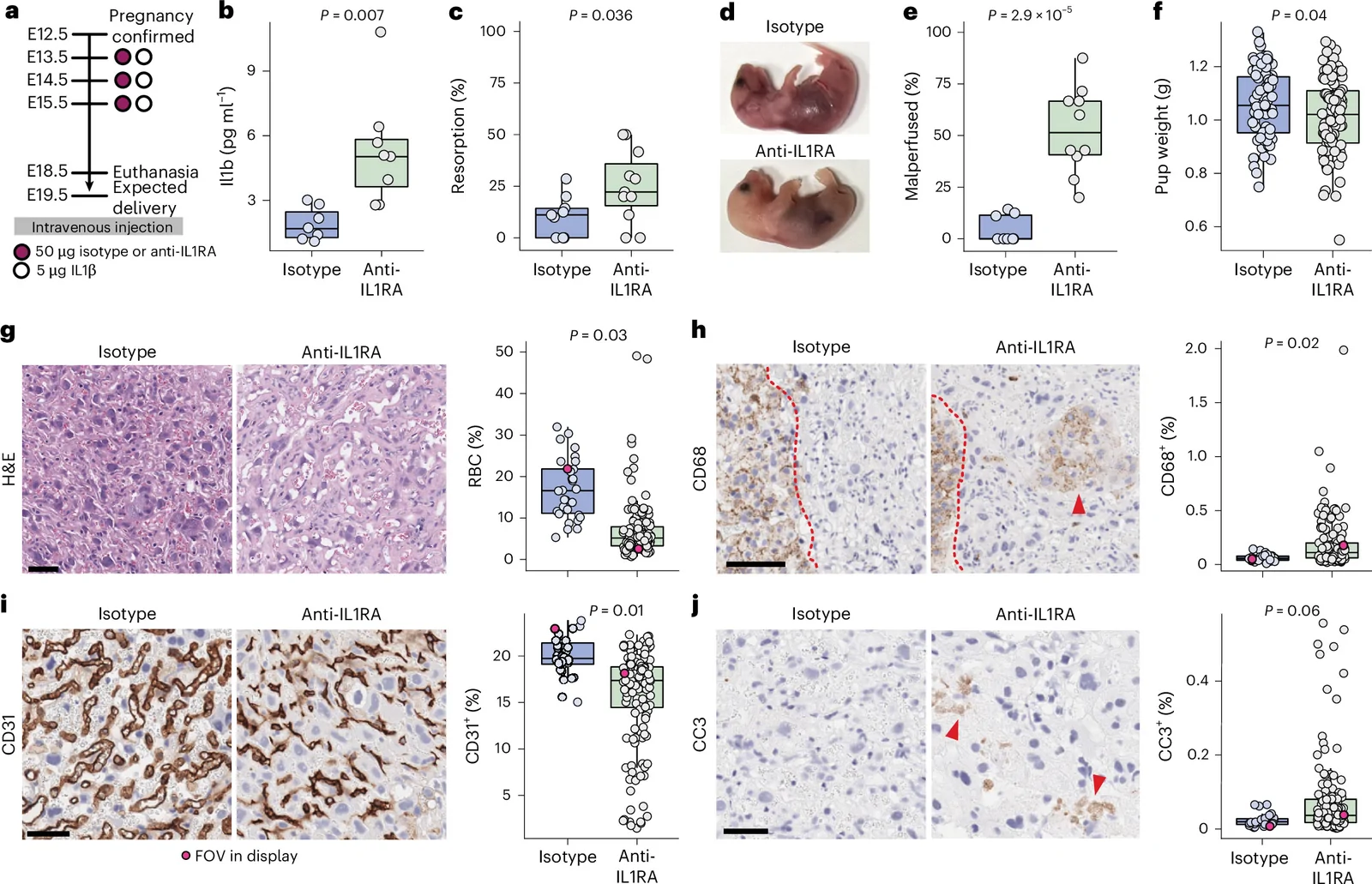
Anti-IL1RA deficiency induces reproductive defects in vivo. **a**, Schematic of injections in timed-pregnant C57Bl/6 mice. **b**, Quantification of Il1b (pg ml^−1^) in *n* = 15 mouse maternal serum by ELISA. **c**, Complete resorption rate (%) in *n* = 20 mice. **d**, Representative images of nonresorbed pups. **e**, Percentage of malperfused pups among live pups in *n* = 18. **f**, Pup weight (in grams) of 106 pups from dams treated with either an isotype control antibody or anti-IL1RA at E18.5. **g**–**i**, Micrographs of representative placentas stained with H&E (**g**), anti-CD68 (**h**), anti-CD31 (**i**) and anti-cleaved caspase 3 (CC3) (**j**) from isotype or anti-IL1RA injected dams at E18.5 supplemented with 5 µg of IL1β. Quantification of red blood cell (RBC, **g**) or antibody positive (**h**–**j**) area in each field of view (FOV) is shown the in right panel; the magenta dot denotes the field of view in display. Dots represent individual mice in **b**,**c** and **e**,**f** from at least two independent experiments; and individual FOV in **g**–**j** from at least *n* = 3 mice per group. All samples in **b** were run in technical duplicate. Statistical significance calculated by two-sided Kolmogorov–Smirnov pairwise test in **b**,**c** and **e**,**f**. FOV quantifications were averaged, and the per-mouse two-sided Kolmogorov–Smirnov pairwise test is reported in **g**–**j**. Boxes indicate the median and interquartile range; whiskers extend to minimum and maximum values. Source data

**Extended Data Fig. 9:**
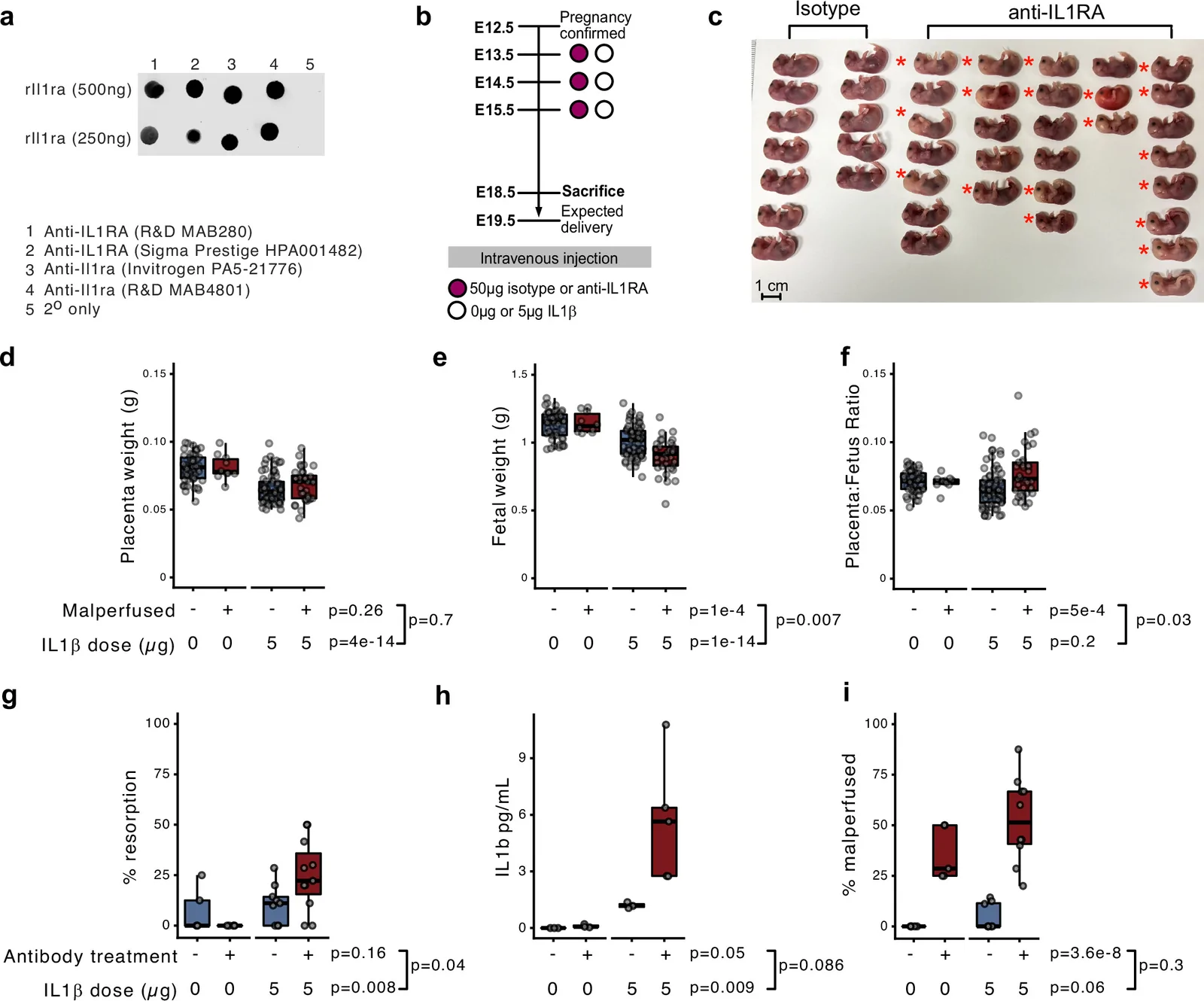
Neutralizing anti-IL1RA antibodies dysregulate IL1β *in vivo*. **a**. Dot blot of recombinant mouse 500 ng (top) or 250 ng (bottom) Il1ra probed with anti-human IL1RA antibodies (lanes 1-2), or anti-mouse Il1ra (lanes 3-4), or secondary only control (lane 5). **b**. Schematic of injections in timed-pregnant C57Bl/6 mice. Isotype or anti-IL1RA commercial antibody was injected daily on embryonic days (E) 13.5-15.5 with either 0 or 5 µg of IL1β; mice were sacrificed on E18.5. **c**. Representative images of fetuses from isotype or anti-IL1RA injected mice. Each column represents pups from one dam, asterisk indicated observation of gross vascular defects. Quantification of **d**. placenta weights, **e**. fetal weights, and **f**. placenta:fetus weight ratio in malperfused or normal pups relative to IL1β dose from n = 169 fetoplacental units. Quantification of **g**. the percent resorption in n = 30 mice, **h**. maternal serum Il1b levels (pg/mL) in n = 21 mice and **i**. percent malperfusion in n = 28 mice treated with anti-IL1RA (+) or isotype (-) and relative to IL1β dose. Two-way ANOVA test for significance and interaction in **d**-**I**; boxes indicate median and interquartile range; whiskers extend to minimum and maximum values. Source data

To further explore the physiological consequence of circulating anti-IL1RA antibodies in vivo, the role of maternal anti-IL1RA antibodies was investigated during mouse pregnancy, in which exposure to IL1β results in reproductive defects^17^. We hypothesized that, because IL1RA regulates IL1β, adoptive transfer of anti-IL1RA antibodies would inhibit the ability of IL1RA to rescue pregnancies threatened by IL1β-induced inflammation, resulting in worse pregnancy outcomes. First, we confirmed that the patient epitope targeting commercial anti-IL1RA antibody was cross-reactive to the native mouse orthologue by dot blot (Extended Data Fig. 9a). Timed-pregnant dams were injected intravenously three times (embryonic days (E)13.5–15.5) with either the anti-IL1RA antibody or an equivalent amount of isotype control in the context of IL1β-induced inflammation (Fig. 7a). Levels of mouse Il1β were measured in maternal serum by enzyme-linked immunosorbent assay (ELISA) on the day before expected delivery (E18.5). Concentration of maternal Il1β was significantly greater in anti-IL1RA-injected dams as compared with isotype controls (Fig. 7b), indicating that anti-IL1RA neutralizes the ability of the antagonist to regulate Il1β during pregnancy. Consistent with our hypothesis, anti-IL1RA injected dams also exhibited profound reproductive defects. Resorption rate was significantly elevated (Fig. 7c). Among the nonresorbed pups, a significantly greater proportion of fetuses from anti-IL1RA pregnancies exhibited less prominent cephalic vasculature and pallid skin color (Fig. 7d-e and Extended Data Fig. 9c), suggestive of a defect in placental perfusion. All nonresorbed pups from anti-IL1RA pregnancies weighed significantly less than isotype-injected controls (Fig. 7f). Malperfused pups weighed significantly less than normal pups, although a defect in gross placental weight was not observed (Extended Data Fig. 9d–f). Histological analysis of placenta revealed reduced red blood cell perfusion into the placental labyrinth in animals treated with anti-IL1RA (Fig. 7g). Placentas from anti-IL1RA-treated dams also exhibited vessel constriction (lower CD31^+^ area), immune infiltration (greater number of CD68^+^ cells) and elevated apoptosis (cells with cleaved caspase 3), as determined by immunohistochemistry (IHC; Fig. 7h–j).

Anti-IL1RA impaired IL1β signaling and produced pregnancy pathology even without exogenous IL1β, but both factors contributed to resorption phenotypes. IL1β supplementation had a significant effect (*P* = 0.008), and its interaction with anti-IL1RA was also significant (*P* = 0.04; Extended Data Fig. 9g). Maternal IL1β levels were driven by both IL1β dose and antibody administration (Extended Data Fig. 9h). By contrast, fetal malperfusion was highly specific to anti-IL1RA, as the IL1β dose, alone or in interaction, did not significantly explain variance (Extended Data Fig. 9i). These patterns extended to fetal and placental weights: placental weights were reduced by IL1β, whereas fetal growth restriction tracked with malperfusion (Extended Data Fig. 9d–f). Together, these results show that anti-IL1RA is sufficient to disrupt IL-1β regulation and induce vascular and fetal defects, but pathology is most severe when IL1β is concurrently dysregulated.

## IL1RA is expressed in placental vessels

**Fig. 8:**
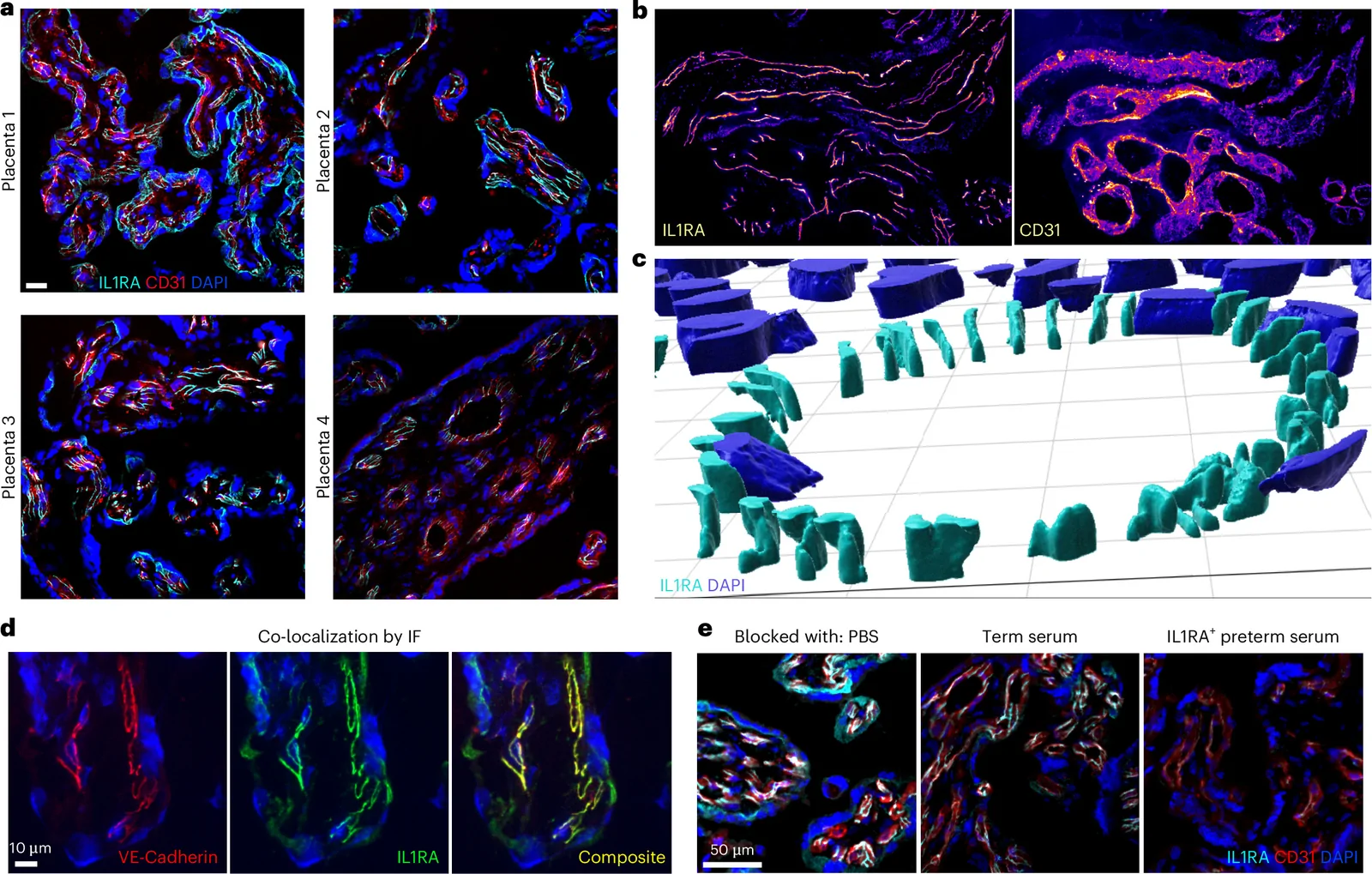
IL1RA localizes to endothelial adherens junctions in human placenta. **a**, Spinning disk confocal images of indirect immunofluorescence (IF) of IL1RA (cyan) and CD31 (red) in *n* = 4 term human placentas. Composite images with 4′,6-diamidino-2-phenylindole (DAPI) counterstain. Scale bar, 10 µm. **b**, Single channel emission of placenta stained with anti-IL1RA (left) or CD31 (right). Representative image of at least ten independent experiments. **c**, Space-filling model of a three-dimensional reconstruction of a placenta vessel immunofluorescence probed with anti-IL1RA (cyan) and counterstained with DAPI (blue). **d**, Costaining with anti-VE-cadherin (left) or anti-IL1RA antibody (middle). Composite (right) indicates overlay of signal from anti-VE-cadherin and anti-IL1RA. Representative image of three independent experiments. **e**, Indirect immunofluorescence of placenta preblocked with PBS, term sera or anti-IL1RA^+^ preterm sera from cohort II. Overlay of signal from anti-CD31 (red), anti-IL1RA (cyan) and DAPI (blue). Representative images from three experiments with *n* = 12 patient sera. DAPI counterstain in blue. Scale bars, 10 µm (**d**) and 50 µm (**e**). Source data

We hypothesized that IL1RA may also be expressed on the placental surface, where it could serve as a target for maternal alloantibodies. To investigate localization of IL1RA in human placenta, we used indirect immunofluorescence with a commercial antibody to IL1RA (Sigma Prestige HPA001482). A striking pattern of IL1RA localization was found near CD31-positive fetal vessels within the placenta (Fig. 8a–c) that also co-localized with VE-cadherin in endothelial cell junctions^44^ (Fig. 8d). While IL1RA is known to be secreted, these results suggest a substantial amount of this tolerogenic protein is localized to the endothelial adherens junctions in the placenta.

**Extended Data Fig. 10:**
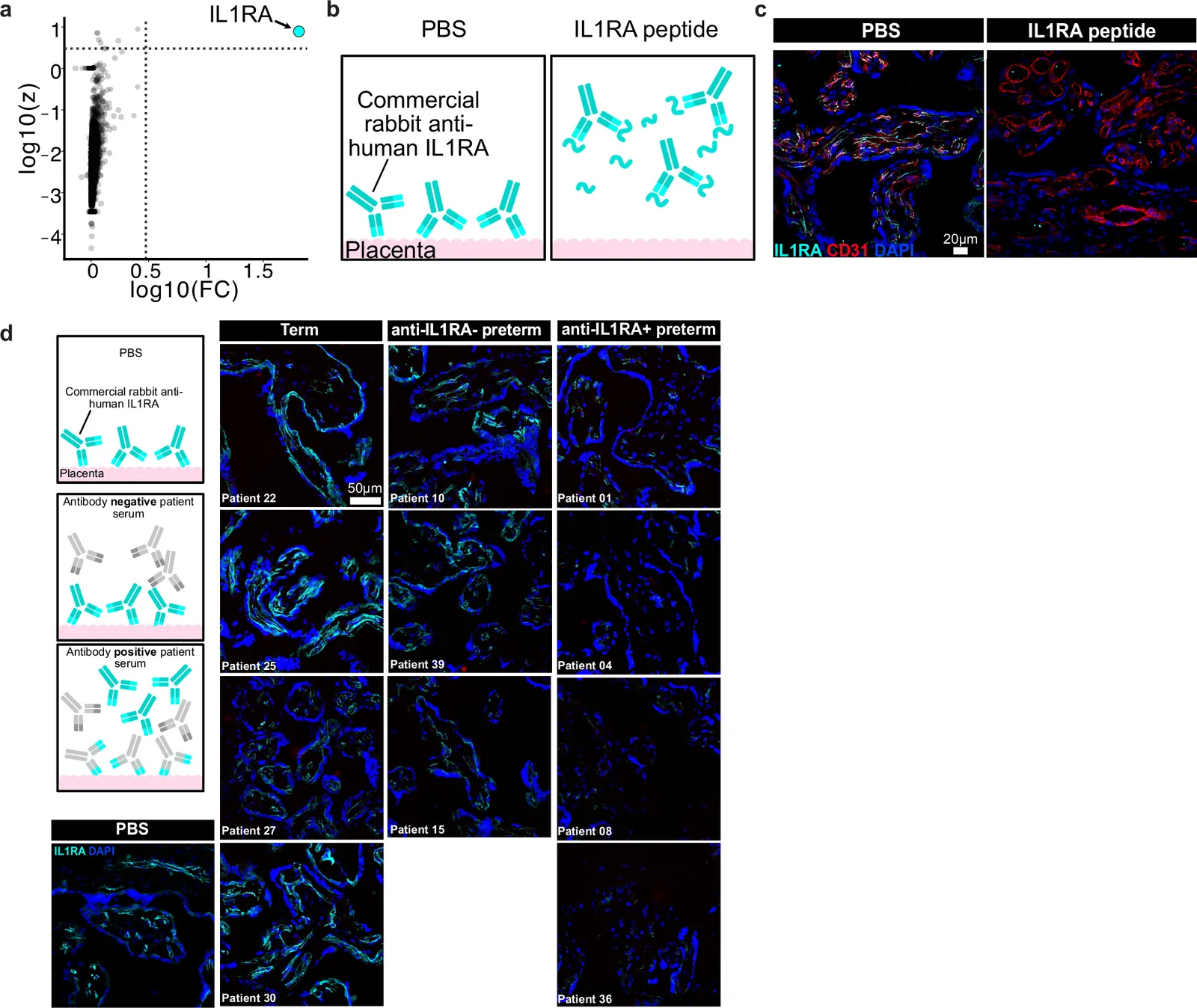
IL1RA localizes to placental vessels. **a**. Volcano plot of PhIP-seq enrichments utilizing commercial anti-IL1RA antibody represented by log10(z-score above mock IP) and log10(fold change over mock IP). Each dot represents one protein in the PhIP-seq library. **b**. Schematic of peptide blocking experiment. **c**. Indirect immunofluorescence of IL1RA and CD31 in experiments with (right) and without (left) a blocking peptide. Representative images of three experiments. **d**. (inset) Schematic of blocking experiment with patient sera and counterstaining with commercial anti-IL1RA antibody. Indirect immunofluorescence of IL1RA (cyan) in placental sections pre-blocked with anti-IL1RA negative term serum, anti-IL1RA positive preterm serum, or PBS. Each slide was blocked with serum from a different subject. Scale bar represents 50 µm, DAPI counterstain. Representative images of five experiments. Source data

To address whether the observed pattern of IL1RA co-localization was authentic, or due to cross reactivity of the antibody with other targets (such as VE-cadherin), PhIP-seq was performed with the same antibody and analyzed for enrichment. Only the IL1RA derived peptides were enriched above background, suggesting that the commercial IL1RA antibody is highly specific (Extended Data Fig. 10a). As a further validation of antibody specificity, a blocking assay was performed with the peptide immunogen used to generate the antibody (Extended Data Fig. 10b). The signal corresponding to IL1RA was eliminated, again suggesting that this antibody is highly specific (Extended Data Fig. 10c). Together, these data support the notion that IL1RA is bound at placental endothelial cell junctions, in close proximity to other junction proteins, such as VE-cadherin.

To determine whether anti-IL1RA antibodies functionally bind placenta, we investigated whether anti-IL1RA^+^ patient sera were capable of blocking IL1RA on placental tissue from binding by a commercial antibody. A positive result would further confirm the existence and specificity of anti-IL1RA antibodies with respect to native placental tissue-bound IL1RA protein. Placenta was first blocked with either phosphate-buffered saline (PBS) or term or anti-IL1RA^+^ patient sera, and then indirect immunofluorescence was performed with the commercial anti-IL1RA antibody (Methods; Extended Data Fig. 10d). The characteristic pattern of IL1RA localization to fetal vessel cell–cell junctions was clearly observed in the absence of sera, term sera or anti-IL1RA^−^ preterm sera (Fig. 8e and Extended Data Fig. 10d). However, the IL1RA localization signal was blocked in placental tissue treated with anti-IL1RA^+^ preterm sera (Fig. 8e and Extended Data Fig. 10d). These data strongly support the presence of highly specific anti-IL1RA^+^ autoantibodies in the maternal sera of a subset of preterm pregnancies, capable of recognizing native tissue-bound IL1RA in normal placenta.

## Discussion

Certain pathogenic alloantibodies are known to drive pregnancy complications in humans^29,30^, motivating a systematic examination of maternal autoreactivity during gestation. By profiling more than 2,000 maternal sera across eight pregnancy cohorts, we defined a comprehensive landscape of humoral autoreactivity in term and preterm pregnancies. Overall autoreactivity increased with advancing gestational age and gravidity, underscoring the dynamic and evolving nature of the maternal immune repertoire. We identified a preterm birth-associated autoantibody signature that was consistently enriched across geographically and ethnically diverse cohorts. Notably, these reactivities were detected an average of 12 weeks before preterm delivery, indicating that serologic changes may precede clinical onset rather than result from it. Because preterm birth arises from diverse pathophysiological causes, including inflammatory, vascular and idiopathic etiologies, the associated autoantibody landscape is correspondingly heterogeneous, probably contributing to the large feature set required for classification. The autoreactivies that comprise the signature may be mechanistically pathogenic and/or reflect broader immune dysregulation.

One limitation of this work is that the signature does not reveal the underlying triggers or events that lead to defects in self-tolerance associated with preterm birth. However, within this broader signature, multiple proteins comprise plausible autoreactive targets of functional significance. Among these, a subset of preterm pregnancies harbored functionally inhibitory antibodies against the IL1RA that exacerbated IL-1β-driven reproductive defects in mice and were capable of binding IL1RA in human placenta. The prevalence of anti-IL1RA antibodies varied across orthogonal detection methods (PhIP-seq, IP–MS, Luminex, cell-based reporter assay and immunofluorescence blocking), ranging from 0.4% by PhIP-seq to 16% by IP–MS. Such assay-dependent variation in prevalence has been reported for anti-cytokine antibodies^45^ and probably reflects differences in sensitivity, epitope accessibility and the distinction between linear and conformational epitope detection. Furthermore, sample selection for orthogonal validation was determined by sample availability and the number of anti-IL1RA-positive pregnancies identified across cohorts remains underpowered to assess associations with clinical metadata variables. Notably, anti-IL1RA antibodies were also detected in a small fraction of term pregnancies, although these typically appeared later in gestation and did not exhibit neutralizing activity in vitro, suggesting that timing of emergence and functional capacity, rather than presence alone, distinguish pathogenic from benign anti-IL1RA responses.

Rare genetic deficiencies in IL1RA (DIRA) result in a greater rate of preterm birth and intrauterine growth restriction^46^, whereas *Il1rn*-knockout mice exhibit reduced litter sizes, growth restriction^47^ and vasculitis^48^, mirroring our findings of antibody-induced IL1RA deficiency. The emergence of anti-IL1RA antibodies may reflect post-infectious or autoimmune immune priming, as anti-IL1RA antibodies have been described after coronavirus disease infection^41^, and in IgG4^40^ and Still’s disease^42^. Together, these findings support a two-hit model, in which preexisting or infection-induced dysregulation of IL-1β signaling is amplified by anti-IL1RA antibodies, tipping the balance toward inflammation, placental injury and pregnancy loss. Although the pathogenic role of these antibodies requires further investigation, recombinant IL1RA (anakinra; SOBI) has been used clinically for over two decades with established long-term safety in nonpregnant populations^49^ and limited case reports of use in pregnancy^50^. These data suggest that anakinra use in pregnancy to prevent preterm labor merits further evaluation, particularly in stratified cohorts defined by anti-IL1RA antibody status.

Beyond an individual protein, these collective proteome-wide data, representing thousands of individuals, reveals that pregnancy itself elicits a broad and shared program of humoral autoreactivity, and that preterm birth is distinguished by a markedly expanded signature targeting proteins essential for placental function, vascular remodeling and immune regulation. The enrichment of autoreactivity with gravidity further suggests that repeated pregnancy may shape the maternal humoral response to fetal, placental or pregnancy-associated antigens. Supporting this, prior pregnancy may not simply represent a clinical risk factor, but an immunological exposure history that alters the baseline state from which subsequent pregnancies begin. The breadth of this landscape suggests that distinct autoantibody-defined subtypes of preterm birth exist, each potentially amenable to serologic identification and targeted intervention. In women with recurrent preterm birth and evidence of pathogenic humoral autoreactivity, these findings may ultimately support evaluation of selective immunomodulatory or immunosuppressive strategies, although such approaches will require careful mechanistic and clinical validation. Together, these advances may yield improved management of pregnancies at risk for preterm delivery and reductions in neonatal morbidity and mortality.

More broadly, this study identifies a proteome-wide humoral immune program elicited by pregnancy, as well as distinct, shared changes preceding preterm delivery. The reprogramming of autoreactivity across gestation, the cumulative effect of gravidity, the enrichment of histocompatibility and antigen-processing targets, and the existence of antibodies against cytokine regulators together suggest that maternal B cell responses are dynamically shaped by fetal, placental and inflammatory antigen exposure. Notably, antibodies targeting HLA proteins may reflect maternal alloimmune responses to paternally inherited fetal HLA antigens. Fetal HLA genotypes were not available for these cohorts, precluding direct assessment of whether maternal HLA antibodies matched fetal alleles; paired maternal antibody and fetal HLA profiling will be important in future studies to establish the specificity and potential functional relevance of these responses. In this context, preterm birth-associated antibodies may represent not only biomarkers of disease risk, but also molecular evidence of disrupted maternal–fetal immune tolerance. Some reactivities may reflect failed tolerance to pregnancy-associated antigens, others may represent alloimmune responses to fetal or placental variants, and still others may arise secondary to tissue injury or infection. The convergence of these signals on pathways involved in antigen presentation, vascular integrity, placental remodeling and IL-1β regulation suggests that humoral immunity may participate in multiple mechanistic routes to early parturition.

## Methods

### Human cohorts and samples

Sera from cohort I were obtained from a California-wide biobank (Committee for the Protection of Human Subjects within the Health and Human Services Agency of the State of California protocol number 12-09-0702). Sera from cohort II, cohort III, cohort VI and cohort VII (University of California San Francisco (UCSF) IRB numbers 10-00505, 20-31171, 20-32077, 20-32779 and 16-20474; San Francisco, CA) were obtained from deliveries at UCSF. Sera from cohort IV (UCSF IRB number 10-00350) were obtained from UCL Hospital and Homerton University Hospital of the National Health Services of the United Kingdom. Sera from cohort V were obtained from ‘Fondazione IRCCS Policlinico San Matteo’ (San Matteo Research Hospital) in Pavia, Italy, under RC08061819 and RC08061821 approved IRB protocols. Sera from cohort VIII (IRB numbers 2009P000557 and 2014P001109, Boston, MA) were obtained from Brigham and Women’s Hospital in Boston, MA, USA, from the previously described VDAART clinical trial^51^. Deidentified healthy control (nonpregnant) plasma was collected from two sources: courtesy of New York Blood Center (New York, NY) and donors from a UCSF community drive (UCSF IRB number 22-3611; San Francisco, CA). All samples were collected under the referenced institutional review board protocols, and all patients gave informed consent before sample collection. In some cases, patients received nominal compensation for travel to donation sites.

Uncomplicated, term pregnancies were selected from cohort I to define a baseline of autoreactivity. An uncomplicated, term pregnancy for this cohort was defined as one resulting in delivery at greater than or equal to 37 weeks of gestation and did not have a diagnosis of any of the following: placental abruption, placenta previa, chorioamnionitis, oligohydraminos, polyhydraminos, premature rupture of membranes, bacterial vaginosis, urinary tract infection, diabetes or gestational diabetes, preexisting or gestational hypertension, preeclampsia, infection during pregnancy, any specified placental condition, retained placenta with or without hemorrhage, unspecified hemorrhage, abnormal clotting or threatened abortion. Any pregnancy that required transfusion, rhesus isoimmunization or cerclage was excluded from this group. We also excluded mothers with any hypertension disorder, endometriosis, Group B streptococcus positivity, any coagulation deficiency, malignancy, asthma, allergic dermatitis, anaphylaxis, rheumatoid arthritis, systemic lupus erythematosus, autoimmune thyroiditis or mental disorder. Mothers who reported any smoking, drug or alcohol abuse or dependency during pregnancy were excluded. Pregnancies where the infant exhibited convulsions, abnormal neural imaging or examination, retinopathy of prematurity, respiratory distress syndrome, intraventricular hemorrhage, necrotizing enterocolitis, bronchopulmonary dysplasia, periventricular leukomalacia, hypoxic-ischemic encephalopathy or known congenital heart disease were also excluded from this group. Finally, we also required that women defined as having uncomplicated pregnancies did not have a history of recurrent pregnancy loss, preterm delivery or poor obstetric/reproductive outcomes.

Term pregnancies for all cohorts were defined as deliveries occurring at 37 or more weeks of gestation.

Preterm pregnancies were defined as those delivering before 37 weeks of gestation and were further subdivided into spontaneous or iatrogenic preterm delivery. Spontaneous preterm pregnancies were defined as pregnancies with spontaneous labor with delivery before 37 weeks of gestation. Iatrogenic preterm pregnancies were defined as pregnancies with delivery before 37 weeks of gestation and without spontaneous labor, which included both induced vaginal and cesarean deliveries. Indication for iatrogenic preterm deliveries was variably documented across the eight cohorts and typically included preeclampsia with severe features or concern for fetal/maternal well-being.

### Animal husbandry

All mice were housed, bred and maintained in a pathogen-free facility at UCSF. All procedures were performed in concordance with UCSF Institutional Animal Care and Use Committee (IACUC) regulations and approved protocol. Timed-pregnant C57BL/6 females mated with C57BL/6 males were purchased from Jackson Laboratories (strain number 000664).

### Mouse injections and collection

Retro-orbital injections were done following with UCSF IACUC procedural guidelines. In brief, pregnant dams were anesthetized at E13.5–E15.5 with isoflurane and injected in the retro-orbital sinus using 0.5-ml insulin syringes, with a maximum volume of 150 µl containing 0 or 5 µg of human IL1β (Peprotech 200-01B) and 50 µg of polyclonal goat IgG (isotype control; R&D AB-108-C) or of goat anti-human IL1RA antibody (R&D AF-280-NA) in sterile PBS. Reagents were mixed immediately before injection. Mice were euthanized at E18.5 with CO_2_, and a sample of serum was obtained via transcardial puncture. Resorbed and malpefrused fetuses were quantified. Whole fetuses and their placentas were collected and weighed. Mouse Il1b concentrations were measured in maternal serum by a commercial ELISA (Abcam catalogue number ab197742).

### Histological analysis of mouse placenta

Whole placentas were fixed in 4% paraformaldehyde and embedded in paraffin using standard protocols. Embedding, grossing, sectioning, IHC with anti-human antibodies to anti-CD68, anti-cleaved Caspase 3 or anti-CD31, and scanning were performed at HistoWiz. Blinded hematoxylin and eosin (H&E) slides were assessed by a licensed pathologist. IHC was quantified by defining five non-overlapping regions in each of the two placental sections for a total of ten regions for each placenta in QuPath software. Regions were randomly drawn and covered most of the placental area, avoiding anomalous regions (for example, small tears in tissue). For anti-CD68 IHC, regions of interest were drawn in the labyrinth region of the placenta. A pixel thresholding classifier was built for each of the antibody stains using a Laplacian of Gaussian prefilter setting. DAB-positive area was calculated as a percentage of total area within the defined region. Each individual region of interest was reported for isotype or anti-IL1RA-treated conditions.

### PhIP-seq with human peptidome library

The human T7 phage display library used for IP and sequencing is previously described and sera from the above cohorts was used in high-thruput protocols as previously described. Detailed protocols are available via protocols.io at https://doi.org/10.17504/protocols.io.4r3l229qxl1y/v1.

### Trapped ion mobility time-of-flight MS of immunoprecipitated placental protein lysate

Term placenta from a single donor was collected within 2 h of delivery. Biopsies were taken perpendicular to the basal surface to ensure sampling of anchoring villi, and may include a small amount of decidua; 1-cm^3^ blocks were flash frozen in liquid nitrogen. Placenta protein lysate was prepared by mechanically dissociating tissue using a glass Dounce homogenizer and an electric tissue homogenizer in RIPA buffer with protease inhibitors (Roche) on ice. Protein was quantified using Bradford Assay (Pierce), normalized to 500 µg in TNP40 and incubated with 1 µl of human sera overnight at 4 °C with overhead mixing. Proteins were subsequently incubated with a 1:1 mix of protein A and protein G Dynabeads (Thermo) for 1 h and washed five times with RIPA and once with Tris–HCl pH 7.4. A NanoDrop reading was taken under Tris–HCl pH 7.4 to quantify protein.

Supernatant was removed, and proteins were then resuspended in 8 M urea, 50 mM Tris (pH 8) before being subject to a standard on-bead protein digestion. The protein digestion procedure included disulfide reduction with 5 mM dithiothreitol, alkylation with 14 mM iodoacetamide, and an overnight digestion with Lys/C at a protein to protease ratio of 1:50. To obtain an optimal tryptic digestion, the urea concentration was then diluted to 2 M with 50 mM Tris (pH 8), followed by the addition of trypsin to a protein to protease ratio of 1:50, and a 4-h incubation at 25 °C. The peptide supernatant pH was then decreased to 2 with 7 µl formic acid and separated from the beads with a magnet. An extraction was performed on the beads by resuspension with 0.1% formic acid, the supernatant of which was added to the final sample. Peptide desalting was performed with the AssayMAP Bravo (Agilent Technologies) using standard RPS peptide cleanup cartridges and protocol^52^. Approximately 25 ng of desalted peptides were analyzed on a TIMSTOF SCP mass spectrometer (Bruker Corporation) coupled with an EASY-nLC 1200 LC system (Thermo Fisher Scientific). Peptides were separated by reverse-phase chromatography on a 25-cm column (75 μm inner diameter, packed with 1.6-μm C18 resin, AUR2-25075C18A-CSI; IonOpticks). Peptides were introduced into the mass spectrometer using a gradient starting with 2% to 8% buffer B (0.1% (v/v) formic acid in 80% acetonitrile) for 1 min, followed by an increase to 25% buffer B for 25 min then an increase to 40% buffer B for 5 min at a flow rate of 100 nl min^−1^ and were ionized by CSI (captive spray ionization).

Samples were first analyzed by PASEF-dda with a duty cycle of 1.03 s comprising one MS1 survey for every five PASEF MS2 ramps and a mass range between 100 *m*/*z* and 1,700 *m*/*z*. The trapped ion mobility spectrometry (TIMS) device ramp/accumulation time was set to 166 ms with a 100% duty cycle and a mobility range of 0.7–1.3 1/*K*_0_. Precursor ions from the survey scan were selected using an adjusted isolation width of 2 *m*/*z* below 700 *m*/*z* and 3 *m*/*z* above 800 *m*/*z* precursor mass-to-charge value in the quadrupole that aligns with the anticipated TIMS elution time relative to the ion mobility value. Fragmentation was performed by collision-induced dissociation with collisional energy interpolation between 42 eV at 0.65 1/*K*_0_, 31.92 eV at 0.8 1/*K*_0_, 36.96 eV at 1 1/*K*_0_, 42 eV at 1.2 1/*K*_0_, 47.04 eV at 1.4 1/*K*_0_ and 51.24 eV at 1.6 1/*K*_0_. Active exclusion of precursors was released after 0.2 min. Precursor repetitions were set to a target intensity of 20,000 and a threshold of 500.

Real-time searching was performed with PaSER (Parallel Search Engine in Real-Time) against a human Uniprot database (downloaded on 30 July 2022) using a reverse-decoy method with default settings. A spectral library was generated from the searched data-dependent acquisition (DDA) data using PaSER. Samples were then analyzed by PASEF-dia using a high-speed acquisition scheme from Meier et al.^53^ and searched in real time against the DDA-generated spectral library using the TIMS DIA-NN algorithm with default settings.

Peptide counts were collapsed to the corresponding protein and log-normalized, and fold changes and *z*-scores were calculated over mean signal in term pregnancies. Preterm-specific hits were identified by requiring that at least one preterm pregnancy and no term pregnancies met the threshold of *z* ≥ 5 for a given protein.

### Human placental immunofluorescence

Placental tissue was collected within 2 h of delivery, biopsied as above, embedded, unfixed, in OCT compound (Tissue-Tek) and flash frozen in an isopentanol bath submerged in liquid nitrogen. Ten-micron cryosections were cut from frozen blocks and adhered to glass slides. Slides were thawed and briefly post-fixed with acetone, rehydrated in 1× PBS, blocked with 10% (v/v) goat serum in 1× PBS for 2 h, and incubated with 1:1,000 rabbit anti-human anti-IL1RA (Millipore Sigma catalogue number HPA001482) and 1:500 rat anti-human CD31 (Abcam catalogue number ab9498) overnight at 4 °C in a humidified chamber. Primary antibodies were washed using standard protocols and incubated with anti-rabbit and anti-rat secondaries using standard protocols. Secondary only controls were stained identically but omitting primary antibody. Stained tissue was imaged using the Crest LFOV Spinning Disk/ C2 Confocal at 400× magnification under identical camera exposure and laser settings for secondary only and experimental samples. Micrograph exposure was normalized to secondary only negative control in FIJI and applied to all images at once. In peptide blocking experiments, IL1RA PrEST Antigen (Millipore Sigma APREST83081) was incubated with anti-IL1RA antibody per the manufacturer’s instructions at 4 °C overnight and used to stain tissue as above. In serum blocking experiments, placental sections were blocked with 1% (v/v) goat serum and 10% human serum in PBS-T overnight at 4 C. Controls were blocked without human serum. Placenta was subsequently probed with the commercial anti-IL1RA antibody (Millipore Sigma catalogue number HPA001482) and imaged as above.

### In vitro IL1RA blocking assay

Diluted patient sera, anti-IL1RA antibodies (R&D Systems, AF-280-NA) or anti-GFAP antibodies were pre-incubated with recombinant human IL1RA (PeproTech) for 1 h at 25 °C and then applied to 1 × 10^4^ HEK-Blue IL-1β reporter cell line (InvivoGen) per well for 1 h at 37 °C in 384-well plates. Cells were subsequently stimulated with recombinant human IL-1β (PeproTech) for 24 h at 37 °C. Cells treated with IL-1β alone or TNF alone (PeproTech) served as positive and negative controls, respectively. Cell culture supernatants were assayed using the QUANTI-Blue detection reagent (InvivoGen), as previously described^40,41^. IL-1β and TNF were used at a final concentration of 0.5 ng ml⁻^1^, while IL1RA concentrations were varied as indicated. IL-1β activity was quantified by measuring secreted embryonic alkaline phosphatase (SEAP) activity in cell supernatants at OD_655_ following 3 h of incubation at 37 °C. Raw OD_655_ values were background-subtracted using TNF-treated controls and normalized to the mean signal from IL-1β-only-treated cells, expressed as a percentage. Each serum sample was tested in technical duplicate across an eight-point serum dilution series.

### Mouse Il1ra dot blot

In total, 500 or 250 ng of recombinant mouse Il1ra (R&D 480-RM-050) were blotted onto nitrocellulose using a custom 3D-printed dot blot device under vacuum. Membranes were blocked for 1 h at 25 °C in 5% (w/v) milk and subsequently probed overnight at 4 °C with either anti-human IL1RA antibodies (R&D AF-280-NA or Sigma Prestige HPA001482) or anti-mouse Il1ra antibodies (Invitrogen PA5-21776 or R&D MAB4801) or no primary antibody. Following standard washing procedures, membranes were probed with relevant secondaries conjugated to Licor-IR dyes and blots were imaged using the Licor Odyssey machine. Membranes were imaged together in one scan under identical exposure settings.

### Luminex assay to detect anti-IL1RA antibodies in patient sera

Recombinant IL1RA (Peprotech) or BSA (Thermo) were conjugated to spectrally-distinct Luminex beads in separate 1.5-ml protein LoBind tubes. Each bead conjugation was performed as previously described^54^ using the Antibody Coupling Kit following manufacturer’s instructions (Luminex, 40-50016). All serological analyses were performed exactly as previously described^54^ in technical duplicate on two separate experimental days. Putatively positive samples (with more than 20 net mean fluorescence intensity (MFI) of IL1RA/BSA) were repeated on an additional experimental day in technical duplicate. All net MFI IL1RA/BSA values were averaged, and *z*-scores above the mean were calculated for term pregnancies.

### Statistics and reproducibility

Detailed statistical analysis of PhIP-seq data is available in the Supplementary Methods. No statistical method was used to predetermine sample size. Statistical analyses were performed as described for each experiment, with technical replicates and independent cohorts used where indicated. Data were excluded only according to predefined quality-control, eligibility or missing-data criteria as described in the Supplementary Methods. Data distribution was assumed to be normal, but this was not formally tested. The experiments were not randomized. No animals were excluded from analysis. Investigators were not blinded to allocation during experiments and outcome assessment, except for blinded histopathological assessment of mouse H&E sections.

### Reporting summary

Further information on research design is available in the Nature Portfolio Reporting Summary linked to this article.

## Online content

Any methods, additional references, Nature Portfolio reporting summaries, source data, extended data, supplementary information, acknowledgements, peer review information; details of author contributions and competing interests; and statements of data and code availability are available at https://doi.org/10.1038/s41590-026-02689-y.

## Supplementary information

Supplementary Information Supplementary Fig. 1 and Methods.

Reporting Summary

Peer Review File

Supplementary Table 1 Sample and cohort statistics.

Supplementary Table 2 The PhIP-seq pregnancy signature.

Supplementary Table 3 The preterm and term autoantibody signature.

Supplementary Table 4 Feature coefficients of the final logistic reggression model to predict preterm outcome.

Supplementary Table 5 IP–MS autoantibody targets and overlap with PhIP-seq.

Supplementary Table 6 Samples tested for IL1RA across orthogonal methods.

## Source data

Source Data Fig. 1 Statistical source data.

Source Data Fig. 2 Statistical source data.

Source Data Fig. 3 Statistical source data.

Source Data Fig. 4 Statistical source data.

Source Data Fig. 5 Statistical source data.

Source Data Fig. 6 Statistical source data.

Source Data Fig. 7 Statistical source data.

Source Data Fig. 8 Raw microscopy images.

Source Data Extended Data Fig. 1 Statistical source data.

Source Data Extended Data Fig. 2 Statistical source data.

Source Data Extended Data Fig. 4 Statistical source data.

Source Data Extended Data Fig. 5 Statistical source data.

Source Data Extended Data Fig. 6 Statistical source data.

Source Data Extended Data Fig. 7 Statistical source data.

Source Data Extended Data Fig. 8 Statistical source data.

Source Data Extended Data Fig. 9 Statistical source data, dot blot raw data and raw image of mouse experiment.

Source Data Extended Data Fig. 10 Statistical source data and raw microscopy images.

## Data Availability

All raw and processed data for cohorts II–VIII as well as trained models and feature weights are available via Dryad in the associated repository https://doi.org/10.5061/dryad.fqz612k24 (ref. ^55^). Data for Cohort I were obtained via agreement with the California State Committee for the Protection of Human Subjects which prohibits the depositing of any individual level data into any external data repository where it could be used by unapproved investigators even if that data could not be used to identify individual subjects. Requests for access to the Cohort I individual-level data should be directed to L.L.J.P. Access is subject to approval by the California State Committee for the Protection of Human Subjects and completion of any required data use agreements, which restrict use of the data to approved investigators and approved research purposes. Requests will be reviewed and responded to within a reasonable timeframe following receipt of the required documentation. Contact details and instructions for submitting an access request are provided in the Dryad repository^55^. The mass spectrometry proteomics data have been deposited to the ProteomeXchange Consortium via the PRIDE partner repository with the dataset identified PXD085007. Correspondence and requests for materials should be addressed to J.L.D., joe@derisilab.ucsf.edu. Source data are provided with this paper.

## Code availability

PhIP-seq analytical code is freely available via GitHub at https://github.com/h-s-miller/phagepy.
