# Supplementary material for: Evidence for maternal autoantibodies in the pathogenesis of preterm birth
Source: Nat Immunol. 2026 Oct 14;27(11):2280–92. doi: 10.1038/s41590-026-02689-y (PMC13710164; doi:10.1038/s41590-026-02689-y)
Supplement: Supplementary file 1 [file 41590_2026_2689_MOESM1_ESM.pdf]

nature immunology

Supplementary information https://doi.org/10.1038/s41590-026-02689-y

Evidence for maternal autoantibodies in the pathogenesis of preterm birth

In the format provided by the authors and unedited

Supplementary Data Figure 1

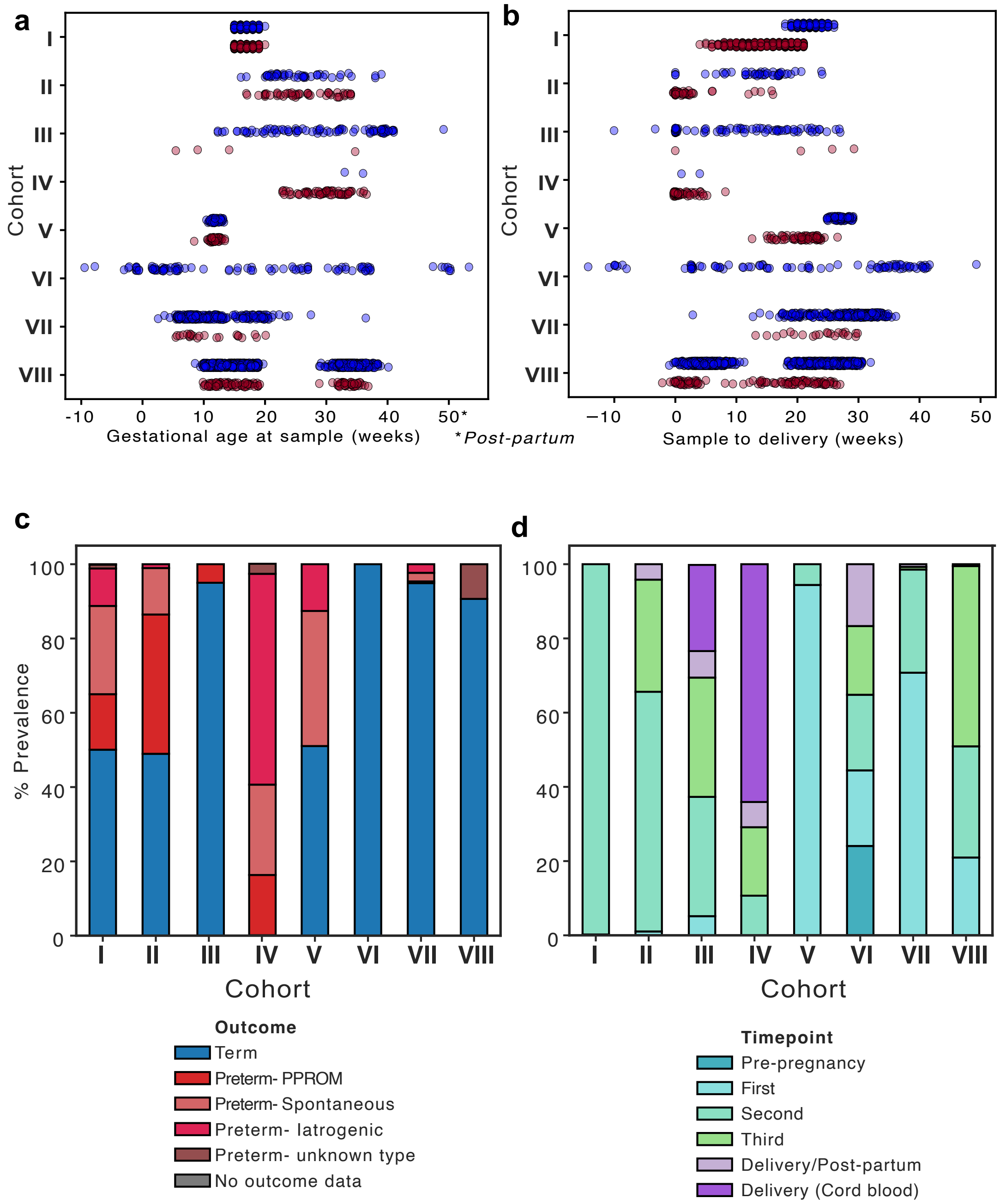

Supplementary Data Figure 1. **Maternal serum samples used in the study** Dot plot of gestational age **a.** at test or **b.** sample to delivery in weeks across the eight pregnancy cohorts with term (blue) and preterm (red) outcomes. Stacked barplots of **c.** pregnancy outcomes and **d**. timepoints at sample collection for eight pregnancy cohorts. Individual human samples represent each dot in a and b.

SUPPLEMENTARY METHODS

I. PhIP-seq Bioinformatic Analyses

To analyze peptide enrichment after PhIP-seq, reads were aligned at the protein level using RAPsearch[1], as previously described[2]. Aligned reads were normalized to 100,000 reads per k-mer (RPK) to account for varying read-depth. All downstream analyses were performed by using an implementation of PhagePy python package (https://github.com/h-s-miller/phagepy).

All data was initially filtered to samples with greater than recovered 100,000 reads. Data was pseudocounted and fold change over mock IP controls was calculated. Co-correlations of the fold change over mock IP matrix were used to determine technical replicate consistency. Subsequently, technically replicated samples (with the exception of positive and mock IP controls) were averaged.

Fold change over mock IP was re-calculated with averaged values. Additional fold changes were calculated over mean in healthy term pregnancies from Cohort I only (see above) or preterm pregnancies (downsampled to n=204, five times, then averaged). Z-scores were calculated for each of the fold-change matrixes after log-10 transformation relative to mock IP, healthy term pregnancies, and preterm pregnancies. Peptides with a z-score greater than 3 in a minimum of >0.5% experimental samples and <0.5% control samples were considered specific for that comparison, unless otherwise indicated. Analysis was performed on all cohorts together, except when performing comparisons for Cohort II PhIP-seq data with Cohort II IP-MS data, where the same analysis was performed using samples only from Cohort II. Positional enrichment analysis across IL1RA was performed as previously described[3] for Cohort I samples only and preterm-specific hits were recalculated as above with data from this cohort only. Autoreactivities were counted per person by summing up binarized per peptide hits within the preterm- or term-specific PhIP-seq signature. Where indicated, cumulative enrichment was calculated by summing log-transformed enrichments over mock IPs for peptides identified as preterm- or term-specific. Gene-set enrichment analysis was performed by manually annotating genes with previously reported roles in placental function and pregnancy. GO term enrichment analysis supplemented manual grouping and was performed utilizing the gseapy package.

II. Logistic Regression Model of Pregnancy

Feature selection

Pregnancy-specific peptides were defined using the FET cohort (Cohort VI) exclusively: peptide-level fold changes were computed for each individual relative to their pre-pregnancy baseline. Peptides were retained if they achieved a z-score ≥ 3 in at least 10 pregnancy samples and were absent (z-score < 3) from all baseline samples.

Data preparation and cohort inclusion

Samples from nine cohorts (Cohort I, II, III, IV, V, VII, VII, Healthy Baseline 1, Healthy Baseline 2) were included in the classification analysis. Cohort VI, used solely for feature selection, was excluded from model training and evaluation to prevent

information leakage. The binary outcome variable encoded pregnancy status: pregnant samples were labeled as positive and non-pregnant samples (nulligravid females and males from healthy control cohorts) as negative.

Input matrix

The input feature matrix consisted of $\log_{10}$-transformed fold changes over mock immunoprecipitation controls (stored as `log_FC_over_AG` in the AnnData object), restricted to the 3,096 pregnancy-associated peptides defined above. This matrix captures the magnitude of autoreactive enrichment for each peptide relative to background signal.

Cross-validation and model training

A five-fold cross-validation procedure was used to generate out-of-fold predicted probabilities. To prevent data leakage from longitudinally sampled individuals, all samples from a given subject (identified by barcode) were constrained to the same fold. Within each training fold, the following steps were applied:

1. *Fold-specific baseline normalization.* Using only the training-fold samples, an adjudicated term-control baseline was computed by averaging fold-change values across healthy term controls. Log_{10}-transformed fold changes were then recalculated relative to this training-fold baseline for all samples in the fold, ensuring that no information from held-out validation samples influenced normalization.
2. *Feature scaling* Selected peptide features were standardized (z-scored without centering, to accommodate sparse inputs) using a `StandardScaler` fitted only on the training portion of each fold.
3. *Classifier fitting.* A logistic regression model with elastic net regularization (L1 ratio = 0.2, regularization strength C = 1.0, solver = SAGA) and balanced class weights was fitted on the scaled training-fold matrix. The maximum number of iterations was set to 10,000 to ensure convergence. Predicted probabilities for the held-out validation fold were recorded.

This procedure was repeated across all five folds, yielding out-of-fold predicted probabilities for every sample in the training set.

Evaluation

Classifier performance was assessed by computing the area under the receiver operating characteristic curve (ROC AUC) from the out-of-fold predicted probabilities across the full cross-validated dataset. For all reported AUC values, 95% confidence intervals were computed using the bootstrap percentile method (2,000 iterations) and reported as asymmetric ranges. Statistical significance of each AUC against the null hypothesis of AUC = 0.5 was assessed using the DeLong one-sample test.

Implementation

All analyses were performed in Python using AnnData for data management and scikit-learn (v1.x) for model fitting and evaluation. Randomness entered through the cross-

validation fold assignment (seed = 0). No hyperparameter tuning was performed; regularization parameters were fixed a priori. The full analytical code is available at https://github.com/h-s-miller/phagepy.

III. Sub-analysis of the Preterm Autoantibody Signature

Visualization of preterm autoantibody signature across gestational age

Peptides were selected from the preterm autoantibody signature by first filtering to the top 5,000 peptides ranked by $\log_2$ fold change (preterm over healthy term), then selecting the 500 most prevalent (by number of preterm samples enriched). Genes were similarly ranked by selecting the top 2,000 genes with the fewest enrichments in healthy term controls, then retaining the 500 most prevalent in preterm samples.

Peptides (rows) were clustered across all samples using the Jaccard distance metric with complete linkage. Total number of significant peptide hits per sample (summed from the binary hit matrix), providing a per-group measure of overall autoreactive breadth. A right-side summary panel shows the mean proportion of samples positive for each peptide within each outcome-by-trimester group.

Identification of reactivities associated with preterm clinical subsets

Peptides within the preterm autoantibody signature were mapped to their corresponding genes using the phage library annotation. For each sample, gene-level enrichment was computed as the sum of peptide-level fold changes over healthy term controls (calculated relative to healthy term pregnancies from Cohort I) across all preterm autoantibody peptides mapping to a given gene. This summation approach captures cumulative autoreactivity against a protein, accounting for genes represented by multiple peptides in the library. Seven pairwise comparisons were defined within preterm samples: (i) spontaneous versus medically-indicated preterm delivery, (ii) history of prior preterm birth versus no history, (iii) preeclampsia versus no preeclampsia, (iv) early preterm (gestational age at delivery < 28 weeks) versus late preterm (32–37 weeks), (v) moderate preterm (28–32 weeks) versus late preterm (32–37 weeks), (vi) multigravidae (gravidity ≥ 2) versus primigravidae, and (vii) high gravidity (gravidity ≥ 4) versus primigravidae. Comparisons were retained for analysis only if both groups contained at least 10 samples.

For each gene and each comparison, the distributions of gene-level summed enrichments between the two groups were compared using both the two-sample Kolmogorov–Smirnov test. P-values were corrected for multiple testing across genes within each comparison using the Benjamini–Hochberg procedure. Log_2 fold changes were computed as $\log_2((\text{mean}_1 + 1) / (\text{mean}_2 + 1))$, where mean_1 and mean_2 are the mean gene-level enrichments in the two groups, respectively. The direction of effect (which group exhibited higher enrichment) was recorded for each gene. To ensure that significant associations were driven by a meaningful number of individuals rather than outlier values, we applied robustness criteria. For each gene, the group with higher mean enrichment was identified, and an enrichment threshold was defined as the 75th percentile of gene-level enrichment values in the lower group. A gene was classified as "robust" if at least 5 samples in the higher group exceeded this threshold and these samples constituted at least 10% of the higher group. A composite rank score was computed for robust genes as: $-\log_{10}(\text{p-value}) \times |\log_2\text{FC}| \times \sqrt{(\text{n\_positive})}$, where

n_positive is the number of samples in the higher group exceeding the threshold. Non-robust genes received a rank score of zero.

To summarize associations across comparisons, a heatmap was constructed displaying the top 50 genes ranked by their maximum robust rank score across all comparisons. For each gene–group pair, the raw p-value was displayed if the gene was significantly enriched in that group ($p < 0.05$ and the group exhibited higher mean enrichment). Where a single group served as a reference for multiple comparisons (e.g., "Late" preterm for both early-vs-late and moderate-vs-late), the most significant p-value across those comparisons was displayed.

Analysis of Preterm Birth Risk Factors

Logistic regression analyses were performed using the earliest available gestational sample per patient, deduplicated by patient identifier to ensure statistical independence. For each sample, the cumulative PhIP-seq enrichment score was computed as the sum of $\log_2$ fold changes (preterm over healthy term) across peptides in the preterm autoantibody signature. Scores were calculated at multiple stringency levels by restricting to the top 10, 50, 100, 200, or 500 peptides ranked by fold change, as well as across all signature peptides. All PhIP-seq scores and continuous clinical variables (maternal age, gravidity) were standardized to zero mean and unit variance prior to modeling, so that odds ratios reflect the effect of a one standard deviation increase.

Separate logistic regression models were fit for each clinical variable (history of prior preterm birth, preeclampsia, maternal age, and gravidity) and each PhIP-seq enrichment score, with preterm birth as the binary outcome. Odds ratios and 95% Wald confidence intervals were computed from the estimated coefficients.

A multivariate logistic regression model was fit including both history of preterm birth and the PhIP-seq top-100 peptide score as predictors. To estimate the combined effect of both risk factors, we computed the joint log-odds for a patient with a positive history of preterm birth and elevated autoantibody burden at +1 and +2 standard deviations above the mean, with confidence intervals computed using the delta method to account for covariance between parameter estimates. Results were visualized as a forest plot of odds ratios with 95% confidence intervals. All models were fit using the statsmodels Python package.

IV. Machine Learning Predictive Modelling of Preterm Delivery

Data and preprocessing

A total of 3,073 samples from eight independent cohorts (Cohorts I–VIII) were included in the preterm birth prediction analysis. The binary outcome variable encoded preterm birth status: samples delivered before 37 weeks of gestation were labeled as positive (preterm) and those delivered at or after 37 weeks as negative (term). The input feature matrix consisted of binarized peptide enrichment calls from the PhIP-seq analysis over mock IP. For each sample, a peptide was scored as a “hit” (1) if its fold change over mock immunoprecipitation controls exceeded a z-score threshold of 3, and 0 otherwise, yielding a sparse binary matrix of 731,724 peptide features. For cohorts with longitudinal sampling, technical replicates were deduplicated at the barcode (subject) level to ensure statistical independence.

Train/holdout partitioning

Samples were split once into training (~85%) and holdout (~15%) sets (random seed = 666). The split was stratified by outcome and constrained by cohort and gestational-age-at-sampling bins (12, 16, 20, 24, 28, 32, 36, and 40 weeks). For longitudinal cohorts, a subject identifier (barcode) was used to prevent data leakage by keeping all samples from the same individual within a single partition. This produced 2,607 training samples and 466 holdout test samples. All model development and cross-validation used only the training partition; final performance was assessed once on the untouched holdout set.

Per-cohort consensus feature selection

To select peptide features that generalize across cohorts rather than reflecting batch-specific signals, we implemented a per-cohort consensus feature selection procedure. Feature selection was restricted to “powered” cohorts, defined as those with at least 10 preterm cases and 10 term controls in the training partition; five of the eight cohorts met this criterion (Cohorts I, II, V, VII, VIII). For each powered cohort, barcode-deduplicated prevalence (the fraction of unique subjects with a hit for a given peptide) was computed separately for preterm and term samples. The prevalence difference (preterm prevalence minus term prevalence) was calculated for each peptide, and peptides were ranked by the absolute value of this difference within each cohort. The top K = 30,000 peptides per cohort were retained. A consensus threshold of C = 2 was applied: only peptides appearing in the top-K list of at least two independent cohorts were selected. Each consensus peptide was assigned a direction—classified as preterm-enriched if at least two cohorts showed positive enrichment, or term-enriched if at least two showed negative enrichment. This procedure yielded 12,071 consensus peptides (6,779 preterm-enriched and 5,292 term-enriched).

Protein-level feature aggregation and selection

To capture protein-level autoreactive signal, peptides were mapped to their corresponding protein using the phage library annotation (731,724 peptides mapping to 19,997 unique protein). For each sample, a protein-level hit count was computed as the sum of peptide-level binary hits across all peptides mapping to that protein. Protein-level features were binarized at a threshold of ≥2 peptide hits per protein. Per-cohort protein enrichment was computed using the same prevalence-difference framework as for peptides, and protein consensus selection was applied with K = 500 and C ≥ 3 (requiring a protein to appear in the top-500 list of at least three cohorts with consistent direction). One protein (SPATA6) survived this stringent filtering, yielding a total of 12,072 selected features (12,071 peptides + 1 gene).

Feature normalization and scaling

To account for differences in total autoreactive burden across samples and batches, each sample’s binary peptide hit counts were normalized by dividing by the total number of peptide hits for that sample (ss_{ij} / $total\_hits_i$), converting raw hit counts into a “share of total reactivity” representation. This normalization renders features approximately batch-invariant, as between-cohort differences in overall reactivity are removed. Protein-level

features were similarly normalized by total protein hits per sample. The combined peptide and protein feature matrix was scaled using a maximum-absolute-value scaler (MaxAbsScaler), which preserves the sparsity structure of the input matrix.

Classifier and hyperparameters

The classifier was a logistic regression model with elastic net regularization (scikit-learn LogisticRegression, solver = SAGA). Regularization parameters were set to C = 0.18 and L1 ratio = 0.90, selected from a grid search over $C \in \{0.02, 0.04, 0.07, 0.10, 0.12, 0.15, 0.18, 0.20\}$ and L1 ratio $\in \{0.85, 0.90, 0.95\}$, optimized on a 3-fold stratified cross-validation (grouped by subject barcode) within the training set. The maximum number of iterations was set to 10,000 with a convergence tolerance of 10^{-4}. Inverse class-frequency weighting (class_weight = “balanced”) was applied to account for outcome imbalance. The fitted model retained 463 non-zero coefficients (462 peptide features and 1 gene feature) out of 12,072 candidate features, providing an interpretable set of signed per-feature weights.

Cross-validation

Model performance was estimated by 5-fold stratified group cross-validation on the training set (StratifiedGroupKFold, grouped by subject barcode, shuffle seed = 42). For all reported AUC values, 95% confidence intervals were computed using the bootstrap percentile method (2,000 iterations) and reported as asymmetric ranges. Statistical significance of each AUC against the null hypothesis of AUC = 0.5 was assessed using the DeLong one-sample test.

Holdout evaluation and subgroup analyses

The trained model was evaluated on the held-out test set (466 samples). We computed the overall ROC AUC with 95% bootstrap confidence intervals (2,000 iterations). Per-cohort ROC AUC was computed for each evaluable cohort in the test set (those with at least one preterm and one term sample). To examine the model’s sensitivity to preterm severity, we performed a severity-stratified analysis: preterm test samples were grouped by gestational age at delivery into very early preterm (<28 weeks), early preterm (28–32 weeks), moderate preterm (32–34 weeks), and late preterm (34–37 weeks), and each group was evaluated against all term controls (≥37 weeks). Additional clinical subgroup analyses evaluated model performance across preterm subtypes (iatrogenic versus spontaneous preterm delivery) and gravidity categories (primigravida, 2–3 pregnancies, and ≥4 pregnancies), where clinical metadata were available.

Reproducibility and implementation details

All analyses were performed in Python (v3.10) using AnnData for matrix management and scikit-learn (v1.5) for model building. Randomness entered through the train/holdout split seed (666) and the cross-validation shuffle seed (42); all seeds are specified above. Feature selection was performed entirely within the training partition using only powered cohorts, with no information from the holdout set. All reported cross-validated metrics were computed strictly within the training partition. Holdout metrics were computed once on the untouched test set. The trained model, feature indices, scaler, and all 463 feature coefficients were saved as a portable model bundle. All raw and

processed data, the trained model and feature weights, and the associated code are available for download at Dryad.

SUPPLEMENTARY REFERENCES

1. Zhao, Y., Tang, H. & Ye, Y. RAPSearch2: a fast and memory-efficient protein similarity search tool for next-generation sequencing data. *Bioinformatics* **28**, 125–126 (2012).
2. Vazquez, S. E. *et al.* Identification of novel, clinically correlated autoantigens in the monogenic autoimmune syndrome APS1 by proteome-wide PhIP-Seq. *eLife* **9**, 1–25 (2020).
3. Rajan, J. V. *et al.* Phage display demonstrates durable differences in serological profile by route of inoculation in primary infections of non-human primates with Dengue Virus 1. *Sci. Rep.* **11**, 1–12 (2021).
