# Supplementary material for: Evidence for maternal autoantibodies in the pathogenesis of preterm birth
Source: Nat Immunol. 2026 Oct 14;27(11):2280–92. doi: 10.1038/s41590-026-02689-y (PMC13710164; doi:10.1038/s41590-026-02689-y)
Supplement: Supplementary file 2 [file 41590_2026_2689_MOESM2_ESM.pdf]

nature portfolio

Corresponding author(s): DeRisi, Joseph L

Last updated by author(s): Sept 3, 2026

Reporting Summary

Nature Portfolio wishes to improve the reproducibility of the work that we publish. This form provides structure for consistency and transparency in reporting. For further information on Nature Portfolio policies, see our Editorial Policies and the Editorial Policy Checklist.

Statistics

For all statistical analyses, confirm that the following items are present in the figure legend, table legend, main text, or Methods section.

n/a	Confirmed	
☐	☒	The exact sample size (n) for each experimental group/condition, given as a discrete number and unit of measurement
☐	☒	A statement on whether measurements were taken from distinct samples or whether the same sample was measured repeatedly
☐	☒	The statistical test(s) used AND whether they are one- or two-sided<br>*Only common tests should be described solely by name; describe more complex techniques in the Methods section.*
☐	☒	A description of all covariates tested
☒	☐	A description of any assumptions or corrections, such as tests of normality and adjustment for multiple comparisons
☐	☒	A full description of the statistical parameters including central tendency (e.g. means) or other basic estimates (e.g. regression coefficient) AND variation (e.g. standard deviation) or associated estimates of uncertainty (e.g. confidence intervals)
☐	☒	For null hypothesis testing, the test statistic (e.g. F, t, r) with confidence intervals, effect sizes, degrees of freedom and P value noted<br>*Give P values as exact values whenever suitable.*
☒	☐	For Bayesian analysis, information on the choice of priors and Markov chain Monte Carlo settings
☒	☐	For hierarchical and complex designs, identification of the appropriate level for tests and full reporting of outcomes
☐	☒	Estimates of effect sizes (e.g. Cohen's d, Pearson's r), indicating how they were calculated

Our web collection on statistics for biologists contains articles on many of the points above.

Software and code

Policy information about availability of computer code

Data collection	No data collection software was used.
Data analysis	PhIP-seq analytical code is freely available on GitHub: https://github.com/h-s-miller/phagepy

For manuscripts utilizing custom algorithms or software that are central to the research but not yet described in published literature, software must be made available to editors and reviewers. We strongly encourage code deposition in a community repository (e.g. GitHub). See the Nature Portfolio guidelines for submitting code & software for further information.

Data

Policy information about availability of data

All manuscripts must include a data availability statement. This statement should provide the following information, where applicable:

- Accession codes, unique identifiers, or web links for publicly available datasets
- A description of any restrictions on data availability
- For clinical datasets or third party data, please ensure that the statement adheres to our policy

All raw and processed data, the forecasting model and feature weights are available for download at Dryad at DOI: 10.5061/dryad.fqz612k24.

Research involving human participants, their data, or biological material

Policy information about studies with human participants or human data. See also policy information about sex, gender (identity/presentation), and sexual orientation and race, ethnicity and racism.

Reporting on sex and gender	Biological sex was reported female for Cohorts I-VIII. Biological sex of the infant (male or female categories) was available and statistical analysis between this variable and delivery outcome are reported in Supplementary Table 1, when available. Infant sex categories were obtained from patient medical records. The non-pregnant Baseline Cohorts were comprised of non-pregnant healthy individuals with an equal balance of biological sex (male and female categories). Sex in this cohort was self-reported. Gender and/or sexual orientation data was not available for any of the cohorts utilized. Sex chromosome information was not available for any of the cohorts utilized.
Reporting on race, ethnicity, or other socially relevant groupings	Self-reported race and ethnicity was recorded for Cohorts I, II, III, IV, and VII. Distribution of these variables was significant across cases and controls, as reported in Supplementary Table 1. Racially and ethnically diverse cohorts were recruited, representative of populations from which the cohorts were drawn. The Baseline Cohort was comprised of individuals across all decades of life and self-reported race and ethnicity categories are reflective of New York, NY. Racial and ethnic distribution of Cohort I reflects California demographics (predominantly Hispanic). Cohorts II, III, and VII represent demographics in San Francisco, CA. Cohort IV is representative of demographics in London, UK and self-reported racial and ethnic categories differ from North American cohorts. Genetic ancestry data was not available for any cohort. While subject-level race and ethnicity was not available fro Cohort V, it was comprised of participants living in Italy, providing yet another layer of geographic diversity in our work.
Population characteristics	Maternal age, delivery mode, gravidity, and infant sex were collected for Cohorts I-VII and did not vary significantly between cases and controls, as described in Supplementary Table 1.
Recruitment	Cohort I was assembled retrospectively by drawing from a California biobank, balancing preterm and term outcomes. Participants in Cohort II, III, VI, and VII were recruited from the population of patients delivering at UCSF. Participants in Cohort IV were recruited from the population of patients delivering at UCL Hospital and Homerton University Hospital of the National Health Services of the United Kingdom. Baseline Cohortst were healthy blood donors at the New York Blood Center and at UCSF.
Ethics oversight	Human subjects research was conducted with ethical oversight from the following commitees under the described protocol numbers. Sera from the Cohort I was obtained from a California-wide biobank (Committee for the Protection of Human Subjects within the Health and Human Services Agency of the State of California protocol# 12-09-0702). Sera from the Cohort II, Cohort III, Cohort VI, and Cohort VII (UCSF IRB# 10-00505, 20-31171, 20-32077, 20-32779, 16-20474; San Francisco, CA) was obtained from deliveries at UCSF. Sera from Cohort IV (UCSF IRB# 10-00350) was obtained from UCL Hospital and Homerton University Hospital of the National Health Services of the United Kingdom. Sera from Cohort V was obtained from 'Fondazione IRCCS Policlinico San Matteo' (San Matteo Research Hospital) in Pavia, Italy, under RC08061819 and RC08061821 approved IRB protocols. Sera from Cohort VIII (IRB# 2009P000557 and 2014P001109, Boston, MA) was obtained from Brigham and Women's Hospital in Boston, MA, USA from the previously described VDAART clinical trial59. De-identified healthy control (non-pregnant) plasma was collected from two sources: courtesy of New York Blood Center (New York, NY) and donors from a UCSF community drive (UCSF IRB# 22-3611; San Francisco, CA). All samples were collected under the referenced Institutional Review Board protocols, and all patients gave informed consent prior to sample collection.

Note that full information on the approval of the study protocol must also be provided in the manuscript.

Field-specific reporting

Please select the one below that is the best fit for your research. If you are not sure, read the appropriate sections before making your selection.

☒ Life sciences ☐ Behavioural & social sciences ☐ Ecological, evolutionary & environmental sciences

For a reference copy of the document with all sections, see nature.com/documents/nr-reporting-summary-flat.pdf

Life sciences study design

All studies must disclose on these points even when the disclosure is negative.

Sample size	Sample size calculation was not performed for experiments involving retrospective human cohorts. Experiments with pregnant mice were performed without a priori sample size calculation. These experiments yielded significantly different outcomes, indicating further animals were not required.
Data exclusions	Samples that did not reach a minimum of 100,000 reads per sample in PhIP-seq experiments were excluded from the analysis. If no sera remained, that subject was dropped from subsequent analysis.
Replication	Technically replicated samples were included in every PhIP-seq experiment and exhibited high co-correlation, as described in Extended Data Fig 1b. Murine experiments were performed in experimental triplicate with separate cohorts of pregnant mice. In vitro IL1b activity assays were performed at least twice in experimental triplicates. Luminex experiments were performed in technical duplicate on two separate days. Positive Luminex samples were then repeated on a third separate day. Two samples in IP-MS experiments were replicated.
Randomization	Samples were randomized utilizing the Hamilton STAR robotic liquid handling platform. Operators were not aware of preterm or term

Randomization	outcome.<br><br>Samples with either term or preterm outcomes were randomly distributed across experimental plates. Batch effect was not observed across experimental plates.
Blinding	Experimental operators were blinded to preterm or term outcomes during the execution of PhIP-seq, IIP-MS, and Luminex experiments with human cohorts. Analysis was unblinded, as model training was supervised. Mouse experiments and in vitro IL1b activity experiments were unblinded.

Reporting for specific materials, systems and methods

We require information from authors about some types of materials, experimental systems and methods used in many studies. Here, indicate whether each material, system or method listed is relevant to your study. If you are not sure if a list item applies to your research, read the appropriate section before selecting a response.

Materials & experimental systems

n/a	Involved in the study
☐	☒ Antibodies
☐	☒ Eukaryotic cell lines
☒	☐ Palaeontology and archaeology
☐	☒ Animals and other organisms
☒	☐ Clinical data
☒	☐ Dual use research of concern
☒	☐ Plants

Methods

n/a	Involved in the study
☒	☐ ChIP-seq
☒	☐ Flow cytometry
☒	☐ MRI-based neuroimaging

Antibodies

Antibodies used	Goat anti-human Anti-IL1RA (R&D AF-280-NA), rabbit anti-human Anti-IL1RA (Millipore Sigma Cat No. HPA001482), rabbit ant-human Anti-GFAP (Agilent Z033429-2), rat anti-human Anti-CD31 (Abcam Cat. No. ab9498), mouse ant-human Anti-CD144 (Ve-cadherin; BD Pharmingen 555661), rabbit anti-mouse Il1ra (Invitrogen PA5-21776), rat anti-mouse Il1ra (R&D MAB4801). Secondary antibodies raised in goat : Anti-Rabbit Alexa 647 (Invitrogen A-21244), anti-Mouse Alexa 568 (Invitrogen A11031), anti-Rat TRITC (Abcam ab7094), anti-Rabbit 800CW (LICOR 925-32211), anti-Goat 800CW (LICOR 926-32214), anti-Rat 800CW (LICOR 926-32219), and anti-Human HRP (Invitrogen A18805). Goat IgG isotype control (R&D AB-108-C).
Validation	Both commercial anti-IL1RA antibodies were extensively validated with dot blot, PhIP-seq, and antigen blocking experiments on human placenta, as described in the manuscript. Anti-GFAP antibodies were validated with PhIP-seq, as previously described. All antibody stains were performed with a secondary only control.

Eukaryotic cell lines

Policy information about cell lines and Sex and Gender in Research

Cell line source(s)	HEKBlue IL1b reporter cells were obtained from InvivoGen Cat No. hkb-il1bv2.
Authentication	Cell line was authenticated by the manufacturer and confirmed to be stimulated by recombinant IL1b but not TNFa
Mycoplasma contamination	Cells were confirmed mycoplasma negative from the manufacturer.<br>Cells were passaged to a maximum of eight passages. Further mycoplasma testing was not performed.
Commonly misidentified lines<br>(See ICLAC register)	Commonly misidentified lines were not used.

Animals and other research organisms

Policy information about studies involving animals; ARRIVE guidelines recommended for reporting animal research, and Sex and Gender in Research

Laboratory animals	Timed-pregnant C57/Bl6 mice were obtained from Jackson Labs.
Wild animals	Wild animals were not utilized
Reporting on sex	Sex-based analysis was not performed, because all mice were female. This goal of this study is to investigate the fundamental biology of pregnancy, as such male mice were not studied.
Field-collected samples	Field-collected samples were not utilized.

Ethics oversight | Animal experiments were performed under UCSF IACUC approved protocols.

Note that full information on the approval of the study protocol must also be provided in the manuscript.

Plants

Seed stocks | Plants were not utilized

Novel plant genotypes | Plants were not utilized

Authentication | Plants were not utilized
