# Supplementary material for: Evidence for maternal autoantibodies in the pathogenesis of preterm birth
Source: Nat Immunol. 2026 Oct 14;27(11):2280–92. doi: 10.1038/s41590-026-02689-y (PMC13710164; doi:10.1038/s41590-026-02689-y)
Supplement: Supplementary file 3 [file 41590_2026_2689_MOESM3_ESM.pdf]

nature portfolio

Peer Review File

Evidence for Maternal Autoantibodies in the Pathogenesis of Preterm Birth

Corresponding Author: Dr Joseph DeRisi

This file contains all editorial decision letters in order by version, followed by all author rebuttals in order by version.

Version 0:

Decision Letter:

28th Apr 2026

Dear Joe,

Thank you for transferring your manuscript entitled "Evidence for Maternal Autoantibodies in the Pathogenesis of Preterm Birth". Apologies for the delay in getting back to you. We had our editorial team meeting this morning and I was able to discuss your study findings with the others on our editorial team.

We had previously discussed the study with Ashley at Nature Medicine, who had consulted with us last week. More importantly, we saw that the manuscript had been reviewed at Nature three times, so we were able to obtain those previous reviewer comments. I had the impression that two of the referees were more hung up attempting to generalize the findings regarding causation for pre-term births, rather than noting that a subset of pregnant mothers who experienced pre-term births harbored autoantibodies against IL-1 receptor antagonist.

We are very interested in the possibility of publishing your study in Nature Immunology, but would like you revise with the following suggestions. Given that the manuscript is written in an abbreviated form to fit within the Nature article format, I suspect that the data presentation and discussion of the findings could be improved. For example, there are 3 main figures and 16 Extended Data figures. Note, research articles for Nature Immunology can have up to 8 main figures and 10 Extended Data figures. The word length for the combined Introduction-Results-Discussion can be up to 5000 words, however the Abstract should not be longer than 150 words total.

I suspect that the data presented in these figures can be reorganized, eg bring some Extended data into main figures (noting to try not to exceed more than 10-12 panels per figure) to provide more clarity. Two, there's a bit more room to expand on provide more context or discussion of the results, especially to emphasize the immunological aspects insights gained in the new study.

We therefore invite you to revise your manuscript taking into account all reviewer and editor comments. Please highlight all changes in the manuscript text file in Microsoft Word format.

We may chose to have a systems immunology expert look at the revised manuscript, given that there was a section noting data analysis from PhIP-seq discovery platform to build modeling for identifying signatures of enrichment for peptide epitope sequences recognized by autoantibodies identified in pre-term births. That is, building training sets by omitting data one or more cohorts, then testing the performance of the predictions in the omitted cohort dataset to determine the robustness of the signatures.

We are committed to providing a fair and constructive peer-review process. Do not hesitate to contact us if there are specific requests from the reviewers that you believe are technically impossible or unlikely to yield a meaningful outcome.

When revising your manuscript:

* Include a “Response to referees” document detailing, point-by-point, how you addressed each referee comment. If no action was taken to address a point, you must provide a compelling argument.

* If you have not done so already please begin to revise your manuscript so that it conforms to our Article format instructions at http://www.nature.com/ni/authors/index.html. Refer also to any guidelines provided in this letter.

* Please include a revised version of any required reporting checklist. It will be available to referees to aid in their evaluation of the manuscript goes back for peer review. They are available here:

Reporting summary:
https://www.nature.com/documents/nr-reporting-summary.pdf

When submitting the revised version of your manuscript, please pay close attention to our href="https://www.nature.com/nature-portfolio/editorial-policies/image-integrity">Digital Image Integrity Guidelines. and to the following points below:

-- that unprocessed scans are clearly labelled and match the gels and western blots presented in figures.
-- that control panels for gels and western blots are appropriately described as loading on sample processing controls
-- all images in the paper are checked for duplication of panels and for splicing of gel lanes.

Please note, Extended Data figures and tables are online-only (appearing in the online PDF and full-text HTML version of the paper), peer-reviewed display items that provide essential background to the Article but are not included in the printed version of the paper due to space constraints or being of interest only to a few specialists. A maximum of ten Extended Data display items (figures and tables) is typically permitted. When re-submitting your manuscript, please ensure that any supplementary figures and tables that are more critical to the manuscript's conclusions are converted to Extended data to increase these data's visibility.

Finally, please ensure that you retain unprocessed data and metadata files after publication, ideally archiving data in perpetuity, as these may be requested during the peer review and production process or after publication if any issues arise.

Please use the link below to submit your revised manuscript and related files:

Link Redacted

Note: This URL links to your confidential home page and associated information about manuscripts you may have submitted, or that you are reviewing for us. If you wish to forward this email to co-authors, please delete the link to your homepage.

We hope to receive your revised manuscript within two months. If you cannot send it within this time, please let us know. We will be happy to consider your revision so long as nothing similar has been accepted for publication at Nature Immunology or published elsewhere.

Please do not hesitate to contact me if you have any questions or would like to discuss these revisions further.

Nature Immunology is committed to improving transparency in authorship. As part of our efforts in this direction, we are now requesting that all authors identified as 'corresponding author' on published papers create and link their Open Researcher and Contributor Identifier (ORCID) with their account on the Manuscript Tracking System (MTS), prior to acceptance. ORCID helps the scientific community achieve unambiguous attribution of all scholarly contributions. You can create and link your ORCID from the home page of the MTS by clicking on 'Modify my Springer Nature account'. For more information please visit please visit www.springernature.com/orcid.

We look forward to seeing the revised manuscript and thank you for the opportunity to review your work.

Kind regards,

Laurie

Laurie A. Dempsey, Ph.D.
Senior Editor
Nature Immunology
l.dempsey@us.nature.com
ORCID: 0000-0002-3304-796X

Referee expertise:

Referee #1:

Referee #2:

Referee #3:

Reviewers' Comments:

Version 1:

Reviewer comments:

Reviewer #1

(Remarks to the Author)
This paper evaluates pregnancy induced protection of autoantibodies, and specifically focuses differences on the targets of autoantibodies between term and preterm pregnancies. This was accomplished analyzing 2700 sera samples from pregnant women using PhIP-seq. This analysis showed distinct term pregnancy and preterm birth antibody signatures. These results were somewhat verified using by mass spec evaluation of immunoprecipitated proteins from placental extracts for a limited subset of sera from term (n=13) and preterm (n=25) pregnancies showing 65% of the preterm birth signature proteins identified using PhIP-seq were also identified by mass spec. One of these targets for preterm birth autoantibodies in IL-1RA, and more focused analysis shows higher levels of antibodies targeting this protein in perterm pregnancies. The paper concludes with data showing pregnant mice treated with anti-IL-1RA antibodies have increased fetal resorption and other pregnancy defects.

I participated in the review for several prior versions of this manuscript for another journal. A consistent suggestion on each of these reviews was to improve the clarity, accessibility and accuracy of the writing (proofreading). Many parts of the text still contain specific technical details without explanation which makes it very hard to understand. Another consistent theme was that while evaluating the sera from >2700 pregnant women is commendable, the number of samples analyzed with preterm birth, and the rationale for how samples from term and preterm birth were chosen for more focused downstream analysis was not clearly described. I can see in the response to reviewers comments that the authors made some superficial changes (eliminating the prior inappropriate references from the abstract; adding sample size to the abstract). Unfortunately however these general concerns remain in this revised manuscript. While some data presented in the paper could be very interesting and helpful, the overall package is still very hard to understand, and as written probably more appropriate for a more specialized journal.

Specific comments-suggestions.
1. Line 133. What is the difference between a cross-sectional and population-based cohort design? Were the samples from males and control women that have never been pregnant from cohort 1 (the population based study?). Line 138, would evaluating sera from nulliparous women be a better control instead of nulligravid?
2. Lines 144 and 145. When adding all the samples in Fig 1a, one gets 3101. Please explain the source of the 1411 (4512-3101) samples? Are these all from the 130 healthy non-pregnant individuals? If so this seems like a lot, and it may be helpful to explain why so many unique samples were obtained from these non-pregnant controls.
3. Line 149. I am not it is obvious what anti-GFAP antibodies are? I presume this is antibodies targeting Glial Fibrillary Acidic protein? This seems like a very obscure self antigen. Why were antibodies against the particular self-antigen chosen? Some background, rationale and explanation or even writing out this uncommonly used abbreviation would be helpful.
4. Line 150. “(n=88)” cohorts. Is this a mistake since Fig 1A shows 8 pregnancy cohorts? If not, please explain how 8 pregnancy cohorts (plus samples from two non-pregnant [line 138] became 88.
5. Lines 156-158. The 4512 samples described in line 158 is now reduced to 3101. Is having “under-sequenced samples” in over 30% of the samples expected? reasonable? Without more explanation on this point, I worry that the analysis on auto-antibodies is already biased on ~70% samples with autoantibodies to begin with. Are the “under-sequenced samples” more enriched for samples from preterm birth?
6. Lines 164-165. Why was this cohort chosen for initial analysis? My concern is that this choosing embryo transfer pregnancies will almost certainly not represent normal pregnancy. Did these women have embryo transfer pregnancy due to some underlying disorder? If so, would this bias the interpretation of results evaluating auto-antibodies?
7. Line 167. More information is needed for evaluating how greater than or equal to 10 pregnancy samples was chosen. Cohort VI contains 13 pregnancies and 97 samples based on Fig 1a. How many of these pregnancies and how many of these 97 samples were included? For the criteria of “greater than or equal to 10 pregnancy samples” does it matter if the samples were primarily from the same person? Would it be helpful to expand the criteria to specifically indicate a minimum number of separate pregnant individuals are needed for defining a “pregnancy-specific peptide”. Here a distribution of data visually showing z-score > 3 would also be really helpful.
8. Line 171-172. I am not sure that most readers of Nature immunology will understand what “mean AUC of the receiver-operating-characteristic analysis” means. I think the whole paper would read a lot better if these complex words are explain or at least used in context. Without it, I worry this is essentially a meaningless sentence and piece of data.
9. Line 184. I am not sure that most readers of Nature immunology will understand what “mean RPK values at the peptide level” means. See additional comments for preceding point 8.
10. Line 187. Here preterm associated peptide are defined based on the presence in a minimum number of subjects (but previously it was based on number of samples without consideration for how many subjects?) See preceding point 7. “At least four preterm subjects” out of how many?
11. Lines 207 to 208. How were these peptides chosen? I assume this refers to peptides among the 9209 preterm-specific peptides described in lines 190-191? Where these the top 10 and top 500? If so, this should be clearly stated, and how differences in preterm peptide priority is assigned clear explained (is this based on difference magnitude between term

pregnancies?, P value?)
12. Figures 2. I think another very important consideration missing in these analysis is consideration of preterm birth not as binary variable, but rather a continuous variable.
13. Line 224. Is “severe” preterm birth the same as “early (Fig 3b)” preterm birth?
14. Line 232-235. Differences between spontaneous vs iatrogenic preterm birth is specifically called out as being “modestly higher” compared with the order comparisons (C-section, infant sex, history of preterm birth, preeclampsia), yet the P value for this is the highest (least statistically significant, P=0.6 [Extended data figure 6]). Please explain.
15. Line 243. Please show when the gestational age for when these samples were collected (similar to the data in Fig 1b), but just for this smaller subset.
16. Line 302. I could not find IL1R2 in Fig 6a. Is this a typo?
17. Line 304-305. Please clearly state which specific proteins that are “essential” and expression on the placenta refers to, with corresponding references.
18. Line 319. I am not sure reference 13 alone from 20 years ago showing intraamniotic injection of purified IL-1 in nonhuman primates means that the role for this cytokine in pregnancy is “well-established”. This is specifically in contrast to other parts of the paper (line 103) where “Little is known” used to describe the causes of preterm labor where I would argue just as much is known vs unknown.
19. Figure 6c and Figure 6d. Please clearly state when the samples were collected for each of the term and preterm birth samples, and whether these preterm births were early/severe, moderate or late.
20. Lines 342-343. Are the antibodies equally effective at pickup on different versions of this peptide based on differential glycosylation? If not, is it possible that there are differences in antibodies to IL-1RA specifically reflect one (glycosylated) variant of this glycoprotein? This should be specially evaluated.
21. Line 356. Which specific samples (from which cohorts, and levels of anti-IL-1RA and based which assay showing anti-IL-1RA activity) is this referring. Figure 6f would be a lot more meaningful if preterm samples with and without anti-IL1RA were directly compared, and samples from term pregnancies with and without anti-IL1RA were directly compared.
22. Lines 381, 384, 387. Is this referring to figure 7 rather than figure 3?
23. Extended data Figure 7. Would it be helpful to also analyze the data in Extended data Figure 6 using the same parameters (comparing with term pregnancy?)
23. Extended data Figure 8F (right sided). Percent primigravida and percent nulliparous in this context comparing the samples from pregnant women seem like the same thing? Should nulliparous be "multiparous"? or is this referring to the state of these women prior to the current pregnancy?

Reviewer #2

(Remarks to the Author)
The paper by Rackaityte et al. studies the presence of maternally transferred autoantibodies and it’s association with preterm birth.

The authors perform a proteome wide screening using PhiPSeq on several thousand pregnant women.

Interestingly, they identify autoantibodies to IL1RA (a negative regulator of IL1b) as enriched in preterm pregnancies. They convincingly show the impact of IL1RA in mice and its colocalization to endothelial adherens in human placenta.

Comments:
- Discussion about immunosuppressive therapies in women with recurrent preterm pregnancies would be interested
- Have the authors considered studying still births, which would be seen as an extreme phenotype
- The identification of Abs against HLA is interesting, but not much discussed. Do the authors have the HLA of the babies who were born to such mothers. Indeed, a matching of HLA antibodies targeting an HLA of the fetus would be very interesting
- A major caveat of PhiPSeq is that it can only study peptides, and not confirmational proteins. The authors should discuss this important issue.
- Neutralization shown in figure 6f is far from convincing, although this should be the most important confirmation that these autoantibodies to IL1RA are functional. I would suggest optimizing the technique to clearly show the neutralizing patients or consider using other assays.
- What is the impact of IL1RA AABs on systemic inflammation in these children and their mothers?
- Did any of the positive mothers have a particular clinical phenotype or disease ? Same question for the children.
- The prevalence of anti-IL1RA autoantibodies need to be clarified among the different groups, given the small number of patients. i.e. how many positive individuals within the preterm versus term babies.

Decision Letter:

5th Jul 2026

Dear Joe,

Thank you for providing a point-by-point response to the referees' comments on your manuscript entitled, "Evidence for Maternal Autoantibodies in the Pathogenesis of Preterm Birth".

As noted previously, I think many of the points raised by referee #1 (who has seen multiple versions of the study), is mostly

asking for clarifications in the descriptions of the cohorts, the results obtained in the study and the analysis methods & terminology. Referee #2 likewise is asking for a bit more context and discussion. I think your responses in your point-by-point response largely address those concerns.

However, for referee #1 point 4, regarding the sample number for the experiment in Fig. 1a, the reported "n" should be the number of independent experimental samples, not the number of times that a commercial polyclonal was used as a positive control. Are there 88 independent sera samples from the patient cohorts that are being assayed in this experiment? This point needed to be clarified.

Similarly, we encourage you to include the experimental data to address referee #2's point for the sera neutralization of IL1RA and clarifications addressing that concern.

When you revise your manuscript, please take into account all reviewer and editor comments, please highlight all changes in the manuscript text file in Microsoft Word format.

We therefore invite you to revise your manuscript taking into account all reviewer and editor comments. Please highlight all changes in the manuscript text file in Microsoft Word format.

We are committed to providing a fair and constructive peer-review process. Do not hesitate to contact us if there are specific requests from the reviewers that you believe are technically impossible or unlikely to yield a meaningful outcome.

When revising your manuscript:

* Include a “Response to referees” document detailing, point-by-point, how you addressed each referee comment. If no action was taken to address a point, you must provide a compelling argument. This response will be sent back to the referees along with the revised manuscript.

* If you have not done so already please begin to revise your manuscript so that it conforms to our Article format instructions at http://www.nature.com/ni/authors/index.html. Refer also to any guidelines provided in this letter.

* Please include a revised version of any required reporting checklist. It will be available to referees to aid in their evaluation of the manuscript goes back for peer review. They are available here:

Reporting summary:
https://www.nature.com/documents/nr-reporting-summary.pdf

When submitting the revised version of your manuscript, please pay close attention to our href="https://www.nature.com/nature-portfolio/editorial-policies/image-integrity">Digital Image Integrity Guidelines. and to the following points below:

-- that unprocessed scans are clearly labelled and match the gels and western blots presented in figures.
-- that control panels for gels and western blots are appropriately described as loading on sample processing controls
-- all images in the paper are checked for duplication of panels and for splicing of gel lanes.

Please note, Extended Data figures and tables are online-only (appearing in the online PDF and full-text HTML version of the paper), peer-reviewed display items that provide essential background to the Article but are not included in the printed version of the paper due to space constraints or being of interest only to a few specialists. A maximum of ten Extended Data display items (figures and tables) is typically permitted. When re-submitting your manuscript, please ensure that any supplementary figures and tables that are more critical to the manuscript’s conclusions are converted to Extended data to increase these data’s visibility.

Finally, please ensure that you retain unprocessed data and metadata files after publication, ideally archiving data in perpetuity, as these may be requested during the peer review and production process or after publication if any issues arise.

Please use the link below to submit your revised manuscript and related files:

Link Redacted

Note: This URL links to your confidential home page and associated information about manuscripts you may have submitted, or that you are reviewing for us. If you wish to forward this email to co-authors, please delete the link to your homepage.

We hope to receive your revised manuscript within 4 weeks. If you cannot send it within this time, please let us know. We will be happy to consider your revision so long as nothing similar has been accepted for publication at Nature Immunology or published elsewhere.

Please do not hesitate to contact me if you have any questions or would like to discuss these revisions further.

Nature Immunology is committed to improving transparency in authorship. As part of our efforts in this direction, we are now requesting that all authors identified as ‘corresponding author’ on published papers create and link their Open Researcher and Contributor Identifier (ORCID) with their account on the Manuscript Tracking System (MTS), prior to acceptance. ORCID helps the scientific community achieve unambiguous attribution of all scholarly contributions. You can create and link your ORCID from the home page of the MTS by clicking on ‘Modify my Springer Nature account’. For more information please visit please visit www.springernature.com/orcid.

We look forward to seeing the revised manuscript and thank you for the opportunity to review your work.

Kind regards,

Laurie

Laurie A. Dempsey, Ph.D.
Senior Editor
Nature Immunology
l.dempsey@us.nature.com
ORCID: 0000-0002-3304-796X

Reviewers' Comments:

Reviewer #1 (Remarks to the Author):

This paper evaluates pregnancy induced protection of autoantibodies, and specifically focuses differences on the targets of autoantibodies between term and preterm pregnancies. This was accomplished analyzing 2700 sera samples from pregnant women using PhIP-seq. This analysis showed distinct term pregnancy and preterm birth antibody signatures. These results were somewhat verified using by mass spec evaluation of immunoprecipitated proteins from placental extracts for a limited subset of sera from term (n=13) and preterm (n=25) pregnancies showing 65% of the preterm birth signature proteins identified using PhIP-seq were also identified by mass spec. One of these targets for preterm birth autoantibodies in IL-1RA, and more focused analysis shows higher levels of antibodies targeting this protein in perterm pregnancies. The paper concludes with data showing pregnant mice treated with anti-IL-1RA antibodies have increased fetal resorption and other pregnancy defects.

I participated in the review for several prior versions of this manuscript for another journal. A consistent suggestion on each of these reviews was to improve the clarity, accessibility and accuracy of the writing (proofreading). Many parts of the text still contain specific technical details without explanation which makes it very hard to understand. Another consistent theme was that while evaluating the sera from >2700 pregnant women is commendable, the number of samples analyzed with preterm birth, and the rationale for how samples from term and preterm birth were chosen for more focused downstream analysis was not clearly described. I can see in the response to reviewers comments that the authors made some superficial changes (eliminating the prior inappropriate references from the abstract; adding sample size to the abstract). Unfortunately however these general concerns remain in this revised manuscript. While some data presented in the paper could be very interesting and helpful, the overall package is still very hard to understand, and as written probably more appropriate for a more specialized journal.

Specific comments-suggestions.
1. Line 133. What is the difference between a cross-sectional and population-based cohort design? Were the samples from males and control women that have never been pregnant from cohort 1 (the population based study?). Line 138, would evaluating sera from nulliparous women be a better control instead of nulligravid?
2. Lines 144 and 145. When adding all the samples in Fig 1a, one gets 3101. Please explain the source of the 1411 (4512-3101) samples? Are these all from the 130 healthy non-pregnant individuals? If so this seems like a lot, and it may be helpful to explain why so many unique samples were obtained from these non-pregnant controls.
3. Line 149. I am not it is obvious what anti-GFAP antibodies are? I presume this is antibodies targeting Glial Fibrillary Acidic protein? This seems like a very obscure self antigen. Why were antibodies against the particular self-antigen chosen? Some background, rationale and explanation or even writing out this uncommonly used abbreviation would be helpful.
4. Line 150. “(n=88)” cohorts. Is this a mistake since Fig 1A shows 8 pregnancy cohorts? If not, please explain how 8 pregnancy cohorts (plus samples from two non-pregnant [line 138] became 88.
5. Lines 156-158. The 4512 samples described in line 158 is now reduced to 3101. Is having “under-sequenced samples” in over 30% of the samples expected? reasonable? Without more explanation on this point, I worry that the analysis on auto-antibodies is already biased on ~70% samples with autoantibodies to begin with. Are the “under-sequenced samples” more enriched for samples from preterm birth?
6. Lines 164-165. Why was this cohort chosen for initial analysis? My concern is that this choosing embryo transfer pregnancies will almost certainly not represent normal pregnancy. Did these women have embryo transfer pregnancy due to some underlying disorder? If so, would this bias the interpretation of results evaluating auto-antibodies?
7. Line 167. More information is needed for evaluating how greater than or equal to 10 pregnancy samples was chosen. Cohort VI contains 13 pregnancies and 97 samples based on Fig 1a. How many of these pregnancies and how many of

these 97 samples were included? For the criteria of “greater than or equal to 10 pregnancy samples” does it matter if the samples were primarily from the same person? Would it be helpful to expand the criteria to specifically indicate a minimum number of separate pregnant individuals are needed for defining a “pregnancy-specific peptide”. Here a distribution of data visually showing z-score > 3 would also be really helpful.
8. Line 171-172. I am not sure that most readers of Nature immunology will understand what “mean AUC of the receiver-operating-characteristic analysis” means. I think the whole paper would read a lot better if these complex words are explain or at least used in context. Without it, I worry this is essentially a meaningless sentence and piece of data.
9. Line 184. I am not sure that most readers of Nature immunology will understand what “mean RPK values at the peptide level” means. See additional comments for preceding point 8.
10. Line 187. Here preterm associated peptide are defined based on the presence in a minimum number of subjects (but previously it was based on number of samples without consideration for how many subjects?) See preceding point 7. “At least four preterm subjects” out of how many?
11. Lines 207 to 208. How were these peptides chosen? I assume this refers to peptides among the 9209 preterm-specific peptides described in lines 190-191? Where these the top 10 and top 500? If so, this should be clearly stated, and how differences in preterm peptide priority is assigned clear explained (is this based on difference magnitude between term pregnancies?, P value?)
12. Figures 2. I think another very important consideration missing in these analysis is consideration of preterm birth not as binary variable, but rather a continuous variable.
13. Line 224. Is “severe” preterm birth the same as “early (Fig 3b)” preterm birth?
14. Line 232-235. Differences between spontaneous vs iatrogenic preterm birth is specifically called out as being “modestly higher” compared with the order comparisons (C-section, infant sex, history of preterm birth, preeclampsia), yet the P value for this is the highest (least statistically significant, P=0.6 [Extended data figure 6]). Please explain.
15. Line 243. Please show when the gestational age for when these samples were collected (similar to the data in Fig 1b), but just for this smaller subset.
16. Line 302. I could not find IL1R2 in Fig 6a. Is this a typo?
17. Line 304-305. Please clearly state which specific proteins that are “essential” and expression on the placenta refers to, with corresponding references.
18. Line 319. I am not sure reference 13 alone from 20 years ago showing intraamniotic injection of purified IL-1 in nonhuman primates means that the role for this cytokine in pregnancy is “well-established”. This is specifically in contrast to other parts of the paper (line 103) where “Little is known” used to describe the causes of preterm labor where I would argue just as much is known vs unknown.
19. Figure 6c and Figure 6d. Please clearly state when the samples were collected for each of the term and preterm birth samples, and whether these preterm births were early/severe, moderate or late.
20. Lines 342-343. Are the antibodies equally effective at pickup on different versions of this peptide based on differential glycosylation? If not, is it possible that there are differences in antibodies to IL-1RA specifically reflect one (glycosylated) variant of this glycoprotein? This should be specially evaluated.
21. Line 356. Which specific samples (from which cohorts, and levels of anti-IL-1RA and based which assay showing anti-IL-1RA activity) is this referring. Figure 6f would be a lot more meaningful if preterm samples with and without anti-IL1RA were directly compared, and samples from term pregnancies with and without anti-IL1RA were directly compared.
22. Lines 381, 384, 387. Is this referring to figure 7 rather than figure 3?
23. Extended data Figure 7. Would it be helpful to also analyze the data in Extended data Figure 6 using the same parameters (comparing with term pregnancy?)
23. Extended data Figure 8F (right sided). Percent primigravida and percent nulliparous in this context comparing the samples from pregnant women seem like the same thing? Should nulliparous be "multiparous"? or is this referring to the state of these women prior to the current pregnancy?

Reviewer #2 (Remarks to the Author):

The paper by Rackaityte et al. studies the presence of maternally transferred autoantibodies and it’s association with preterm birth.

The authors perform a proteome wide screening using PhiPSeq on several thousand pregnant women.

Interestingly, they identify autoantibodies to IL1RA (a negative regulator of IL1b) as enriched in preterm pregnancies. They convincingly show the impact of IL1RA in mice and its colocalization to endothelial adherens in human placenta.

Comments:
- Discussion about immunosuppressive therapies in women with recurrent preterm pregnancies would be interested
- Have the authors considered studying still births, which would be seen as an extreme phenotype
- The identification of Abs against HLA is interesting, but not much discussed. Do the authors have the HLA of the babies who were born to such mothers. Indeed, a matching of HLA antibodies targeting an HLA of the fetus would be very interesting
- A major caveat of PhiPSeq is that it can only study peptides, and not confirmational proteins. The authors should discuss this important issue.
- Neutralization shown in figure 6f is far from convincing, although this should be the most important confirmation that these autoantibodies to IL1RA are functional. I would suggest optimizing the technique to clearly show the neutralizing patients or consider using other assays.
- What is the impact of IL1RA AABs on systemic inflammation in these children and their mothers?

- Did any of the positive mothers have a particular clinical phenotype or disease ? Same question for the children.
- The prevalence of anti-IL1RA autoantibodies need to be clarified among the different groups, given the small number of patients. i.e. how many positive individuals within the preterm versus term babies.

Version 2:

Decision Letter:

Our ref: NI-A43122B

24th Jul 2026

Dear Joe & Elze,

Thank you for submitting your revised manuscript "Evidence for Maternal Autoantibodies in the Pathogenesis of Preterm Birth" (NI-A43122B). We have looked over your clarifications and responses to the last round of review, and we are now satisfied that the story can move forward. Therefore we'll be happy in principle to publish it in Nature Immunology, pending minor revisions to comply with our editorial and formatting guidelines.

We will now perform detailed checks on your paper and will send you a checklist detailing our editorial and formatting requirements in a couple of weeks. Please do not upload the final materials and make any revisions until you receive this additional information from us.

If you had not uploaded a Word file for the current version of the manuscript, we will need one before beginning the editing process; please email that to immunology@us.nature.com at your earliest convenience.

Thank you again for your interest in Nature Immunology Please do not hesitate to contact me if you have any questions.

Kind regards,

Laurie

Laurie A. Dempsey, Ph.D.
Senior Editor
Nature Immunology
l.dempsey@us.nature.com
ORCID: 0000-0002-3304-796X

INTERNAL USE ONLY AIP modules required: ["Life Science"]

Open Access This Peer Review File is licensed under a Creative Commons Attribution 4.0 International License, which permits use, sharing, adaptation, distribution and reproduction in any medium or format, as long as you give appropriate credit to the original author(s) and the source, provide a link to the Creative Commons license, and indicate if changes were made.
In cases where reviewers are anonymous, credit should be given to 'Anonymous Referee' and the source.
The images or other third party material in this Peer Review File are included in the article's Creative Commons license, unless indicated otherwise in a credit line to the material. If material is not included in the article's Creative Commons license and your intended use is not permitted by statutory regulation or exceeds the permitted use, you will need to obtain permission directly from the copyright holder.
To view a copy of this license, visit https://creativecommons.org/licenses/by/4.0/

POINT BY POINT RESPONSE TO REVIEWERS

Referee #1

1. Fig 7b indicates that the preterm autoantibody signature is not significantly different comparing spontaneous and iatrogenic preterm birth. This is a surprising finding. The non-specificity of the relationship makes me concerned about the possibility of biases in the data explaining associations. The great diversity of the studies used in this analysis is a weakness.

Extended Figure 7b shows the summed autoantibody response within the preterm signature across all peptides. This combined signature was significantly higher in more severe preterm births (Ext Data 7a) and in all preterm compared to term (Fig 2a), though it did not reach significance for other clinical subtypes. We previously performed subgroup analysis *within* the combined preterm signature across various clinical groupings of preterm pregnancies. This is represented in Extended Data Fig 8.

In the revised text we now state:

> "While the overall preterm autoantibody signature did not differ significantly between spontaneous and iatrogenic preterm births (**Extended Data Fig 7b**), subtype-specific analyses within the signature revealed distinct enrichment profiles: for example, MSX1 was enriched in spontaneous versus iatrogenic preterm births, PKP1 in pregnancies without preeclampsia, and ING4 in high-gravidity mothers (**Extended Data Fig 8**), suggesting that the preterm autoantibody landscape reflects heterogeneous clinical etiologies."

The great diversity of studies is a strength of the study, because it brings together cohorts across sites, populations, and clinical contexts, demonstrating that the signal is reproducible and not driven by cohort-specific biases or confounders, but instead reflects a robust and generalizable biological phenomenon. We added four new cohorts over the course of our extensive revisions, at the request of the reviewer.

In their response to my first comment, they state (page 3, revised text) that they performed an "odds ratio analysis". I am not sure what they mean by this. An odds ratio is a proportion, it is not a statistical method. They have calculated odds ratios for different combinations of features and then compared them. They found that the combination of a history of previous preterm birth and a 1 SD increase in the autoantibody signature had an OR of 8.36 and this was "multiplicative", given the individual ORs were 5.28 and 1.80, respectively. The product of the two odds ratios was 9.5. Generally the term "multiplicative" is used to describe a positive interaction between two features and this would generally be used to describe a situation where the combination of two features was associated with an OR which was greater than the

product of the two individual ORs. Where the combination of two features is the product of the two odds ratios, this is generally referred to as additive. While this may sound counterintuitive, this refers to the beta coefficients from logistic regression (another way of expressing the model). Adding two coefficients is the same as multiplying two odds ratios. An appropriate analysis method here would be multivariate logistic regression, with preterm birth as the dependent variable, previous preterm birth as a binary independent variable and autoantibody signature as a continuous independent variable. A positive interaction would be tested by creating an interaction term between the two independent variables (their product) and testing its significance using a log rank test. I suggest they call the association additive if there is no statistically significant interaction between these two variables.

We have changed the text to additive. We have changed "odds ratio analysis" to logistic regression analysis.

The text has been revised as follows:

> "To determine whether the preterm autoantibody signature is associated with preterm birth risk relative to established obstetric risk factors, a logistic regression analysis was performed was performed (**Fig 2b**)."
>
> "Notably, the autoantibody signature and clinical history provided largely independent risk information: in a multivariate model, patients with both prior preterm birth and elevated autoantibody burden (+1 SD) exhibited substantially increased odds (OR = 8.36, 95% CI 2.97–23.52) compared to those with neither risk factor, consistent with additive effects on the odds scale."

2. I requested that they add 95% CI and P values for the AUC. They have chosen not to add P values, which is unhelpful. I note that they express the 95% CI as plus or minus. This is relatively unusual. Typically, 95% CI are expressed as a range. CIs for the AUC are not generally symmetric. The AUC is bounded on [0, 1], and methods that respect those bounds (e.g. logit-transformed intervals or bootstrap percentile/BCa intervals) will typically produce asymmetric CIs, particularly as the estimate moves away from 0.5. Intuitively, if the AUC is close to 1, the interval cannot extend far in that direction, so some asymmetry is to be expected. Some commonly used approaches (e.g. those based on a normal approximation, as used in DeLong's method) do give symmetric intervals of the form estimate ± margin. However, that symmetry arises from the approximation rather than the true sampling distribution, and can lead to intervals extending beyond [0, 1] when the AUC is near the boundaries. 95% CI should be expressed as a range a P value added.

1. All AUC confidence intervals are now reported as asymmetric bootstrap percentile intervals in bracket form: e.g., AUC = 0.76 (95% CI 0.71–0.81). We use 2,000 bootstrap iterations with the 2.5th and 97.5th percentiles as bounds.

2. DeLong one-sample P-values (DeLong et al. 1988) vs. AUC = 0.5 have been added for all reported AUCs. The overall held-out test set AUC is 0.761 (95% CI 0.710–0.808, $P = 2.8 \times 10^{-25}$).

3. All figure legends, figures, and Supplementary Methods have been updated to reflect this format. No AUC is reported as ± margin in the revised manuscript.

We have also verified that the bootstrap percentile intervals respect the [0, 1] bounds of the AUC and produce appropriately asymmetric intervals, addressing the reviewer's concerns about symmetric approximation artifacts near the boundaries.

Figure legends and supplementary methods have been updated accordingly.

3. The authors acknowledge the problem of lacking true out of sample validation and it looks like this is a problem they are unable to address. I appreciate that they have de-emphasised the screening test element of their presentation of the data. But their inability to address this point does substantially reduce the interest in the work. The other reviewer has highlighted the diversity of methods of assessment of the antibody signature. I would strongly recommend this paper for publication if they identified the optimal method of assessment and applied it to the samples from a prospective cohort study and could demonstrate that a PRE-SPECIFIED definition of screen positive had clinically significant prediction out of sample. A cohort such as the NICHD study, NuMoM2b (https://pubmed.ncbi.nlm.nih.gov/25648779/), would be ideal.

The scope and the contribution of the present study open exciting opportunities to develop and validate precisely the screening approaches the reviewer describes. Our objective is not to present a finalized clinical screening test, but to define and validate a previously unrecognized, biologically coherent autoantibody signature associated with preterm birth across independent cohorts and orthogonal platforms. The absence of a prospective, pre-specified screening threshold in a cohort such as NuMoM2b does not diminish the central advance: the identification of a reproducible immunological phenomenon with mechanistic and predictive relevance.

Importantly, the signal is validated across multiple independent cohorts, sampling schemes, and analytical modalities. Prospective clinical validation is a natural and necessary next step, but it is not a prerequisite for establishing biological discovery and translational potential, as widely recognized in biomarker development frameworks.

Thus, rather than reducing impact, the current work establishes a strong and generalizable foundation that enables precisely the type of prospective validation the reviewer proposes.

Referee #2 (Remarks to the Author):

The authors have addressed suggestions on the original and revised manuscript, and each revision is progressively improved from both a clarity perspective on which samples are used, and sample size considerations. Since this type of publication will likely draw a lot of attention, I have a few additional suggestions for clarity and accuracy.

1. Opening paragraph, sentence 2 (line 62). I am not sure any of the provided references specifically provide primary data describing spontaneous preterm labor as the most common cause of preterm delivery. I wonder if citing something like the ACOG practice bulletin (PMID 34293771) would be appropriate? Since the provided (Extended data Figure 7B) do not any difference between spontaneous vs iatrogenic preterm birth, I also wonder if this emphasis on spontaneous preterm birth early on is the intended focus?

We have removed focus on spontatenous preterm birth in the abstract. Citations are not recommended in the abstract and we have removed them.

2. Opening paragraph, sentence 3 (line 63). Many of these references are not dealing with “autoantibodies”, but rather maternal antibodies targeted against paternal-fetal expressed antigens. For example, reference 6 is not referring to Rh as a “autoantibody” since Rh haemolytic disease would be occurring in Rh-negative mothers and not considered an autoantibody. Please double check all these references to ensure its autoantibodies, rather than antibodies against paternal antigens expressed by the fetus.

We have clarified in the text that there is precedent for both allo- and autoantibodies affecting pregnancy outcomes. We also clarify that all current proteomic assays, including ours, cannot distinguish between allo- and autoreactivities.

3. Opening paragraph, sentence 4 (line 65). Since the introduction and prior sentences are focused on preterm birth, would it be helpful to highlight the preterm birth sample size, rather than the sample size of all pregnant individuals? If emphasis on spontaneous preterm birth, can highlight that sample size, rather than all pregnancies?

We have removed emphasis on spontaneous preterm birth. We have added the number preterm births into the abstract.

4. Opening paragraph, sentences 5 and 6 (lines 68 and 70). These sentences are confusing because what preterm birth is being compared to is not clearly defined. Is it the antibodies from women with term pregnancy or non-pregnant individuals?

We have clarified that it is compared to term pregnancy.

5. Opening paragraph, sentence 9 (line 76). I could not find in the text, figures or supplementary table where this 6% value came from. It may be helpful to also clearly state what percent of sera from women with term pregnancy and non-pregnant individuals have antibodies against IL1RA? This may help the reader understand how to better interpret what 6% means in this context.

6% prevalence is clearly stated in Line 310 (now line 320) of the results and represented in Figure 3c.

We have modified the abstract to include the prevalence in term pregnancies.

6. These types of very granular suggestions really can be applied throughout the entire text. If helpful, it may be useful to carefully go through the text line by line to ensure the focus and references are as intended.

We have performed a thorough line-by-line review of the manuscript to ensure consistency in the following areas:

- All comparisons specify the comparator group (term vs preterm, non-pregnant vs pregnant)
- References are correctly categorized as addressing autoantibodies vs alloantibodies
- Sample sizes include both numerator and denominator where prevalence is stated
- Figure legends match the statistical methods described in Supplementary Methods
- AUC values use the corrected asymmetric CI + P-value format throughout

PREVIOUS RESPONSE TO REVIEWERS:

We thank the referees for their detailed and constructive comments, which guided a further revision of the manuscript.

Referee 1:

The authors have considerably expanded the number of human samples analysed and have performed a substantial amount of additional work which has addressed my main concerns. I have only one significant persisting concern and a few minor points.

The weakest element of the paper is the description of the screening test, and my concerns are described below.

1. They are predicting all preterm birth. Preterm birth is the end point of multiple different pathological processes (PMID: 25124429). It is not plausible that a single test will be predictive of all the subtypes. Even if they were all associated with the development of maternal autoantibodies, they would likely be different antibodies. I would anticipate that maternal autoantibodies might be more strongly associated with some of the subtypes over others, and this should be identified as an area of further study.

We agree that is unreasonable to capture all possible causes and subtypes of preterm birth within PhIP-seq profiles, and we never meant to suggest otherwise. We have changed this section to directly reflect this point while making it clear that the logistic regression analysis was designed to assess the extent to which preterm birth may be detected through these profiles.

We performed additional analyses to examine the heterogeneity of model performance (**Extended Data Fig 9**). Predictive ability was evaluated in the hold-out validation set after stratification by available clinical metadata. The model performed best for spontaneous preterm birth (AUC 0.82) and showed strongest discrimination for very early preterm births (AUC 0.83 for very early PTB versus 0.73 for late PTB). Gestational age at sampling did not significantly influence predictive ability, and very early preterm births were better predicted than late preterm births even when restricting analysis to samples collected before 16 weeks of gestation. Prediction was most robust among

primigravid pregnancies (AUC 0.73), whereas term pregnancies predicted by the model as high-probability preterm births were primarily multigravidae, suggesting that prior pregnancy history may influence the immunological signature detected by the model. Notably, the ability to identify pregnancies at risk for very early preterm birth is particularly important, as these infants experience the highest rates of neonatal morbidity and mortality. Spontaneous preterm birth also remains extremely difficult to predict clinically, particularly in the first trimester, and prediction in primigravid pregnancies is especially challenging given that the strongest established risk factor for preterm birth is a prior preterm delivery.

The modified paragraph introducing the regression analysis now reads:

> While it is unrealistic that autoreactivity enrichment would be sufficient to capture all causes of preterm birth, a simple logistic regression model was constructed to determine what predictive value may be contained within PhIP-seq profiles. Data from eight independent cohorts were partitioned into sets for feature selection and model training (n = 2,607), with representative samples from each cohort held out for independent validation (n = 466; **Extended Data Fig 9a–c**; Supplementary Methods). The final trained model was utilized to predict preterm or term delivery outcomes of the hold-out set, which was never used for training or feature selection. As expected, model performance on the validation cohorts was less than the training cohorts, however, forecasting ability was still achieved (ROC AUC 0.76 ±0.026; **Fig 2c-d**; **Supplementary Table 4**; Supplementary Methods). Discrimination was stable across trimesters of sample collection, including samples obtained during the first trimester (AUC 0.71; **Extended Data Fig 9d**), was highest among primigravidae (AUC 0.73; **Extended Data Fig 9e**), and predicted spontaneous preterm birth subtype best (AUC 0.82; **Extended Data Fig 9f**). Predicted risk scores exhibited a monotonic severity gradient, with higher scores associated with earlier gestational age at delivery (AUC 0.83 for very early PTB versus 0.73 for late PTB; **Extended Data 9h-i**), and this remained significant even after restricting to samples collected before 16 weeks of gestation (AUC 0.83 for very early PTB; **Extended Data 9j**). Notably, the highest-risk term samples were primarily multiparous women, suggesting that prior pregnancy history may influence the immunological signature (**Extended Data 9i**). Analysis of model performance suggests that the

immunological changes associated with preterm birth are heterogeneous and confirm the necessity for numerous and diverse cohorts for training.

With regards to subtypes, we previously performed a stratification by iatrogenic versus spontaneous preterm delivery subtypes and found that no difference in the overall autoantibody profile (Extended Data Fig 7b). New to this revision, we have performed a KS-test across the clinical metadata variables available to identify autoreactivities specific to each clinical subtype (rather than the global signature). This is represented in a new **Extended Data Fig 8**, where we demonstrate the enrichment of autoreactivity stratified by spontaneous preterm birth, iatrogenic preterm birth, history of preterm birth, preeclampsia diagnosis, severity of preterm delivery (gestational age at delivery), and gravidity.

The modified paragraph describing this analysis now reads:

> Within-preterm comparisons revealed subtype-specific autoantibody associations, with distinct enrichment profiles distinguishing clinical subtypes (Supplementary Methods; **Extended Data Fig 8**). For example, MSX1 was enriched in spontaneous versus iatrogenic preterm births, PKP1 in pregnancies without preeclampsia, and ING4 in high-gravidity mothers (**Extended Data Fig 8**), suggesting that the preterm autoantibody landscape reflects heterogeneous clinical etiologies.

The reviewer's point indirectly touches on whether the autoantibody signature contributes independent information relative to conventional clinical risk factors, such as history of preterm birth. Along with the revision of Fig 2c and Fig 2d (the logistic regression AUCs), we have expanded the odds ratio analysis in Fig 2b, to include a multivariate analysis which assesses the odds ratio when the patients with elevated autoantibody signature (at the +1 SD, and +2 SD level) is combined with history of preterm birth (the best obstetric predictor of subsequent preterm delivery). Here, the combined measures achieve significantly increased odds ratios, suggesting independence between the clinical history and the autoantibody signature.

The text associated with the odds ratio analysis now reads:

> To determine whether the preterm autoantibody signature is associated with preterm birth risk relative to established obstetric risk factors, an odds ratio analysis was performed (**Fig 2b**). Each standard deviation increase in the summed log-fold change over mock IP was associated with significantly higher odds of preterm delivery (all features: OR = 1.54, 95% CI 1.28–1.85). To assess the strength of the association as a function of the number of autoantibody signature features, the feature set was restricted from as few as 10 peptides to as many as 500 peptides. Even with only 10 peptide features, the odds ratios were still significant (OR = 1.32, 95% CI 1.11–1.56), and by 100 features, the maximum odds ratio was achieved (OR = 1.80, 95% CI 1.49–2.17). Established obstetric risk factors showed the expected direction and magnitude: prior preterm birth (OR = 5.28, 95% CI 1.95–14.28) and preeclampsia (OR = 4.52, 95% CI 1.78–11.52) were each associated with increased risk. In contrast, maternal age (OR = 0.89, 95% CI 0.75–1.05) and gravidity (OR = 1.22, 95% CI 1.01–1.46) per standard deviation were not strongly associated with preterm birth risk. Notably, the autoantibody signature and clinical history provided largely independent risk information: in a multivariate model, patients with both prior preterm birth and elevated autoantibody burden (+1 SD) exhibited substantially increased odds (OR = 8.36, 95% CI 2.97–23.52) compared to those with neither risk factor, consistent with multiplicative effects on the odds scale.

2. They report an out of sample AUC of 0.75. I would not regard this level of AUC as indicating “strong forecasting ability” (line 264), I would call this moderately predictive. But, importantly, there is no 95% CI or P value for the AUC. They need to add the confidence interval and P value both to the text (line 265) and Figure (2c).

We report the confidence interval in the revised figure 2.

The modified paragraph describing this analysis now reads:

> As expected, model performance on the validation cohorts was less than the training cohorts, however, forecasting ability was still achieved (ROC

AUC 0.76 ±0.026; **Fig 2c-d**; **Supplementary Table 4**; Supplementary Methods).

3. For me, the external validation of the screening performance remains methodologically weak. The best discovery/validation experiment performs discovery in one group of patients. It then generates a predictive model, locks it down, and tests its discrimination and calibration in a separate validation cohort, ideally, one that is demographically and geographically distinct from the development cohort. Instead, these authors have pooled all eight cohorts together and used a hold-out sample for external validation, including participants from all eight cohorts, for external validation. Lines 265-267 comment that the model performance indicates that the maternal autoantibody signatures associated with preterm birth are varied. I agree and the conclusion is that it might be very difficult to create a clinically useful screening test using this method. By developing and validating the models in the same studies it is very likely that even the AUC of 0.75 is overestimated compared with what would be achieved in a true external validation.

I think the science behind this paper is strong and I think the data are true and important. But, personally, I would de-emphasise the screening element of the analysis.

We agree that leave-one-cohort-out (LOCO) analysis is a gold standard for testing model generalizability. When performing LOCO, it is important to consider confounders when selecting the final test cohort. Cohorts I, II, and V are balanced case-control cohorts, however they minimally overlap in gestational age at time of sampling. Cohorts II, IV, VI, and VII have significant class imbalance for use as a true hold-out cohort (**Extended Data Fig 1**). While Cohort VIII has significant class imbalance, it does have enough preterm cases to estimate performance and there is sufficient overlap in gestational age at the time of sampling with other cohorts. However, the median gestational age at preterm delivery is 36 weeks and our previous analyses indicate that the signature is strongest in earlier preterm deliveries (**Extended Data Fig 7a**). Due to the influence of sampling time during gestation on the autoantibody signature, we did not have sufficient training samples at every gestational age to power leave-one-cohort-out analysis.

Therefore, we have de-emphasized the predictor in the following ways:

- Removed reference to a predictor from the abstract
- Condensed the paragraph describing the predictor model.
- Moved predictor model development methods to supplementary methods.
- Removed reference to a diagnostic from the abstract
- Condensed and revised the paragraph describing the logistic regression model (see above)
- Moved logistic regression model development methods to Supplementary Methods.

Other points:
1. Line 126: “nulliparous” should be “nulligravid”. Nulliparous women can be pregnant – between conception and birth.

Corrected.

2. Lines 208-209. “…whose T labor promoting effects…” Please explain.

We apologize for the typo. It is corrected.
3. Line 330. Extended Fig 11d indicates a 100% probability of preterm birth with the presence of IL-1RA antibodies between 15 to ~28 weeks. But Fig 11c demonstrates that some women having term births have IL-1RA antibodies within that gestational age window. Panel d is from the fitted logistic regression model and the panel c indicates that the model does not fit the data particularly well. I don’t think panel d adds much and I would drop it.

We agree and have removed this analysis.
4. Line 372. Typo. Should be Il not il.

Corrected.
5. Lines 530-531. Did they dissect off maternal decidua from their placental biopsies?

We have clarified in the methods that biopsies sampled anchoring villi, which may include a small amount of decidua.

> Biopsies were taken perpendicular to the basal surface to ensure sampling of anchoring villi, and may include a small amount of decidua; 1 cm^3 blocks were flash frozen in liquid nitrogen.

Referee 2:
The revised paper is improved, but many areas remain unclear. While the sample size

is increased for PhiP-seq and there is some additional new analysis provided, many inconsistencies and inadequately explained methodology remain. As with the original version, there are some potentially interesting findings, but the take home message of the paper (maternal antibodies in the pathogenesis of preterm birth) not well flushed out. The writing also remains very technical, and in many instances the figures and legends are lacking in clarity, that I worry will be hard to follow and understand for a broad scientific audience. Specific comments.

1. Preterm birth is associated with antibodies against more self-placental proteins, and one of these self proteins is IL-1RA. There is considerable focus on anti-IL-RA autoantibodies. However I am still not sure what proportion of preterm birth was associated with these auto-antibodies because the data is presented using different assays for different cohorts without clear justification for why samples from one cohort were selected over another, and why one assay was selected over another for any one cohort (lines 314-323). The justification for in-depth study of anti-IL-1RA would be more clear if samples from all preterm birth samples were tested using a standard assay. In this case using PhIPseq, the prevalence of these autoantibodies is 0.3% (lines 311-312). Why is it increased by over 10-fold or more in selected cohorts using specific assays? Without a clear justification for why samples from one cohort were selected over another, and why one assay was selected over another for any one cohort, the link and impact for subsequent analysis for anti-IL-1RA studies is incomplete.

We have validated the presence of anti-IL1RA antibodies in preterm sera using five orthogonal methods (PhIP-seq, IP-MS, Luminex assay, *in vitro* cell-based reporter assay, and blocking of placental target by immunofluorescence). This strongly supports the conclusion that functionally neutralizing anti-IL1RA antibodies are present.

Each antibody detection approach varies in the amount of plasma, complexity of the experiment, and cost. Therefore, it is not feasible to test every sample with every orthogonal approach, especially given limited human sample volumes. We have tested every sample by PhIP-seq, which represents the largest autoantibody profiling effort ever attempted in pregnancy. Regardless, with the remaining available sample, we have expanded the number of samples analyzed for functional IL1β neutralization (see response to Point 4 below).

Since we have used a variety of different methods across the sample cohorts, we have compiled a new **Supplementary Table 6** summarizing all samples that were assessed

for anti-IL1RA positivity by at least two orthogonal methods, in one concise table. We have additionally updated **Supplementary Table 1**; **tab 3** to reflect which samples were chosen for each orthogonal technique.

We acknowledge that detection varies by method of measurement. This is expected and has been reported for other anti-cytokine antibodies (see Wang et al 2022 Cell Reports Methods Supplementary Figure 1D). We have added this caveat in the discussion, we now state:

> The prevalence of anti-IL1RA antibodies varied across orthogonal detection methods (PhIP-seq, IP-MS, Luminex, cell-based reporter assay, and immunofluorescence blocking), ranging from 0.4% by PhIP-seq to 16% by IP-MS. Such assay-dependent variation in prevalence has been reported for anti-cytokine antibodies[1,2] and likely reflects differences in sensitivity, epitope accessibility, and the distinction between linear and conformational epitope detection.

2. Figure 3a-c are really hard to understand. Panel a is labelled “0.3% positive”, corresponding to text line 311. I presume this is 3/802 which equals 0.374%? Panel a seems to also show more data points with anti-IL-1RA in term pregnancies, but with lower “sum fold-change”. What is the percent positive in these term pregnancies? What was the cut-off in “sum fold-change” used in these calculations and how was this determined?

We apologize for the rounding error and have corrected the percentage to 0.4% positivity.

To define seropositivity, we used a highly conservative outlier threshold relative to the distribution observed in term pregnancies. Specifically, samples were considered positive when their summed fold-change exceeded the mean of term samples by greater than 50 standard deviations. This stringent threshold was selected to ensure that only extreme outliers were classified as positive and to minimize false positives arising from the heavy-tailed distribution typical of PhIP-seq measurements. Using this cutoff, no term pregnancies met the positivity criterion. This definition of seropositivity has now been clarified in the figure legend.

Panel b is labelled “16% positive” using IP-MS (4/25). The corresponding text (lines 316 -317) state antibody recovery in 20% of the samples??? Is there a data point missing? What were only such a small number (n=13) of term pregnancy samples evaluated using IP-MS?

We apologize for the typo on lines 316, it has now been corrected to 16% positivity. As stated in the methods, term samples were randomly selected for inclusion in the IP-MS experiment.

In our previous correspondence we stated:

> “While our IP-MS experiments utilized state-of-the-art high-throughput methods and highly sensitive instrumentation, the approach remains time-consuming and expensive. Instead, we more than tripled the number of samples analyzed by PhIP-seq, a highly scalable and orthogonal technique.”

We have added an explanation for sample selection and the associated caveats into the discussion. We now state:

> Furthermore, sample selection for orthogonal validation was determined by sample availability and the number of anti-IL1RA-positive pregnancies identified across cohorts remains underpowered to assess associations with clinical metadata variables.

Panel c shows 5 above background samples in the preterm group, 5/65 = 7.69%. Is this how 7% was calculated? How about the one sample positive sample in the term pregnancy group? What do the dotted lines mean in panel c? This is neither explained in the legend nor in the corresponding text (lines 317-318).

We apologize for confusion and have revised the legend to explain how the prevalence was calculated. We have corrected the panel to state 6% which was the threshold for z=2 greater than mean in term (4/65).

3. In response to the prior comment about not considering preterm birth as a binary condition, and instead comparing early versus late preterm birth, there is some

additional analysis provided. However this is not followed upon in a meaningful way. For example, are anti-IL-1RA antibodies found more in early or late preterm birth?

In our previous revision, we did compare early versus late preterm birth outcomes for the entirety of the preterm autoantibody signature (**Extended Data Fig 7a**). In response to Reviewer 1, we have provided additional stratification (see above).

We have added **Supplementary Table 6** which describes the clinical features of anti-IL1RA positive pregnancies, as available in our metadata. The numbers remain too small for clinical correlate analysis. We are assembling large prospective cohort, outside the scope of this manuscript, that will be adequately powered to assess the clinical variable association with anti-IL1RA positivity. We have amended this to the discussion as described above.

4. Reproducibility in key data is lacking. For example figure 3e. Are these data points (three per group) from triplicate wells? If so, how many times was this experiment repeated? Are the 3 data points using serum from three different women with term and preterm birth? Among the thousands of serum samples available, how were the 3 individual term and 3 preterm samples chosen for this experiment?

Functional assays use considerable amounts of serum, which is limited for all cohorts tested. New to this revision, we developed a miniaturized protocol, which allowed us to add data from n=10 nulligravidae, n=19 term pregnant, and n=6 anti-IL1RA+ preterm pregnancy samples. Each specimen was tested in technical duplicate (**Figure 3e**). An 8-point dilution series for each specimen is represented in **Extended Data Fig 14d**. For one of the anti-IL1RA samples, we had sufficient serum to screen multiple IL1RA concentrations to determine the IC50 of the neutralizing antibody (**Extended Data Fig 14c**).

Samples were chosen by sample availability, a caveat we now add into the discussion (see response to point 2).

Serum functional assays for anti-IL1RA inhibition have been reported for other diseases and our numbers are within the range of previous studies:

- Pfeifer et al 2022 Lancet tested n=2 plasma samples[3]
- Thurner et al 2022 NEJM tested n=4 plasma samples[4]
- Hoffmann et al 2024 JCI tested n=2 plasma samples[5]
- Jarrell et al 2022 JACI n = 13 cases and n = 15 control plasma samples[6]

Another example is Figure 3f. How was this pregnancy time point chosen? Without a clear reason for this particular pregnancy time point, it may be better to show IL-1b levels changes in the same mouse at multiple time points? Is this one experiment with four mice per group? How many times was this experiment repeated? And how was this sample size chosen?

We have Il1b quantification from additional n=4 and n=4 anti-IL1RA isotype treated mice. This represents three independent experiments of n=7 isotype and n=8 anti-IL1RA treated mice. Each ELISA experiment was performed in technical duplicate, which is now clearly indicated in the figure legend.

The timepoint of murine anti-IL1RA and IL-1β administration (E13.5) was selected to model inflammatory perturbation after establishment of the definitive placenta, which occurs by approximately E12.5 in mice[7]. Administering cytokines at this stage minimizes confounding effects on implantation and early placental morphogenesis while allowing perturbation during a period of active maternal–fetal immune regulation. Prior studies have demonstrated that IL-1 signaling is sufficient to induce preterm parturition in murine models and that blockade of IL-1 signaling with IL-1 receptor antagonist can prevent inflammation-induced preterm birth[8,9]. This gestational window is therefore commonly used to model inflammation-driven pregnancy complications while avoiding disruption of early placental development.

Same with Figure 3s and 3t in that reproducibility is not clearly demonstrated. The text (lines 401-429) here refer to Extended data Figure 13, but this must be a typo (is Figure 14 intended?) which only show additional staining with individual samples. How were these particular samples chosen for this analysis?

We apologize for the typo, indeed we refer to Extended Data Figure 14. This has been corrected.

Anti-IL1RA localization in human placenta is replicated across 4 representative individuals. We tested for the presence of anti-IL1RA using the microscopy method in n=11 patients. The newly generated **Supplementary Table 6** further clarifies which samples were selected for the analysis. As with other orthogonal methods,

immunofluorescence staining utilizes relatively large amounts of serum and therefore samples were selected based on availability.

We do not replicate VE-cadherin staining as this exact staining pattern has already been well-established in the literature[10],which we now reference in our manuscript.

5. Spontaneous versus iatrogenic preterm birth. The description in the methods (lines 515-519) could use more specifics. If there was no spontaneous labor for iatrogenic preterm birth, then was labor induced? If so, what was the indication for induction? Is the data shown in Figure 3a-c for preterm birth restricted to spontaneous preterm birth? Are the few samples with high anti-IL-1RA antibodies from women with spontaneous or iatrogenic preterm birth?

We have added details into the methods that describe criteria for inclusion as iatrogenic preterm birth. We now state:

> Preterm pregnancies were defined as those delivering prior to 37 weeks of gestation and were further subdivided into spontaneous or iatrogenic preterm delivery. Spontaneous preterm pregnancies were defined as pregnancies with spontaneous labor with delivery prior to 37 weeks of gestation. Iatrogenic preterm pregnancies were defined as pregnancies with delivery prior to 37 weeks of gestation and without spontaneous labor, which included both induced vaginal and cesarean deliveries. Indication for iatrogenic preterm deliveries were variably documented across the eight cohorts and typically included: preeclampsia with severe features or concern for fetal/maternal well-being.

We include clinical features of anti-IL1RA positive preterm pregnancies in the new Supplementary Table 6. Overall, we are not powered to perform associations with clinical metadata variables for anti-IL1RA+ pregnancies. This limitation is clearly stated in the discussion:

> Furthermore, sample selection for orthogonal validation was determined by sample availability and the number of anti-IL1RA-positive pregnancies identified across cohorts remains underpowered to assess associations with clinical metadata variables.

We have clarified that Fig 3a-c includes all types of preterm birth.

6. These are just a few examples of instances throughout the paper where I think things are not well flushed out. As written, the paper covers a lot of breath, and pockets of in-depth analysis that are neither fully explained nor justified for the take home message of the paper for evidence of maternal autoantibodies in the pathogenesis of preterm birth.

We agree this paper covers a lot of breadth and utilizes a very large multi-center cohort of patients. Despite the breadth, the overall take home message is distilled in Figure 1 & 2, which together clearly show evidence of a maternal autoantibody signature associated not only with pregnancy, but also with preterm birth. This constitutes the primary take home message of the paper, and we follow it with carefully chosen investigations to elucidate possible mechanistic underpinnings. We chose IL1RA as one such candidate, and pursued it through a logical set of experiments, culminating with both *in vitro* and *in situ* human validation and *in vivo* validation in mice. Altogether, we believe this work follows a single linear thread of reasoning from discovery to validation, rather than pockets of disconnected investigations. With this revision, we have filled gaps in the original work and attempted to focus the narrative on this single thread.

REFERENCES:

1. Wang, E. Y. *et al.* High-throughput identification of autoantibodies that target the human exoproteome. *Cell Rep. Methods* **2**, 100172 (2022).

2. Zamecnik, C. R. *et al.* An autoantibody signature predictive for multiple sclerosis. *Nat. Med.* **30**, 1300–1308 (2024).

3. Pfeifer, J. *et al.* Autoantibodies against interleukin-1 receptor antagonist in multisystem inflammatory syndrome in children: a multicentre, retrospective, cohort study. *Lancet Rheumatol.* **4**, e329–e337 (2022).

4. Thurner, L. *et al.* IL-1RA Antibodies in Myocarditis after SARS-CoV-2 Vaccination. *N. Engl. J. Med.* **387**, 1524–1527 (2022).

5. Hoffmann, M.-C. *et al.* Autoantibody-Mediated Depletion of IL-1RA in Still's Disease and Potential Impact of IL-1 Targeting Therapies. *J. Clin. Immunol.* **44**, 45 (2024).

6. Jarrell, J. A. *et al.* Neutralizing anti-IL-1 receptor antagonist autoantibodies induce inflammatory and fibrotic mediators in IgG4-related disease. *J. Allergy Clin. Immunol.* **149**, 358–368 (2022).

7. Elmore, S. A. *et al.* Histology Atlas of the Developing Mouse Placenta. *Toxicol. Pathol.* **50**, 60–117 (2022).

8. Romero, R., Mazor, M. & Tartakovsky, B. Systemic administration of interleukin-1 induces preterm parturition in mice. *Am. J. Obstet. Gynecol.* **165**, 969–971 (1991).

9. Romero, R. & Tartakovsky, B. The natural interleukin-1 receptor antagonist prevents interleukin-1-induced preterm delivery in mice. *Am. J. Obstet. Gynecol.* **167**, 1041–1045 (1992).

10. Leach, L. The phenotype of the human materno-fetal endothelial barrier: molecular occupancy of paracellular junctions dictate permeability and angiogenic plasticity. *J. Anat.* **200**, 599–606 (2002).

Reviewer 1

This paper evaluates pregnancy induced protection of autoantibodies, and specifically focuses differences on the targets of autoantibodies between term and preterm pregnancies. This was accomplished analyzing 2700 sera samples from pregnant women using PhIP-seq. This analysis showed distinct term pregnancy and preterm birth antibody signatures. These results were somewhat verified using by mass spec evaluation of immunoprecipitated proteins from placental extracts for a limited subset of sera from term (n=13) and preterm (n=25) pregnancies showing 65% of the preterm birth signature proteins identified using PhIP-seq were also identified by mass spec. One of these targets for preterm birth autoantibodies in IL-1RA, and more focused analysis shows higher levels of antibodies targeting this protein in perterm pregnancies. The paper concludes with data showing pregnant mice treated with anti-IL-1RA antibodies have increased fetal resorption and other pregnancy defects.

I participated in the review for several prior versions of this manuscript for another journal. A consistent suggestion on each of these reviews was to improve the clarity, accessibility and accuracy of the writing (proofreading). Many parts of the text still contain specific technical details without explanation which makes it very hard to understand. Another consistent theme was that while evaluating the sera from 2700 pregnant women is commendable, the number of samples analyzed with preterm birth, and the rationale for how samples from term and preterm birth were chosen for more focused downstream analysis was not clearly described. I can see in the response to reviewers' comments that the authors made some superficial changes (eliminating the prior inappropriate references from the abstract; adding sample size to the abstract). Unfortunately, however these general concerns remain in this revised manuscript. While some data presented in the paper could be very interesting and helpful, the overall package is still very hard to understand, and as written probably more appropriate for a more specialized journal.

Specific comments-suggestions.

1. Line 133. What is the difference between a cross-sectional and population-based cohort design? Were the samples from males and control women that have never been pregnant from cohort 1 (the population based study?). Line 138, would evaluating sera from nulliparous women be a better control instead of nulligravid?

Population-based cohort designs aim to sample an entire population (in our case, births in the state of California). Cross-sectional studies sample individuals at a single time point or over a defined period to characterize exposures, outcomes, or traits within that population.

A population-based study can be cross-sectional if it captures a snapshot of the defined population. However, a cross-sectional study is not necessarily population-based, because it may sample only a selected clinical group, convenience sample, or other non-representative subset.

In the manuscript, we stated:

> “Male and nulligravid female sera from two cohorts of healthy individuals were used as **additional controls** (n=130).”

We have now clarified that this is a separate non-pregnant cohort. In lines 139-140, we state:

> “A separate cohort of sera from healthy males and nulligravid females was used as a non-pregnant control group (n=130; Supplementary Table 1).”

In previous revision of this work, we obtained a completely new cohort of non-pregnant healthy controls for which gravidity data was known. We utilize non-pregnant samples from nulligravidae and males to test whether the features selected from our frozen embryo transfer cohort can discriminate never-pregnant individuals from pregnant ones.

The assessment of the persistence of pregnancy autoantibodies in previously, but not currently pregnant individuals, is an interesting exploration outside the scope of our manuscript focused on pregnancy and adverse outcomes of pregnancy.

We address gravidity’s influence on the antibody signature throughout the manuscript (Figure 2b, Figure 3c).

2. Lines 144 and 145. When adding all the samples in Fig 1a, one gets 3101. Please explain the source of the 1411 (4512-3101) samples? Are these all from the 130 healthy non-pregnant individuals? If so this seems like a lot, and it may be helpful to explain why so many unique samples were obtained from these non-pregnant controls.

We have amended Supplementary Table 1 to describe all samples analyzed, including technical replicates.

In line 132 we now state:

> “To comprehensively investigate autoreactivity during pregnancy, maternal sera from term and preterm pregnancies derived from eight separate cohorts across three countries were used (n=3,101 samples derived from n=2,195 pregnant individuals). The cohorts captured cross-sectional (I, IV, V), longitudinal (II, III, VI–VIII), and population-based (I) designs (Fig 1a). Cohorts III and VI comprised only term pregnancies with dense sampling across gestation. Paired maternal–cord blood samples were available for cohorts III and IV (Extended Data Fig 1a-d; Supplementary Table 1).”

3. Line 149. I am not it is obvious what anti-GFAP antibodies are? I presume this is antibodies targeting Glial Fibrillary Acidic protein? This seems like a very obscure self antigen. Why were antibodies against the particular self-antigen chosen? Some background, rationale and explanation or even writing out this uncommonly used abbreviation would be helpful.

We state this is a positive control antibody. The identity of the target antigen is therefore irrelevant, however, GFAP has been used in a multitude of previous publications purely as a positive control for immunoprecipitation.

We have corrected the text in lines 166-167 as follows:

> “A commercial polyclonal antibody against glial fibrillary acidic protein (GFAP) was used as a positive control and replicated across 96-well plates, batches, and cohorts 88 times.“

4. Line 150. “(n=88)” cohorts. Is this a mistake since Fig 1A shows 8 pregnancy cohorts? If not, please explain how 8 pregnancy cohorts (plus samples from two non-pregnant [line 138] became 88.

We have corrected the text in lines 166-167 as follows:

> “A commercial polyclonal antibody against glial fibrillary acidic protein (GFAP) was used as a positive control and replicated across 96-well plates, batches, and cohorts 88 times”

We have now compiled the complete sample breakdown in Supplementary Table 1.

5. Lines 156-158. The 4512 samples described in line 158 is now reduced to 3101. Is having “under-sequenced samples” in over 30% of the samples expected? reasonable? Without more explanation on this point, I worry that the analysis on auto-antibodies is already biased on ~70% samples with autoantibodies to begin with. Are the “under-sequenced samples” more enriched for samples from preterm birth?

We have updated Supplementary Table 1 with an exact breakdown of technical replication and undersequencing.

In lines 176-177 we now state:

> “After quality control filtering to remove under-sequenced samples and averaging technically replicated samples (Supplementary Table 1; Supplementary Methods), a total of n=3,101 samples derived from n=2,195 pregnant subjects and n=130 samples from healthy non-pregnant controls were retained.”

The number 4512 originally reported included technical replicates which were averaged. The number of samples that were removed due to under-sequencing was 25, or 0.6% of the total samples. Undersequenced samples thus represent less than one percent and were not enriched for any clinical variable.

6. Lines 164-165. Why was this cohort chosen for initial analysis? My concern is that this choosing embryo transfer pregnancies will almost certainly not represent normal pregnancy. Did these women have embryo transfer pregnancy due to some underlying disorder? If so, would this bias the interpretation of results evaluating auto-antibodies?

This cohort was chosen because it is a model for human pregnancy for which we can obtain pre-pregnancy samples relative to the exact timing of human gestation. All pregnancies undergoing FET were patients seen at the infertility clinic. It is possible that they may have autoantibodies reflective of the underlying diagnoses.

However, we use this cohort to derive the initial features associated with pregnancy and then subsequently test on non-pregnant versus pregnant samples which encompass all types of pregnancy. This approach was chosen to specifically to avoid lack of generalizability.

We state:

> The classifier was then used to distinguish pregnant (n=2,719) from non-pregnant (n=130) samples from all cohorts except Cohort VI, which was used during feature selection only. The mean AUC of the receiver-operating-characteristic analysis was 0.93 (95% CI 0.91–0.96, $P = 5.3 \times 10^{-232}$; **Fig 1d**), supporting the existence of a large set of shared autoreactive pregnancy-associated changes. The PhIP-seq pregnancy signature recapitulated previously reported reactivities to histocompatibility molecules, pregnancy-associated proteins, and sperm-antigens (**Fig 1e**) and identified autoreactive targets not previously known to be pregnancy associated (**Extended Data Fig 3, Supplemental Table 2**). Together, these data define a complex yet highly shared set of autoreactive changes in the pregnant mother.

7. Line 167. More information is needed for evaluating how greater than or equal to 10 pregnancy samples was chosen. Cohort VI contains 13 pregnancies and 97 samples based on Fig 1a. How many of these pregnancies and how many of these 97 samples were included? For the criteria of “greater than or equal to 10 pregnancy samples” does it matter if the samples were primarily from the same person? Would it be helpful to expand the criteria to specifically indicate a minimum number of separate pregnant individuals are needed for defining a “pregnancy-specific peptide”. Here a distribution of data visually showing z-score 3 would also be really helpful.

All samples from Cohort VI were used to derive the features. We state:

> Pregnancy-specific peptides were defined as those with z-score ≥ 3, present in ≥ 10 pregnancy samples and absent from all baseline samples (**Fig 1c**).

We state that the peptides had to be absent from all baseline samples, which would be unique for each individual, and that they were present in at least 10 pregnancy samples. We do not need to extend the criteria for each separate pregnant individual, because we simply use this cohort to define peptides associated with pregnancy which we then **validate** in a larger set of pregnant versus non-pregnant samples.

We state:

> To derive the common signature of autoreactivity during normal pregnancy, the resulting 3,096 peptides, from 2,244 proteins, were used to build a logistic-regression classifier (Supplementary Methods). The classifier was then used to distinguish pregnant (n=2,719) from non-pregnant (n=130) samples from all cohorts except Cohort VI, which was used during feature selection only. The mean AUC of the receiver-operating-characteristic analysis was 0.93 (95% CI 0.91–0.96, $P = 5.3 \times 10^{-232}$; **Fig 1d**), supporting the existence

of a large set of shared autoreactive pregnancy-associated changes. The PhIP-seq pregnancy signature recapitulated previously reported reactivities to histocompatibility molecules, pregnancy-associated proteins, and sperm-antigens (**Fig 1e**) and identified autoreactive targets not previously known to be pregnancy associated (**Extended Data Fig 3, Supplemental Table 2**). Together, these data define a complex yet highly shared set of autoreactive changes in the pregnant mother.

Throughout the manuscript we use the word “subjects” to refer to unique individuals and “samples” to refer to samples from individuals, which may not be unique to an individual.

8. Line 171-172. I am not sure that most readers of Nature immunology will understand what “mean AUC of the receiver-operating-characteristic analysis” means. I think the whole paper would read a lot better if these complex words are explain or at least used in context. Without it, I worry this is essentially a meaningless sentence and piece of data.

AUC (Area under the curve) of ROC (receiver operator characteristic) are terms common to statistical analysis and well known to the readers of *Nature Immunology*.

Here are four recent papers published in *Nature Immunology* with ROC analyses:

https://www.nature.com/articles/s41590-022-01329-5

https://www.nature.com/articles/s41590-023-01590-2

https://www.nature.com/articles/s41590-025-02353-x

https://www.nature.com/articles/s41590-026-02478-7

We provide extensive documentation of our statistical analysis approach in the Supplementary Methods.

9. Line 184. I am not sure that most readers of Nature immunology will understand what “mean RPK values at the peptide level” means. See additional comments for preceding point 8.

The RPK measure has become a standard way to normalize for the number of read counts, when the total number of reads for any given library may differ. Normalizing sequencing data is standard practice essentially all applications of deep sequencing. All terms, including RPK, are well documented in our supplementary methods.

We believe this is an entirely appropriate use of this term for the audience.

10. Line 187. Here preterm associated peptide are defined based on the presence in a minimum number of subjects (but previously it was based on number of samples without consideration for how many subjects?) See preceding point 7. “At least four preterm subjects” out of how many?

We derived the pregnancy signature and the preterm signature differently.

Throughout the manuscript we use the word “subjects” to refer to unique individuals and “samples” to refer to samples from individuals, which may not be unique to an individual.

Here we use the term “subject”, indicating that we required that 4 preterm individuals had a reactivity to the specific peptide.

A dedicated Extended Data Figure 4 explains how this peptide set was chosen in addition to detailed methods in the Supplementary Methods section.

11. Lines 207 to 208. How were these peptides chosen? I assume this refers to peptides among the 9209 preterm-specific peptides described in lines 190-191? Where these the top 10 and top 500? If so, this should be clearly stated, and how differences in preterm peptide priority is assigned clear explained (is this based on difference magnitude between term pregnancies?, P value?)

This is defined in the figure legend (Figure 2b) and the supplementary methods:

> Logistic regression analyses were performed using the earliest available gestational sample per patient, deduplicated by patient identifier to ensure statistical independence. For each sample, the cumulative PhIP-seq enrichment score was computed as the sum of $\log_2$ fold changes (preterm over healthy term) across peptides in the preterm autoantibody signature. **Scores were calculated at multiple stringency levels by restricting to the top 10, 50, 100, 200, or 500 peptides ranked by fold change, as well as across all signature peptides.** All PhIP-seq scores and continuous clinical variables (maternal age, gravidity) were standardized to zero mean and unit variance prior to modeling, so that odds ratios reflect the effect of a one standard deviation increase.
>
> Separate logistic regression models were fit for each clinical variable (history of prior preterm birth, preeclampsia, maternal age, and gravidity) and each PhIP-seq enrichment score, with preterm birth as the binary outcome. Odds ratios and 95% Wald confidence intervals were computed from the estimated coefficients.
>
> A multivariate logistic regression model was fit including both history of preterm birth and the PhIP-seq top-100 peptide score as predictors. To estimate the combined effect of both risk factors, we computed the joint log-odds for a patient with a positive history of preterm birth and elevated autoantibody burden at +1 and +2 standard deviations above the mean, with confidence intervals computed using the delta method to account for covariance between parameter estimates. Results were visualized as a forest plot of odds ratios with 95% confidence intervals. All models were fit using the statsmodels Python package.

12. Figures 2. I think another very important consideration missing in these analysis is consideration of preterm birth not as binary variable, but rather a continuous variable.

We did consider preterm birth as a continuous variable. This analysis is in Figure 3b, Figure 5d, Extended Data Figure 8 where we examine the severity of the autoreactive signature with the gestational age at preterm delivery.

13. Line 224. Is “severe” preterm birth the same as “early (Fig 3b)” preterm birth?

Yes. When read in the complete context of the sentence, it corresponds with the definition also provided in the figure legend.

In the text we state:

> Furthermore, summed enrichment of the preterm autoantibody signature was significantly greater **in more severe preterm deliveries (before 28 weeks) as compared to moderate or late preterm deliveries** (Fig 3b).

In the figure legend we state:

> Summed log10 fold change over mock IP of the preterm-specific autoantibody signature in preterm pregnancies stratified by b. **early (<28 weeks),** moderate (28-32 weeks), and late (33-37 weeks) delivery age groups

14. Line 232-235. Differences between spontaneous vs iatrogenic preterm birth is specifically called out as being “modestly higher” compared with the order comparisons (C-section, infant sex, history of preterm birth, preeclampsia), yet the P value for this is the highest (least statistically significant, P=0.6 [Extended data figure 6]). Please explain.

We added “though this did not reach significance” at the end of the sentence in question.

In lines 275-276 we now state:

> “Spontaneous preterm pregnancies exhibited a modestly higher preterm autoantibody signature enrichment than iatrogenic preterm pregnancies, though this did not reach statistical significance (Extended Data Fig 6c).”

15. Line 243. Please show when the gestational age for when these samples were collected (similar to the data in Fig 1b), but just for this smaller subset.

We can’t find what the reviewer is referring to here, as the line number does not seem to correspond to their request. We investigate gestational age at sample collection in Figure 3a, Extended Data Figure 6a-b.

16. Line 302. I could not find IL1R2 in Fig 6a. Is this a typo?

This was indeed a typo. We have corrected to say IL2RB

17. Line 304-305. Please clearly state which specific proteins that are “essential” and expression on the placenta refers to, with corresponding references.

We provide references as we describe each protein. In the manuscript we state:

> Several critical reproductive hormones (and/or their receptors) were significantly enriched (CSH2, CGB2, CGB7, PGF). Vascular remodeling of maternal spiral arterioles is a hallmark of successful placentation[42]. Accordingly, proteases (e.g. MMP11, MMP7) and canonical angiogenesis proteins (e.g. LAMB3) were significantly targeted in preterm pregnancies (**Fig 6a**).
>
> Preterm autoantibody targets also included histocompatibility and antigen processing proteins (B2M, HLA-DPB1, HLA-DQA2, MICA, MICB, PSMB9) and the placental-specific HLA-G (**Fig 6a**). Anti-HLA antibodies increase the risk of spontaneous preterm delivery[31] and are associated with complement deposition in the placental endothelium, which occurs more frequently in spontaneous preterm labor[32]. Anti-cytokine antibodies, known to increase susceptibility to infection[43], were also identified. Of these, anti- interleukin-1 receptor antagonist (IL1RA) and anti-IL1R2 were the most significantly enriched. Furthermore, anti-IL1RA was the only anti-cytokine reactivity that was detected both by PhIP-seq and IP-MS (**Fig 6a**; **Supplementary Table 3; Supplementary Table 4**). Together, the essential roles of these proteins, their expression on placenta, and their enrichment by preterm sera suggests that beyond a breakdown in tolerance, there is the possibility that some of the corresponding autoantibodies may possess pathologic function-blocking activities related to pathways that maintain successful pregnancy.

18. Line 319. I am not sure reference 13 alone from 20 years ago showing intraamniotic injection of purified IL-1 in nonhuman primates means that the role for this cytokine in pregnancy is “well-established”. This is specifically in contrast to other parts of the paper (line 103) where “Little is known” used to describe the causes of preterm labor where I would argue just as much is known vs unknown.

In the manuscript we state:

> “**<u>Although little is known about the causes of preterm labor</u>, animal studies have demonstrated that certain inflammatory cytokines are sufficient to induce labor[13–16]**. Intrauterine injection of inflammatory cytokines, particularly IL1β, rapidly induces labor in non-human primates[13,17–20] and rodents[21–25]. This finding mirrors associations in humans, where elevated inflammatory cytokines are detected in mothers during labor, including spontaneous preterm labor[1–5,26]."

We cite many articles where injection of inflammatory cytokines particularly IL1b induces labor or reproductive defects:

> Intrauterine injection of inflammatory cytokines, particularly IL1β, rapidly induces labor in non-human primates[13,17–20] and rodents[21–25].

The large body of evidence from both humans and animal experiments, spanning decades, has established the importance of this cytokine in the context of pregnancy.

19. Figure 6c and Figure 6d. Please clearly state when the samples were collected for each of the term and preterm birth samples, and whether these preterm births were early/severe, moderate or late.

In the figure legend we state the cohort:

> c. protein counts detected by placental IP-MS in Cohort II, or d. net MFI of IL 1RA over BSA detected by Luminex assay in Cohort V.

We have already constructed Supplementary Table 6 which contains all of the requested information.

We have modified the text as follows:

> To assess the prevalence of anti-IL1RA antibodies, two orthogonal approaches were used. First, immunoprecipitation-mass spectrometry (IP-MS) was used to interrogate Cohort II sample (20–30 weeks gestation at sample collection; randomly sampled n=25 preterm, n=13 term). This analysis recovered antibody bound IL1RA in 16% of preterm samples (Fig 6c; Supplementary Table 6). In an independent cohort (Cohort V; 11–15 weeks gestation at sample collection; n=65 preterm, n=69 term), we implemented a Luminex assay which detected anti-IL1RA in 6% of preterm pregnancies and <1.5% of term pregnancies (Fig 6d). We further profiled a random sample of 894 longitudinal sera from Cohort VIII (n=86 preterm, n=808 term) using the same Luminex platform. Prevalence of anti-IL-1RA positivity in preterm pregnancies was 2.3% before 18 weeks (n=3/129). In contrast, term pregnancies exhibited ≤0.4% prevalence before 18 weeks (n=2/516; Fisher exact test OR= 6.12, p=0.057). We also noted anti-IL-1RA positivity among term pregnancies at later gestational ages, including 2.5% after 18 weeks (n=4/90; Extended Data Fig 9a-c; Supplementary Table 6). Together, these results support a model in which early emergence, rather than presence alone, is associated with increased preterm risk.

20. Lines 342-343. Are the antibodies equally effective at pickup on different versions of this peptide based on differential glycosylation? If not, is it possible that there are differences in antibodies to IL-1RA specifically reflect one (glycosylated) variant of this glycoprotein? This should be specially evaluated.

The glycosylation status of IL-1RA in these patients is unknown, and thus whether patient autoantibodies may differentiate glycosylation status is also unknown. This facet of autoimmunity is complex and worthy of future study, but is beyond the scope of this manuscript.

21. Line 356. Which specific samples (from which cohorts, and levels of anti-IL-1RA and based which assay showing anti-IL-1RA activity) is this referring. Figure 6f would be a lot more meaningful if preterm samples with and without anti-IL1RA were directly compared, and samples from term pregnancies with and without anti-IL1RA were directly compared.

This is provided in Supplementary Table 6. Figure 6f is intended to demonstrate that anti-IL1RA-positive preterm sera possess functional neutralizing activity relative to controls: a large, robust effect (Mann–Whitney p=0.001 vs term, p=0.0002 vs nulligravid; Kruskal–Wallis $p=7\times10^{-5}$), corroborated by IC50 titration of the patient epitope targeting antibody (new to this revision). A within-outcome subdivision by antibody status would require additional serum, which is exhausted for these retrospective cohorts.

22. Lines 381, 384, 387. Is this referring to figure 7 rather than figure 3?

The typo has been corrected.

23. Extended data Figure 7. Would it be helpful to also analyze the data in Extended data Figure 6 using the same parameters (comparing with term pregnancy?)

We demonstrate the effect of gestational age at sampling within term pregnancy in Extended Data Fig 6b.

We cannot add term pregnancy to Extended Data Fig 6c, as this is a graph of the type of preterm delivery relative to the preterm autoantibody signature, which necessarily excludes term pregnancy.

For other clinical covariates (infant sex, mode of delivery, preeclampsia, prior history of preterm birth), we tested whether the signature differed between covariate levels separately within the term and within the preterm group, using both a two-sample Kolmogorov-Smirnov test and one-way ANOVA. Because this entails multiple comparisons, all p-values were corrected using the Benjamini-Hochberg FDR.

After FDR correction, no covariate was significantly associated with the signature in either the term or the preterm group (all $q > 0.1$). The nominally strongest association (infant sex within the preterm group; ANOVA $p = 0.013$) did not survive correction ($q = 0.10$):

- Infant sex (Female vs. Male): term ANOVA $p = 0.12$ ($q = 0.47$); preterm $p = 0.013$ ($q = 0.10$)
- Cesarean section (No vs. Yes): term $p = 0.36$ ($q = 0.57$); preterm $p = 0.26$ ($q = 0.57$)
- Preeclampsia (No vs. Yes): term $p = 0.96$ ($q = 0.96$); preterm $p = 0.33$ ($q = 0.57$)
- History of preterm birth (No vs. Yes): term $p = 0.47$ ($q = 0.62$); preterm $p = 0.73$ ($q = 0.84$)

The two-sample KS tests were concordant.

In contrast, the difference between term and preterm pregnancies was highly significant across every stratification (two-way ANOVA main effect of outcome, $p < 1e-5$), confirming that the signature reflects preterm status and not these covariates.

Because none of these covariates modulates the signature within either group, we have chosen not to add the term-pregnancy arm to the figure itself: doing so would substantially increase the

number of panels/boxes and, in our view, distract from the main message without conveying additional information.

23. Extended data Figure 8F (right sided). Percent primigravida and percent nulliparous in this context comparing the samples from pregnant women seem like the same thing? Should nulliparous be "multiparous"? or is this referring to the state of these women prior to the current pregnancy?

“Nulliparous” is not intended to mean “multiparous.” Both variables describe reproductive history before the current pregnancy. Primigravida participants had no previous pregnancies, whereas nulliparous participants had no previous pregnancies resulting in a birth; therefore, the nulliparous category may also include women with prior pregnancies that ended before delivery. We have clarified this distinction in the figure legend.

Reviewer #2

(Remarks to the Author)
The paper by Rackaityte et al. studies the presence of maternally transferred autoantibodies and it’s association with preterm birth.

The authors perform a proteome wide screening using PhiPSeq on several thousand pregnant women.

Interestingly, they identify autoantibodies to IL1RA (a negative regulator of IL1b) as enriched in preterm pregnancies. They convincingly show the impact of IL1RA in mice and its colocalization to endothelial adherens in human placenta.

Comments:
- Discussion about immunosuppressive therapies in women with recurrent preterm pregnancies would be interested

We agree that this is an intriguing question raised by our work. Indeed, we demonstrate in Figure 2b that previous preterm birth significantly increases risk of subsequent preterm birth and that this risk is significantly elevated if combined with our preterm autoantibody signature. Supporting this, we further show that greater number of previous pregnancies (gravidity) results in a significantly higher preterm autoantibody signature (Figure 3c).

In lines 680-693 we now state:

> Beyond an individual protein, these collective proteome-wide data, representing thousands of individuals, reveals that pregnancy itself elicits a broad and shared program of humoral autoreactivity, and that preterm birth is distinguished by a markedly expanded signature targeting proteins essential for placental function, vascular remodeling, and immune regulation. The enrichment of autoreactivity with gravidity further suggests that

repeated pregnancy may shape the maternal humoral response to fetal, placental, or pregnancy-associated antigens. Supporting this, prior pregnancy may not simply represent a clinical risk factor, but an immunological exposure history that alters the baseline state from which subsequent pregnancies begin. The breadth of this landscape suggests that distinct autoantibody-defined subtypes of preterm birth exist, each potentially amenable to serologic identification and targeted intervention. In women with recurrent preterm birth and evidence of pathogenic humoral autoreactivity, these findings may ultimately support evaluation of selective immunomodulatory or immunosuppressive strategies, although such approaches will require careful mechanistic and clinical validation. Together, these advances may yield improved management of pregnancies at risk for preterm delivery and reductions in neonatal morbidity and mortality.

- Have the authors considered studying still births, which would be seen as an extreme phenotype

We are engaged in a number of additional studies investigating various other outcomes of pregnancy, including still birth and hydrops fetalis. Such investigations are outside the scope of this work.

- The identification of Abs against HLA is interesting, but not much discussed. Do the authors have the HLA of the babies who were born to such mothers. Indeed, a matching of HLA antibodies targeting an HLA of the fetus would be very interesting

The reviewer's specific suggestion of matching a mother's anti-HLA specificity to her own fetus's HLA alleles is compelling but requires paired neonatal HLA genotyping, which was not collected for these retrospective cohorts and cannot be recovered from the banked maternal serum used here. We therefore cannot perform fetal-directed epitope matching in this study. We now state this limitation explicitly and identify prospective maternal–fetal HLA typing, paired with anti-HLA antibody specificity mapping, as a defined future direction.

In lines 695-719 we now state:

> "More broadly, this study identifies a proteome-wide humoral immune program elicited by pregnancy, as well as distinct, shared changes preceding preterm delivery. The reprogramming of autoreactivity across gestation, the cumulative effect of gravidity, the enrichment of histocompatibility and antigen-processing targets, and the existence of antibodies against cytokine regulators together suggest that maternal B cell responses are dynamically shaped by fetal, placental, and inflammatory antigen exposure. Notably, antibodies targeting HLA proteins may reflect maternal alloimmune responses to paternally inherited fetal HLA antigens. Fetal HLA genotypes were not available for these cohorts, precluding direct assessment of whether maternal HLA antibodies matched fetal alleles; paired maternal antibody and fetal HLA profiling will be important in future studies to establish the specificity and potential functional relevance of these responses. In this context, preterm birth-associated antibodies may represent not only biomarkers of disease risk, but also molecular evidence of disrupted maternal-fetal immune tolerance. Some reactivities may reflect failed tolerance to pregnancy-associated antigens, others may

represent alloimmune responses to fetal or placental variants, and still others may arise secondary to tissue injury or infection. The convergence of these signals on pathways involved in antigen presentation, vascular integrity, placental remodeling, and IL-1β regulation suggests that humoral immunity may participate in multiple mechanistic routes to early parturition. "

- A major caveat of PhiPSeq is that it can only study peptides, and not confirmational proteins. The authors should discuss this important issue.

The PhIPseq tendency to detect largely linear epitopes is discussed in the results but more importantly, it was this limitation that motivated us to include the mass spectrometry work, which utilizes full length human protein from placental extract as the input, and is considered the gold standard for identifying proteins in immunoprecipitations. The overlap between the mass spectrometry and our PhIPseq data is discussed and presented in Figure 4. With respect to IL-1RA, this protein was prominent in the mass spectrometry data, further motivating our follow-up work on this hit.

- Neutralization shown in figure 6f is far from convincing, although this should be the most important confirmation that these autoantibodies to IL1RA are functional. I would suggest optimizing the technique to clearly show the neutralizing patients or consider using other assays.

Cell-based functional assays using the HEK-Blue IL-1β reporter line are well established in the literature (for example, references 45, 46, 47 in the manuscript) and widely used. A key difference is that we assess anti-IL1RA neutralization using polyclonal patient serum rather than monoclonal antibodies or small molecules. Another limitation of these assays is the requirement for large amounts of serum input, and for these cohorts, serum availability is always a limiting factor.

To address this point, we have expanded our analysis and include new experiments, described below.

First, we have re-analyzed the expanded serum panel ($n = 10$ nulligravid, $n = 19$ term, $n = 6$ anti-IL1RA-positive preterm) using non-parametric statistics. Residual IL-1β reporter signal, the inverse of serum IL-1RA activity, was significantly higher in anti-IL1RA-positive preterm sera than in term sera (Mann–Whitney $p = 0.001$; Cohen's $d = 1.9$) and nulligravid sera ($p = 0.0002$; $d = 2.4$), and increased monotonically across nulligravid < term < preterm groups (Kruskal–Wallis $p = 7 \times 10^{-5}$; ordered-trend $p < 1 \times 10^{-5}$). These are large, statistically robust effects.

Second, we include a new IC50 neutralization assay of an antibody raised against the same epitope as recognized by the patient antibodies (Figure 6e, Extended Data 9d). This clearly shows that the antibody is an efficient neutralizer of IL1RA antagonism. This was the same antibody we injected in mice, where we investigated pregnancy phenotypes.

Third, this *in vitro* assay is one of five orthogonal methods (PhIP-seq, IP-MS, Luminex, cell-based reporter, and immunofluorescence blocking) that independently detect anti-IL1RA activity in preterm sera, reported in Supplementary Table 6.

Fourth, and most importantly, the definitive test of function is our *in vivo* experiment: administration of anti-IL1RA antibody to pregnant mice (three independent experiments, n = 7 isotype, n = 8 anti-IL1RA) reproduces IL-1-dependent pregnancy pathology, demonstrating that neutralization of IL-1RA is functionally consequential in a living system. The cell-based assay corroborates this mechanism at the level of human serum.

We have (i) added the IL-1RA:IL-1β titration and IC50 analysis (purified antibody and isotype/no-treatment controls) as a new panel and (ii) updated the results with the statistics above.

- What is the impact of IL1RA AABs on systemic inflammation in these children and their mothers?

We do not have systemic inflammatory profiling (e.g., serial cytokine measurements) on the anti-IL1RA-positive mothers and their children.

Whether this manifests as measurable systemic inflammation in mothers or offspring is an important question that requires prospectively banked inflammatory markers. We are assembling a large prospective cohort designed to test exactly this.

We state in the Discussion, lines 665:

> “the number of anti-IL1RA-positive pregnancies identified across cohorts remains underpowered to assess associations with clinical metadata variables.”

- Did any of the positive mothers have a particular clinical phenotype or disease ? Same question for the children.

Supplementary Table 6 compiles all available clinical metadata for anti-IL1RA-positive subjects. We did not identify a single recurring maternal diagnosis: notably, none of the anti-IL1RA-positive mothers with recorded preeclampsia status were preeclamptic, indicating that the reactivity is not a proxy for the classic hypertensive/inflammatory preterm phenotype. Among positives with a documented preterm subtype, the majority were spontaneous rather than iatrogenic preterm births, consistent with an inflammation-associated mechanism. Systematic clinical follow-up data on the children from this study are not available.

We state in the Discussion, lines 665:

> “Furthermore, sample selection for orthogonal validation was determined by sample availability and the number of anti-IL1RA-positive pregnancies identified across cohorts remains underpowered to assess associations with clinical metadata variables.”

- The prevalence of anti-IL1RA autoantibodies need to be clarified among the different groups, given the small number of patients. i.e. how many positive individuals within the preterm versus term babies.

We have clarified group-wise prevalence in Supplementary Table 6 and in the text. By every antibody-detection method except Luminex, anti-IL1RA positivity is confined to or strongly enriched in preterm pregnancies (PhIP-seq 1.5% preterm vs 0% term; IP-MS 16% vs 0%; cell-based reporter 35% vs 0%; immunofluorescence 57% vs 0%). Prevalence estimates vary by assay by design and the preterm-specific enrichment is consistent across the orthogonal conformational and functional methods.

In lines 479, we state:

> "To assess the prevalence of anti-IL1RA antibodies, two orthogonal approaches were used. First, immunoprecipitation-mass spectrometry (IP-MS) was used to interrogate Cohort II sample (20–30 weeks gestation at sample collection; randomly sampled n=25 preterm, n=13 term). This analysis recovered antibody bound IL1RA in 16% of preterm samples (Fig 6c; Supplementary Table 6). In an independent cohort (Cohort V; 11–15 weeks gestation at sample collection; n=65 preterm, n=69 term), we implemented a Luminex assay which detected anti-IL1RA in 6% of preterm pregnancies and <1.5% of term pregnancies (Fig 6d). We further profiled a random sample of 894 longitudinal sera from Cohort VIII (n=86 preterm, n=808 term) using the same Luminex platform. Prevalence of anti-IL-1RA positivity in preterm pregnancies was 2.3% before 18 weeks (n=3/129). In contrast, term pregnancies exhibited ≤0.4% prevalence before 18 weeks (n=2/516; Fisher exact test OR= 6.12, p=0.057). We also noted anti-IL-1RA positivity among term pregnancies at later gestational ages, including 2.5% after 18 weeks (n=4/90; Extended Data Fig 9a-c; Supplementary Table 6). Together, these results support a model in which early emergence, rather than presence alone, is associated with increased preterm risk."
